# Miro1 Protects Against Acute Kidney Injury Through Modulating Mitochondrial Homeostasis via Interaction With Sirt6

**DOI:** 10.1002/advs.76824

**Published:** 2026-08-05

**Authors:** Lin Wu, Qing Li, Yiyang Xu, Zitao Wang, Hua Shu, Yangfan Wu, Fang Lu, Zhimin Huang, Bo Zhang, Hongwei Liang, Changying Xing, Huijuan Mao, Yanggang Yuan

**Affiliations:** ^1^ Department of Nephrology the First Affiliated Hospital of Nanjing Medical University Nanjing Medical University Nanjing China; ^2^ School of Life Science and Technology China Pharmaceutical University Nanjing Jiangsu China

**Keywords:** acute kidney injury, Miro1, mitochondrial movement, Sirt6

## Abstract

Acute kidney injury (AKI) is highly prevalent worldwide but lacks specific treatments. Mitochondrial dysfunction plays a central role in its pathogenesis, making mitochondrial protection a promising therapeutic strategy. Miro1 is a key regulator of mitochondrial dynamics, yet its involvement in AKI remains unexplored. In this study, we found that Miro1 expression was significantly downregulated in renal tubular cells from AKI patients and in multiple murine AKI models. Functional studies demonstrated that Miro1 loss aggravated tubular cell injury and mitochondrial dysfunction, whereas Miro1 overexpression exerted protective effects. Mechanistically, we identified Sirt6 as a novel interacting partner of Miro1. Sirt6 directly deacetylated Miro1 at lysine residue K182, which inhibited its ubiquitin‐proteasome degradation and maintained Miro1 protein stability. Disruption of Sirt6 activity led to enhanced Miro1 downregulation and increased tubular cell apoptosis, while Sirt6 activation reversed these detrimental effects in a deacetylase‐dependent manner. Reciprocal rescue experiments further confirmed that Miro1 serves as a critical downstream effector of Sirt6. Collectively, our findings uncover the Sirt6–Miro1 axis as a previously unrecognized protective mechanism in AKI, wherein Sirt6 stabilizes Miro1 via deacetylation to preserve mitochondrial homeostasis and alleviate kidney injury. This axis may represent a novel therapeutic target for AKI intervention.

## Graphical abstract

Ischemia‐reperfusion reduces Sirt6 activity, thereby increasing Miro1 acetylation. Hyperacetylated Miro1 exhibits perinuclear distribution and degradation, thereby inducing mitochondrial dysfunction and promoting apoptosis in renal tubular epithelial cells. This pathway reveals a mechanistic link between Sirt6‐mediated deacetylation and Miro1 stability in ischemic kidney injury.

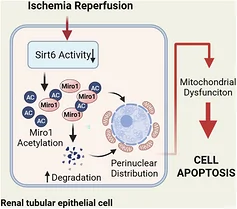

## 1 introduction

Acute kidney injury (AKI) is associated with high morbidity and mortality [1]. The primary etiologies of AKI include sepsis, ischemia/reperfusion (IR) injury, and nephrotoxic agents [2]. The pathogenesis of AKI is multifactorial, involving multiple cell types, among which damage to the tubular epithelial cell has been demonstrated to be the main driving force in AKI development [3]. However, the precise molecular mechanisms underlying tubular cell injury remain unclear.

Mitochondrial damage has been implicated as a key element in the pathogenesis of tubular cell injury in AKI [4]. Mitochondrial dynamics maintain the mitochondrial structure and control mitochondrial function [5]. The mitochondrial dynamics include fusion, fission, movement, biogenesis, and mitophagy [6]. Given the high‐energy demand and extremely polarized structures of neurons, appropriate distribution of mitochondria and maintenance of mitochondrial dynamics are crucial for the function and survival of neurons [7]. Mitochondrial transport between the soma and distal axonal, leading to altered mitochondrial distribution [8]. The defects in mitochondrial movement may affect mitochondrial distribution at the cell periphery [9]. Renal tubular epithelial cells, like neurons, are highly polarized cells with substantial energy requirements, depending primarily on mitochondrial fatty acid oxidation (FAO) for ATP production to support active transport and reabsorptive functions [10]. Mitochondrial dysfunction is a hallmark of AKI [11], and proper mitochondrial positioning is critical for maintaining tubular cell energetics and survival. Thus, strategies for improving mitochondrial distribution may also be a promising therapeutic target in AKI.

Mitochondrial trafficking is mediated via ATP‐dependent molecular motors that transport mitochondria via microtubules [12]. In mammalian cells, mitochondrial transport is facilitated by mitochondrial membrane proteins Miro to link kinesin and dynein motors via trafficking kinesin‐binding protein 1 (TRAK1) and trafficking kinesin‐binding protein 2 (TRAK2) [13]. Miro proteins are evolutionarily conserved mitochondrial calcium‐binding GTPases that regulate various mitochondrial processes [14]. Two Miro genes exist in mice and humans: ras homolog family member T1 (RHOT1), which encodes Miro1, and ras homolog family member T2 (RHOT2), which encodes Miro2. Their functional differences are still unclear [15]. Miro1 is the most reported regulator of mitochondrial transport in neurons and other cell types [16]. However, its precise roles and the mechanisms underlying its involvement in AKI remain elusive.

In this study, we discovered that the levels of Miro1 were reduced in renal biopsies from acute tubular necrosis (ATN) patients and AKI models both in vivo and in vitro. Miro1 protected against tubular cell injury in cisplatin or IR‐induced AKI. Mechanistically, we performed an unbiased Shotgun Proteomics screen for Miro1‐interacting proteins and identified Sirt6, an NAD^+^‐dependent class III deacetylase, as a specific binding partner of Miro1 in renal tubular cells. Notably, while Sirt6 is canonically recognized as a nuclear protein with well‐documented renoprotective functions in various AKI models [17], its cytoplasmic interaction with a mitochondrial outer membrane protein such as Miro1 had not been previously reported. This finding prompted us to investigate whether Sirt6 regulates Miro1 through direct deacetylation and whether this novel Sirt6‐Miro1 axis contributes to mitochondrial protection during AKI.

## 2 Results

### 2.1 Miro1 Protein Levels are Strongly Decreased in AKI

Given that Miro1 and Miro2 are paralogs within the same Miro family, we initially employed single‐cell RNA sequencing to examine their gene expression profiles in a murine renal ischemia‐reperfusion injury (IRI) model across various time points and pathological stages. As depicted in Figure S1A,B, the transcript levels of both Miro1 and Miro2 progressively declined with prolonged reperfusion time. In comparison to normal control and repaired tubular cells, both injured and unsuccessfully repaired tubules exhibited markedly reduced expression of Miro1 and Miro2. However, Miro1 expression was consistently much higher than Miro2 under all tested conditions, leading us to select Miro1 for further mechanistic studies.

To investigate the role of Miro1 in AKI, we first assessed the expression of Miro1 in patients with ATN. These results indicated that the levels of Miro1 protein were decreased in patients with ATN when compared to minimal change disease (MCD) patients (Figure 1A,B). We next examined whether Miro1 levels were associated with conventional renal function parameters. Among these, serum creatinine (Scr) and blood urea nitrogen (BUN) were selected as representative indicators of kidney function for correlation analysis. Notably, Spearman correlation analysis revealed a negative correlation between Miro1 levels and Scr (r = −0.786, *p* < 0.01) as well as BUN (r = −0.600, *p* < 0.05) (Figure 1C,D). Then, we examined Miro1 expression in the kidneys obtained from two well‐characterized AKI mouse models induced by IRI and cisplatin administration, respectively. After IRI or cisplatin injection, Miro1 protein levels were decreased in the diseased kidneys compared to sham mice (Figure 1E,F). Additionally, immunohistochemical staining showed that Miro1 was consistently reduced in the renal tubular cells (Figure 1G,H).

**FIGURE 1:**
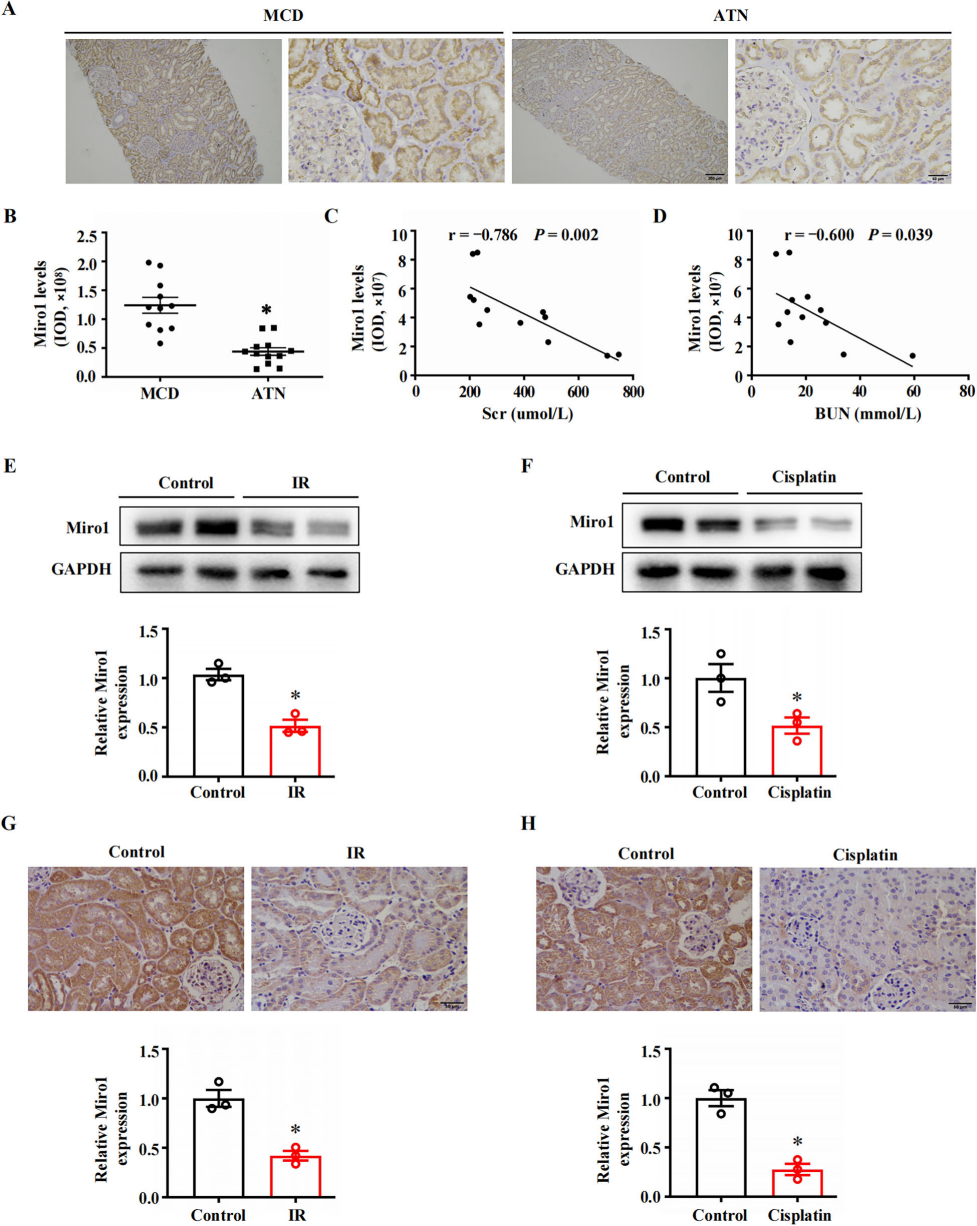
Miro1 levels decreased during AKI. (A) A representative photomicrograph of Miro1 immunohistochemical staining in renal biopsy tissue from patients with ATN (n = 12) and patients with MCD (n = 11) as the control. Scale bar of low magnification: 200 µm; Scale bar of high magnification: 50 µm. (B) Relative protein levels of Miro1. Data are presented as means ± SEM. ^∗^ *p* < 0.05 vs. MCD. Negative correlation between Miro1 protein levels and Scr (C) and BUN (D) levels in ATN patients. (E) Western blotting and densitometry analysis of Miro1 in the kidney cortex of control and IR‐induced mice. (F) Western blotting and densitometry analysis of Miro1 in the kidney cortex of control and cisplatin‐induced mice. (G) The immunohistochemical staining and densitometry analysis of Miro1 in kidney tissue of control and IR‐induced mice. Scale bar: 50 µm. (H) The immunohistochemical staining and densitometry analysis of Miro1 in kidney tissue of control and cisplatin‐induced mice. Scale bar: 50 µm. Data are shown as means ± SEM (n = 3). ∗*p* < 0.05 vs. control group.

We next extended our analysis to evaluate Miro2 protein expression in both in vivo and in vitro AKI models. Miro2 levels were significantly decreased in mouse IR and cisplatin models (Figure S1C,D), as well as in HK2 cells treated with CoCl_2_ or cisplatin (Figure S1E,F).

### 2.2 Knockdown of Miro1 Exacerbates Renal Tubular Cell Injury In Vitro

Because tubules are the epicenter of damage after AKI, we then examined if Miro1 knockdown affects tubular cell death in cultured HK2 cells. Miro1 was silenced by siRNA transfection (Figure 2A). Knockdown of Miro1 in HK2 cells increased CoCl_2_‐induced cleaved caspase‐3 expression (Figure 2B). Flow cytometry results show that Miro1 knockdown aggravated CoCl_2_‐induced apoptosis (Figure 2C,D). Consistently, Miro1 knockdown also aggravated apoptosis in HK2 cells induced by cisplatin (Figure 2E,F). These data indicated that the knockdown of Miro1 exacerbated acute renal tubular cell injury.

**FIGURE 2:**
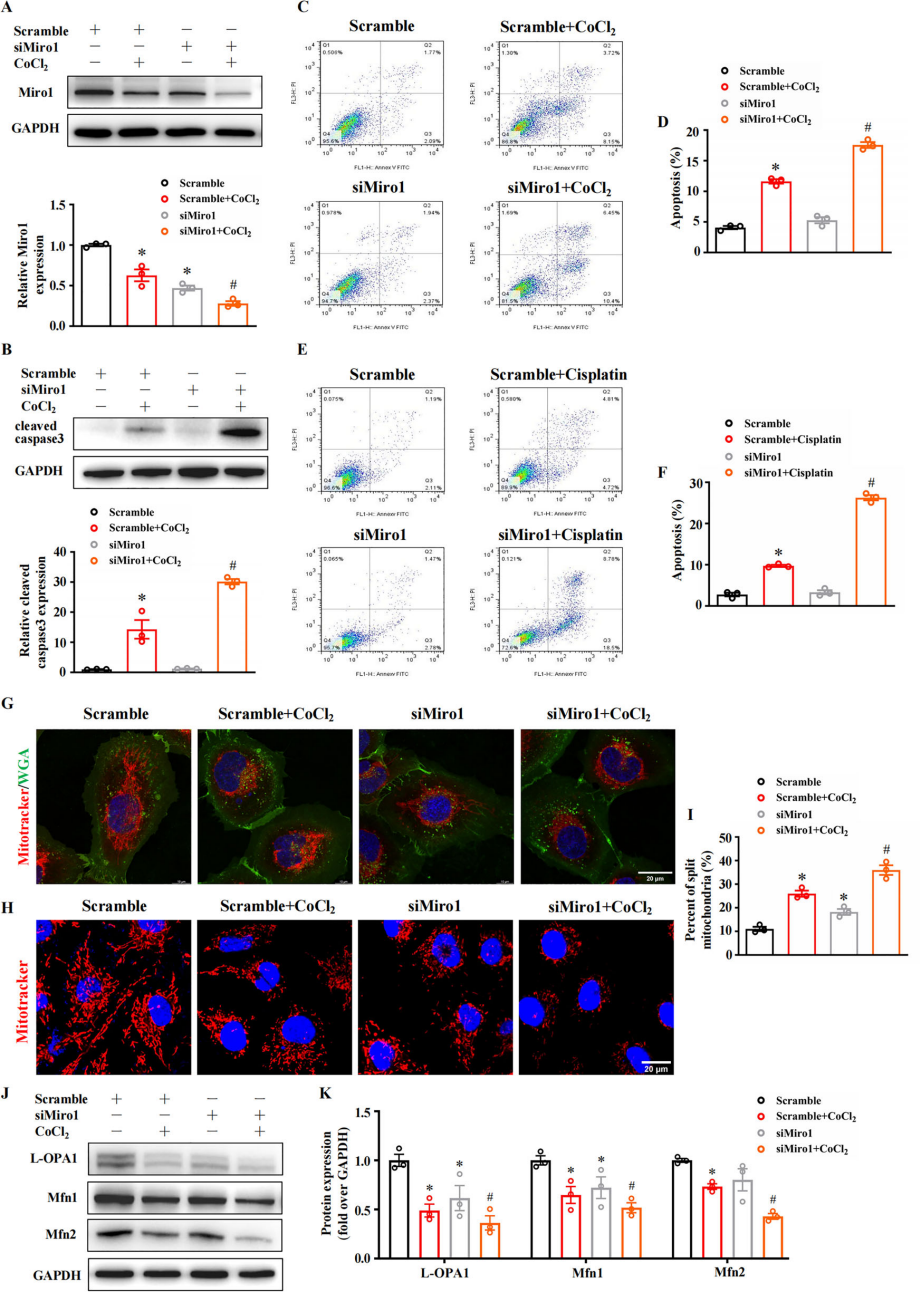
Effect of Miro1 siRNAs on HK2 cell injury and mitochondrial distribution induced by CoCl_2_ or cisplatin. (A) Western blotting and quantitation of Miro1 expression following CoCl_2_ treatment. (B) Western blotting and quantitation of cleaved caspase3 expression following CoCl_2_ treatment. (C) Representative pictures of apoptosis induced by CoCl_2_ determined by flow cytometry. (D) Quantitative analysis of CoCl_2_‐induced apoptosis. (E) Representative pictures of apoptosis induced by 25 µM cisplatin determined by flow cytometry. (F) Quantitative analysis of cisplatin‐induced apoptosis. (G) Mitochondrial distribution in HK2 cells. Live HK2 cells were co‐incubated with 10 nM MitoTracker Red, 2.5 µg/mL WGA, and 1×Hoechst 33342 diluted in HBSS buffer at 37°C for 10 min. Cells were then washed three times with HBSS and examined for mitochondrial distribution using confocal microscopy. Scale bar: 20 µm. (H) Mitochondrial morphology. Scale bar: 20 µm. (I) Quantification of mitochondrial fission. (J) Representative western blotting of L‐OPA1, Mfn1, and Mfn2. (K) Densitometry analysis of L‐OPA1, Mfn1, and Mfn2. Data are expressed as the means ± SEM (n = 3). ∗*p* < 0.05 vs. Scramble. #*p* < 0.05 vs. CoCl_2_ or cisplatin treatment group.

To study the specific roles of Miro1 for mitochondrial distribution and morphology, we labeled the HK2 cells with Mitotracker Red and WGA. As shown in Figure 2G, both Miro1 knockdown and CoCl_2_ treatments induced a significant condensation of the mitochondrial network into the juxta‐nuclear region. Morphological assessment indicated that Miro1 knockdown further increased CoCl_2_‐induced mitochondrial fission in HK2 cells (Figure 2H,I). As shown in Figure 2J,K, Miro1 knockdown promoted the reduction of proteins involved in mitochondrial fusion (L‐OPA1, Mfn1 and Mfn2). These data suggested that knockdown of Miro1 exacerbated CoCl_2_‐induced mitochondrial distribution and fission in cultured HK2 cells.

### 2.3 Knockdown of Miro1 Exacerbates Mitochondrial Dysfunction in Vitro

To examine whether this alteration could lead to a functional change due to a reorganization of the mitochondrial network in the knockdown of Miro1, we detected mitochondrial ROS (mtROS) production, oxidative phosphorylation, and biogenesis. As shown in Figure 3A,B, CoCl_2_ induced mtROS production, and the knockdown of Miro1 increased the mtROS accumulation. It was reported that damaged mitochondria generate less ATP. We found that Miro1 suppression decreased oxidative phosphorylation in HK2 cells treated with CoCl_2_ (Figure 3C,D). Consistently, Miro1 suppression exacerbated the reduction of ATP generation induced by CoCl_2_ (Figure 3E). Mic60, the core component of the mitochondrial contact site and cristae organizing system (MICOS), is an integral protein of the mitochondrial inner membrane required for maintaining mitochondrial cristae structure. Mic60 suppression compromises mitochondrial oxidative phosphorylation. Our study showed that Miro1 suppression exacerbated CoCl_2_‐induced Mic60 reduction (Figure 3F,G). Miro1 knockdown further reduced CoCl_2_‐induced downregulation of mitochondrial DNA (mtDNA) (Figure 3H). Sirt1 senses energy status and regulates mitochondrial activity. PGC‐1α promotes mitochondrial biogenesis and oxidative metabolism. Moreover, Sirt1 improves PGC‐1α expression and thus stimulates mitochondrial biogenesis. Here, we found that CoCl_2_ treatment reduced the expression of Sirt1 and PGC‐1α, and Miro1 knockdown enhanced this effect (Figure 3I,J). We also tested levels of Tom20, which is usually used as a reliable biomarker for assessing mitochondrial mass. Miro1 knockdown promoted the reduction of Tom20 levels treated by CoCl_2_. These data suggested that the knockdown of Miro1 exacerbated CoCl_2_‐induced mitochondrial dysfunction in cultured HK2 cells.

**FIGURE 3:**
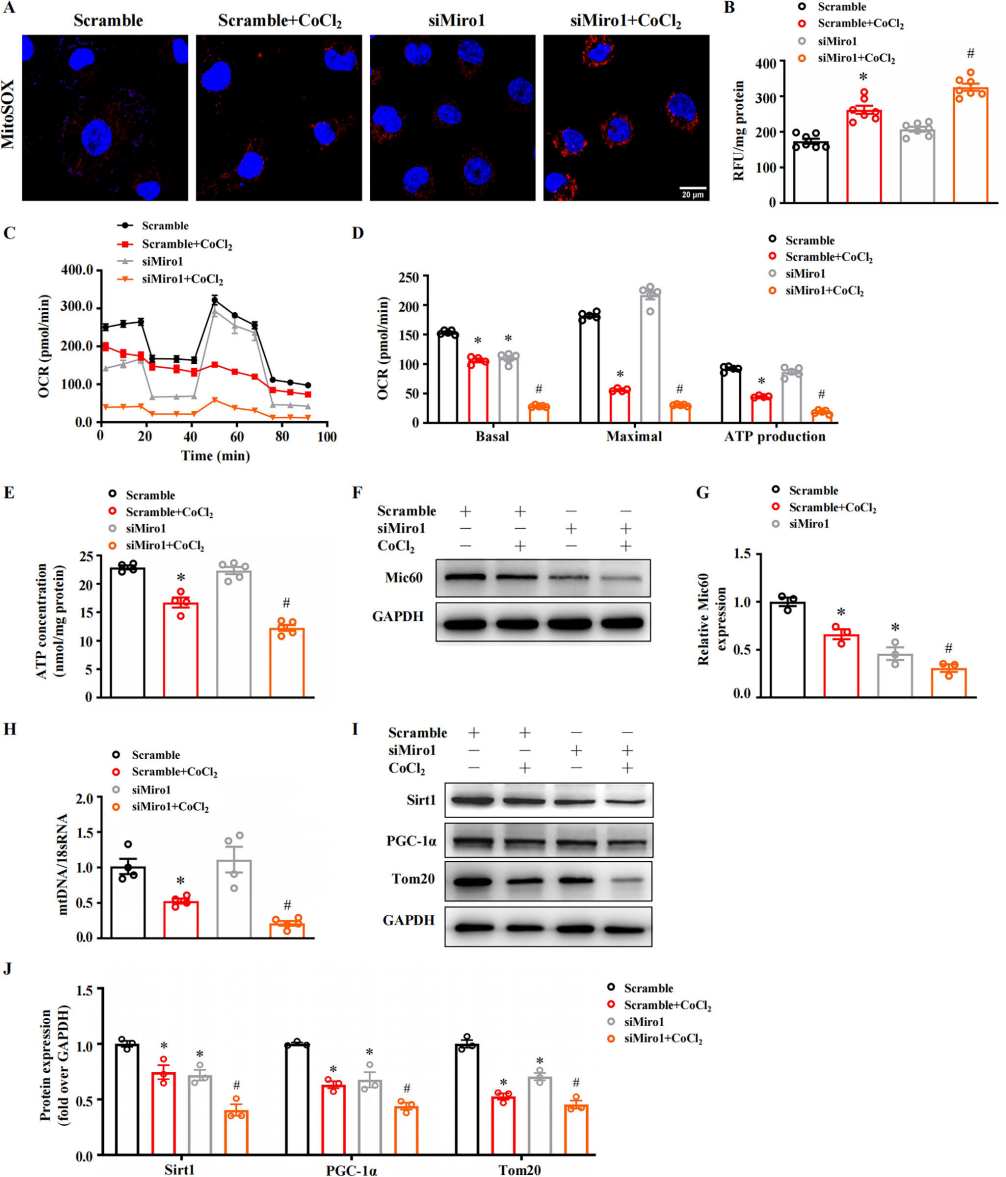
Effect of Miro1 siRNAs on mitochondrial homeostasis in HK2 cells induced by CoCl_2_. (A) MitoSOX staining of mitochondrial ROS levels. Live HK2 cells were co‐incubated with 5 µM MitoSox and 1×Hoechst 33342 diluted in HBSS buffer at 37°C for 10 min. Cells were then washed three times with HBSS and examined for mitochondrial ROS by confocal microscopy. Scale bar: 20 µm. (B) Quantification of mtROS by microplate reader. (C) Mitochondrial respirometry analysis using XF96 Extracellular Flux Analyzer after sequential injection of Oligomycin, FCCP, and Rotenone+Antimycin. (D) Oxygen consumption rate (OCR) for basal respiration, maximal respiration, and ATP production. (E) ATP contents. (F) Western blotting of Mic60. (G) Densitometry analysis of Mic60. (H) Measurement of mtDNA copy number. (I) Representative western blotting of Sirt1, PGC‐1α, and Tom20. (J) Densitometry analysis of Sirt1, PGC‐1α, and Tom20. Data are expressed as the means ± SEM (n ≥ 4 in B, D, E, and H, n = 3 in G and J). ∗*p* < 0.05 vs. Scramble. #*p* < 0.05 vs. CoCl_2_ treatment group.

### 2.4 Miro1 Overexpression Attenuates Renal Tubular Cell Injury In Vitro

To further validate the role of Miro1 in AKI, we overexpressed Miro1 in HK2 cells by using a Miro1 plasmid (Figure 4A). Western blotting results showed that Miro1 overexpression inhibited CoCl_2_‐induced cleaved caspase‐3 expression (Figure 4B). Also, Miro1 overexpression prevented both CoCl_2_ and cisplatin‐induced apoptosis in HK2 cells (Figure 4C‐F). These data indicated that Miro1 overexpression protected against acute renal tubular cell injury.

**FIGURE 4:**
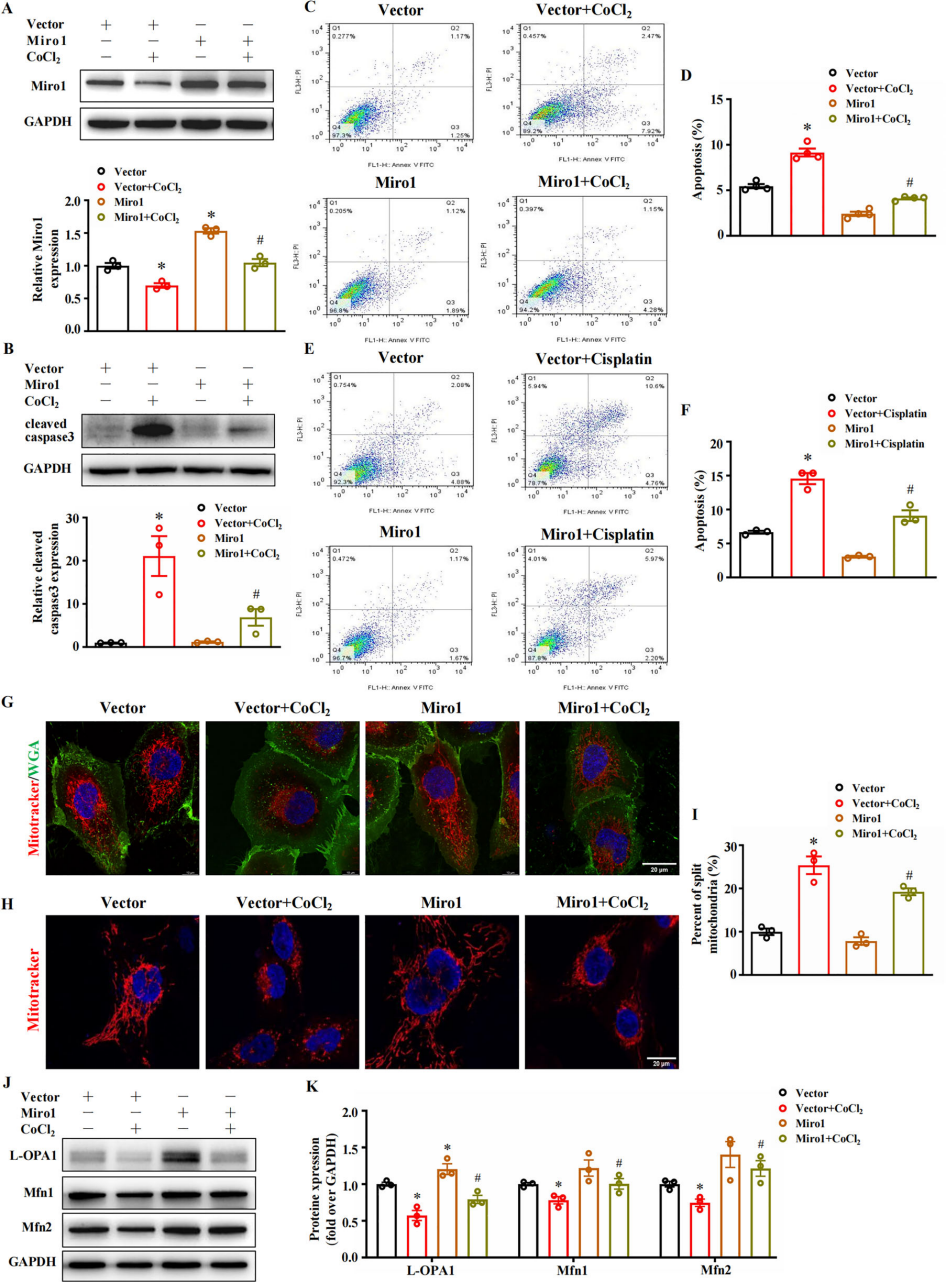
Effect of Miro1 overexpression on HK2 cell injury and mitochondrial distribution induced by CoCl_2_ or cisplatin. HK2 cells were transfected with an empty vector or a Miro1 plasmid followed by treatment with CoCl_2_ for 12 h. (A) Western blotting and quantitation of Miro1 expression following CoCl_2_ treatment. (B) Western blotting and quantitation of cleaved caspase3 expression following CoCl_2_ treatment. (C) Representative pictures of apoptosis induced by CoCl_2_ determined by flow cytometry. (D) Quantitative analysis of CoCl_2_‐induced apoptosis. (E) Representative pictures of apoptosis induced by cisplatin determined by flow cytometry. (F) Quantitative analysis of cisplatin‐induced apoptosis. (G) Mitochondrial distribution in HK2 cells stained with WGA, MitoTracker Red, and Hoechst 33342. Scale bar: 20 µm. (H) Mitochondrial morphology. Scale bar: 20 µm. (I) Quantification of mitochondrial fission. (J) Representative western blotting of L‐OPA1, Mfn1, and Mfn2. (K) Densitometry analysis of L‐OPA1, Mfn1, and Mfn2. Data are shown as means ± SEM (n = 3). ^∗^ *p* < 0.05 vs. vector. *#p* < 0.05 vs. CoCl_2_ or cisplatin treatment group.

We also confirmed the role of Miro1 in mitochondrial distribution. As shown in Figure 4G, Miro1 overexpression improved mitochondrial transport and distribution. Mitochondrial morphology revealed that Miro1 overexpression prevented CoCl_2_‐induced mitochondrial fission (Figure 4H,I). In addition, Miro1 overexpression inhibited the reduction of L‐OPA1, Mfn1, and Mfn2 in HK2 cells induced by CoCl_2_ (Figure 4J,K). These data suggested that Miro1 overexpression attenuated CoCl_2_‐induced mitochondrial distribution and fission in cultured HK2 cells.

### 2.5 Miro1 Overexpression Attenuates Mitochondrial Dysfunction In Vitro

To verify that Miro1 overexpression was associated with the improvement of mitochondrial function, we also detected mtROS production, oxidative phosphorylation, and biogenesis. Miro1 overexpression inhibited CoCl_2_‐induced mtROS production (Figure 5A,B). Miro1 overexpression reversed the reduction of mitochondrial oxidative phosphorylation (Figure 5C,D), ATP production (Figure 5E), and Mic60 protein levels (Figure 5F,G) induced by CoCl_2_. In addition, Miro1 overexpression restored CoCl_2_‐induced disruption of mitochondrial biogenesis, including mtDNA numbers (Figure 5H) and the protein levels of Sirt1, PGC‐1α, and Tom20 (Figure 5I,J). These data indicated that Miro1 overexpression protected against CoCl_2_‐induced mitochondrial dysfunction.

**FIGURE 5:**
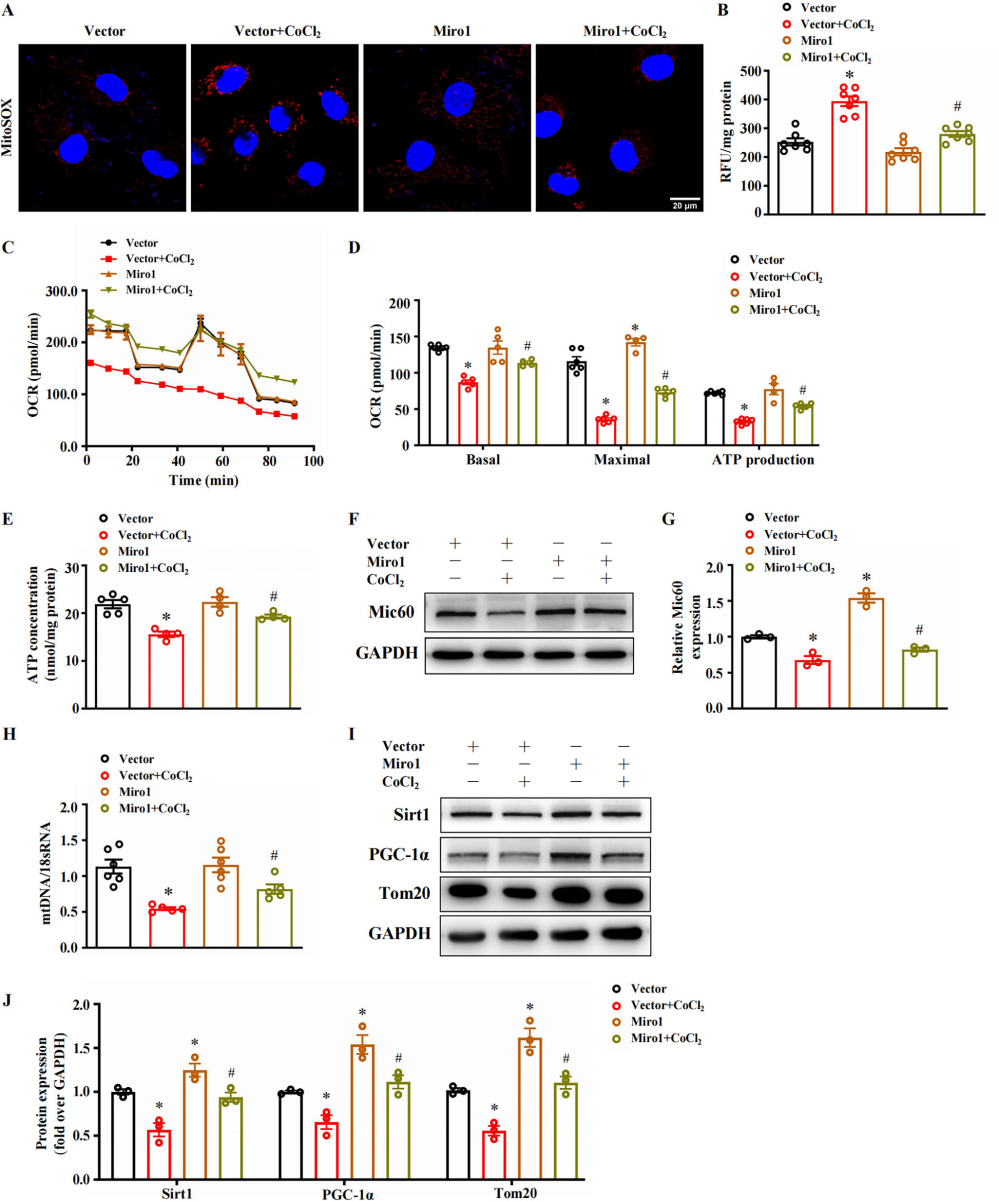
Effect of Miro1 overexpression on mitochondrial homeostasis in HK2 cells induced by CoCl_2_. (A) MitoSOX staining of mitochondrial ROS levels. Scale bar: 20 µm. (B) Quantification of mitochondrial ROS. (C) Mitochondrial respirometry analysis using XF96 Extracellular Flux Analyzer after sequential injection of Oligomycin, FCCP, and Rotenone+Antimycin. (D) OCR for basal respiration, maximal respiration, and ATP production. (E) ATP contents. (F) Western blotting of Mic60. (G) Densitometry analysis of Mic60. (H) Measurement of mtDNA copy number. (I) Representative western blotting of Sirt1, PGC‐1α, and Tom20. (J) Densitometry analysis of Sirt1, PGC‐1α, and Tom20. Data are shown as means ± SEM (n ≥ 4 in B, D, E, and H, n = 3 in G and J). ∗*p* < 0.05 vs. vector. *#p* < 0.05 vs. CoCl_2_ treatment group.

### 2.6 Overexpression of Miro1 Protects Against AKI and Alleviates Tubular Cell Mitochondrial Dysfunction In Vivo

To determine the role of Miro1 in IR‐induced AKI, we established a Miro1 conditional knock‐in (Miro1‐cKI) mouse model by CRISPR/Cas‐mediated genome engineering (Figure S2A,B). As shown in Figure S2C, the amplified Miro1 signal was completely co‐localized with AQP1 (the proximal tubule marker), which suggested that Miro1 was successfully overexpressed in the renal proximal tubular epithelial cells. Western blotting analysis further confirmed successful overexpression of Miro1 (Figure S2D,E). A flowchart for in vivo IR models was shown in Figure S2F. Periodic Acid‐Schiff (PAS) staining demonstrated that Miro1‐cKI mice were protected against tubular injury subjected to IR (Figure 6A,B). Additionally, tubular Miro1 overexpression inhibited IR‐induced elevation of Scr (Figure 6C) and BUN (Figure 6D) levels as compared to Miro1^fl/fl^ mice. Western blotting showed that Miro1 overexpression restored Miro1 protein levels and reduced cleaved caspase‐3 levels in IR models (Figure 6E,F). TUNEL assays also revealed that tubular Miro1 overexpression attenuated renal tubular cell apoptosis subjected to IR (Figure 6G,H). Moreover, we observed that the mitochondrial cristae were disrupted in Miro1^fl/fl^ subjected to IR. Miro1 overexpression sustained the structure of mitochondrial cristae (Figure 6I). In accordance with in vitro data, Miro1 overexpression reversed the IR‐mediated reduction of ATP production (Figure 6J). Thus, these results suggested that renal tubular‐specific overexpression of Miro1 could protect renal tubular cells against AKI via regulating mitochondrial function.

**FIGURE 6:**
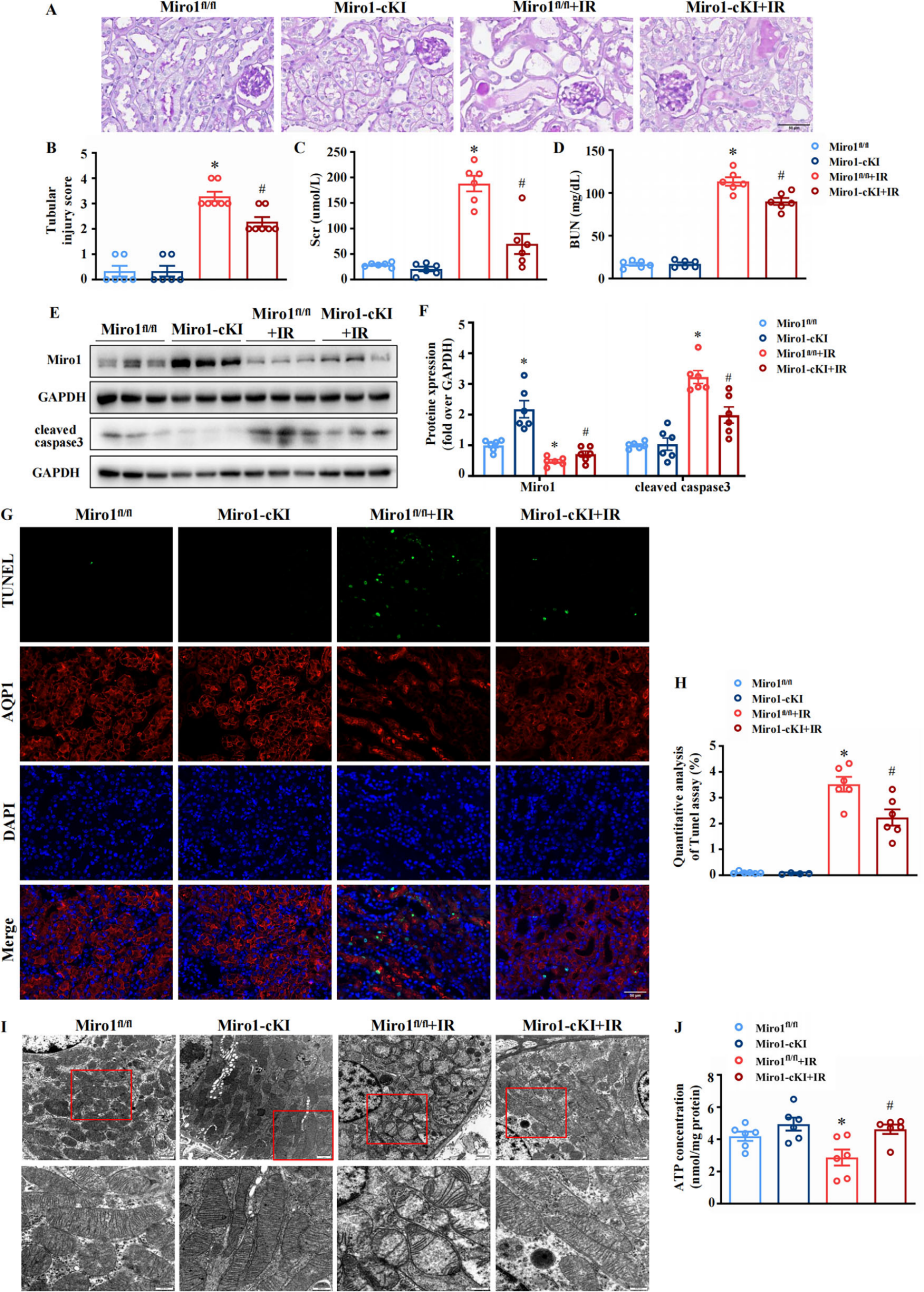
Effect of Miro1 overexpression on IR‐induced AKI in vivo. (A) PAS staining of kidneys from Miro1^fl/fl^ and Miro1‐cKI mice with or without IR. Scale bar: 50 µm. (B) Quantitation of tubular injury. The assay of Scr (C) and BUN (D). (E) Western blotting of Miro1 and cleaved caspase3 in Miro1^fl/fl^ and Miro1‐cKI mice with or without IR. (F) Densitometry analysis of Miro1 and cleaved caspase3. (G) TUNEL assay. TUNEL was co‐stained with AQP1. Scale bar: 50 µm. (H) Quantitative analysis of TUNEL assay. (I) Mitochondrial morphology from the electron microscope. (J) ATP contents. Data are shown as means ± SEM (n = 6). ∗*p* < 0.05 vs. Miro1^fl/fl^. *#p* < 0.05 vs. Miro1^fl/fl^+IR treatment group.

### 2.7 Sirt6 Interacts With and Stabilizes Miro1

Sirtuins are a family of NAD^+^‐dependent histone deacetylases, which are involved in inflammation, energy metabolism, stress resistance, and cancer. Previous studies reported that Sirt1, 6, and 7 played key roles in AKI. To identify the proteins that interact with Miro1, LC/MS/MS analysis of peptides generated by digestion of the immunoprecipitated samples showed that Sirt6 might be the candidate protein (Figure S3A). Notably, while HDAC6 has been reported to interact with Miro1 in neuronal cells [18], our Shotgun Proteomics and analysis of protein‐protein interaction (PPI) network in renal tubular cells identified Sirt6, but not HDAC6, as a potential Miro1‐binding partner, indicating a cell‐type‐specific interactome (Figure S3B). To gain further insight into the potential involvement of sirtuin family members in kidney injury, we interrogated the human KPMP database for their expression patterns across different disease states. As depicted in Figure S3C, the transcript levels of most sirtuin members (Sirt1–Sirt7 except Sirt2) were predominantly elevated in injured tubules relative to normal tubules. Notably, although Sirt6 was not the most abundantly expressed member within the sirtuin family, it emerged as the only candidate identified from our mass spectrometry screening. This finding prompted us to select Sirt6 for further mechanistic exploration.

It was demonstrated that Sirt6 relieved AKI caused by sepsis and cisplatin [17]. To further investigate the effect of Sirt6 on Miro1, we first verified the interaction between these two proteins. GST pull‐down was used to examine whether there is a direct interaction between them. As shown in Figure 7A, GST‐Miro1, but not GST alone, pulled down Sirt6, indicating a specific direct interaction between Miro1 and Sirt6. Co‐immunoprecipitation (co‐IP) experiments were also performed. As expected, Sirt6/Miro1 interaction was detectable through endogenous co‐IP assays in HK2 cells (Figure 7B). Furthermore, the exogenous interaction between these two proteins was confirmed in HEK293T cells transfected with Flag‐tagged Sirt6 and Myc‐tagged Miro1 (Figure 7C). We also verified the interaction between Miro1 and Sirt6 in kidney tissue homogenate of normal mice. As shown in Figure S4A,B, immunoprecipitation with an anti‐Miro1 antibody followed by western blotting detected Sirt6, and the reciprocal IP with an anti‐Sirt6 antibody detected Miro1, indicating that these two proteins interact with each other in vivo. As shown by the immunofluorescence staining, Sirt6 was mainly localized in the nucleus under normal conditions but partially translocated to the cytoplasm and colocalized with Miro1 upon CoCl_2_ treatment (Figure 7D).

**FIGURE 7:**
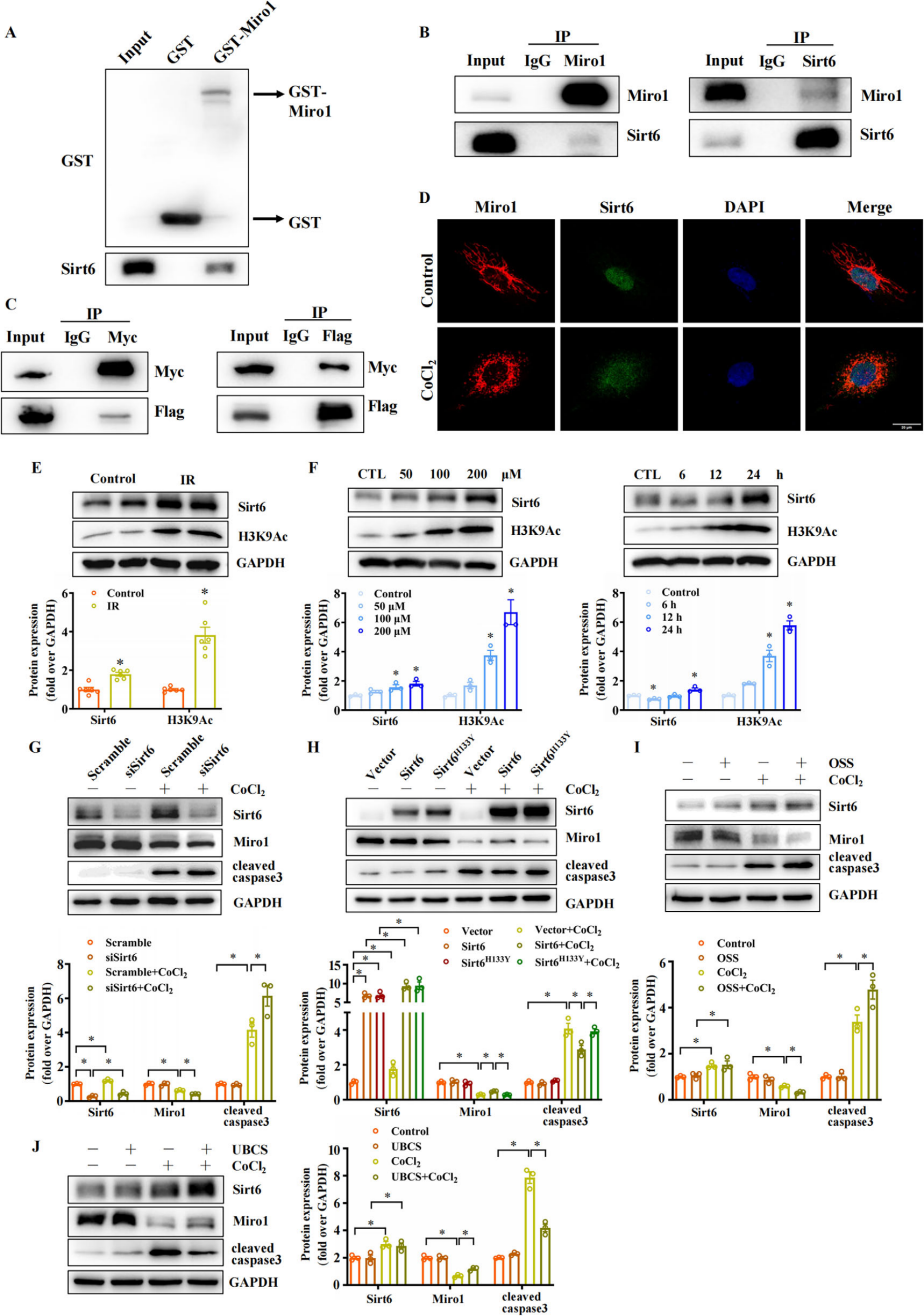
The interaction of Miro1 and Sirt6. (A) GST pull‐down analysis of the interaction between GST‐Miro1 and Sirt6. Immobilized GST or GST‐Miro1 on glutathione‐sepharose beads was incubated with cell lysates expressing Sirt6. Bound proteins were detected by immunoblotting with anti‐Sirt6 antibody. (B) HK2 cells were lysed and subjected to co‐IP with anti‐Miro1 antibody (Left) and anti‐Sirt6 antibody (Right), respectively. IgG was used as a negative control. Immunoprecipitated Miro1 and Sirt6 were detected by western blotting. (C) HEK293T cells were transfected with Myc‐Miro1 and Flag‐Sirt6 plasmids. Cell lysates were immunoprecipitated with anti‐Myc antibody (Left) and anti‐Flag antibody (Right), respectively. IgG was used as a negative control. Immunoprecipitated Miro1 and Sirt6 were detected by western blotting. (D) Immunofluorescence staining was performed using antibodies against Miro1 (red) and Sirt6 (green). DAPI (blue) was used to stain the nuclei. Scale bars: 20 µm. (E) Sirt6 protein levels and the acetylation level of the classic substrate of Sirt6, H3K9, were detected by Western blotting in renal tissue of IR model mice. (F) Sirt6 protein level and H3K9 acetylation level were detected by Western blotting in HK2 cells after treatment with different concentrations of CoCl_2_ (Left) or stimulation with different reoxygenation times following 12 h treatment with CoCl_2_ (Right). (G–J) Protein levels of Sirt6, Miro1, and cleaved caspase3 were detected by Western blotting in CoCl_2_‐treated HK2 cells after different treatments: (G) Pre‐transfected with Scramble or siSirt6, (H) Pre‐transfected with Vector, Sirt6 or Sirt6^H133Y^ plasmids, (I) Treatment with Sirt6 inhibitor OSS, (J) Treatment with Sirt6 activator UBCS. Data are shown as means ± SEM (n = 6 in E and n = 3 in F‐J). ^∗^ *p* < 0.05 vs. Control (E,F) or as indicated (G–J).

We next examined the expression level of Sirt6 and its deacetylase activity in renal cortex tissue of the IR mouse model, as indicated by the acetylation level of its specific substrate H3K9. As shown in Figure 7E, although the expression level of Sirt6 was significantly upregulated in IR mice, its deacetylase activity was decreased, indicating that the increased protein level might be a negative feedback regulation. In HK2 cells treated with various doses of CoCl_2_ for 12 h and reperfusion for 24 h (Left of Figure 7F), the protein level of Sirt6 was upregulated as the concentration of CoCl_2_ increased. In HK2 cells treated with 200 µM CoCl_2_ for 12 h and reperfusion for various times (Right of Figure 7F), Sirt6 decreased at 6 h and then increased till 24 h. In the concentration and time gradient experiments, the acetylation of H3K9 both increased, showing a decreasing trend in Sirt6 deacetylase activity after CoCl_2_ treatment. To further validate the clinical relevance of Sirt6, we performed immunohistochemical staining on renal biopsy specimens obtained from patients with ATN. Compared with the MCD control group, Sirt6 expression levels were significantly elevated in renal tubular cells of ATN patients (Figure S3D,E). Furthermore, Sirt6 expression levels were positively correlated with both Scr (r = 0.618, *p* < 0.01) and BUN (r = 0.574, *p* < 0.01), suggesting a potential association with renal function impairment (Figure S3F,G). We also examined the temporal dynamics of Sirt6 expression and enzymatic activity in a murine model of renal IR injury. As shown in Figure S3H,I, although without statistical significance, there was a downward trend in Sirt6 protein levels at 8 h post‐reperfusion, followed by a substantial upregulation on Day 1 and Day 3 post‐reperfusion. Intriguingly, the acetylation level of H3K9, a well‐established substrate of Sirt6, progressively increased over the course of IR, implying a gradual decline in Sirt6 deacetylase activity despite its elevated protein expression. This inverse correlation between Sirt6 expression and activity was consistent with our observations in vitro.

We further investigated the effect of Sirt6 on Miro1 protein expression level and cell apoptosis in HK2 cells treated with CoCl_2_. Knockdown of Sirt6 further aggravated the downregulation of Miro1 protein level and upregulation of cleaved caspase3 after treatment with CoCl_2_ (Figure 7G), while overexpression of Sirt6 alleviated the CoCl_2_‐induced Miro1 downregulation and cell apoptosis (Figure 7H). However, overexpression of Sirt6^H133Y^, a mutation in the active site of the deacetylase, did not show these improving effects, indicating that the protective effects of Sirt6 described above might depend on its deacetylase activity. Moreover, usage of the Sirt6‐specific inhibitor OSS‐128167 (abbreviated as OSS throughout) and the agonist UBCS039 (abbreviated as UBCS throughout) verified the effect of deacetylase activity of Sirt6 on Miro1 protein level and apoptosis in CoCl_2_‐treated HK2 cells. Based on substrate acetylation levels and CCK‐8 assays, the effective concentrations of OSS and UBCS were determined as 200 µM and 60 µM, respectively (Figure S4C–F). It was shown in Figure 7I that inhibition of Sirt6 deacetylase activity by OSS exacerbated the reduction of Miro1 and cell apoptosis induced by chemical hypoxia and reoxygenation, while the activation of Sirt6 by UBCS evidently relieved cell injury (Figure 7J). Taken together, Sirt6 interacted with Miro1. CoCl_2_ treatment caused an increase  of Sirt6 protein levels and a reduction of its deacetylase activity. Knockdown or inhibition of Sirt6 induced decreased Miro1 protein levels and increased apoptosis in CoCl_2_‐treated HK2 cells. Conversely, overexpression or activation of Sirt6 suppressed CoCl_2_‐induced Miro1 reduction and cell apoptosis.

### 2.8 Sirt6 Deacetylates Miro1 and Protects It From the Proteasomal Degradation Pathway

We further investigated the mechanism by which Sirt6 stabilizes Miro1 levels. In the early chemical hypoxia phase simulated by CoCl_2_, Sirt6 and Miro1 levels decreased, accompanied by increased Miro1 acetylation (Figure 8A). When treating HK2 cells with TSA (a deacetylase inhibitor of the HDAC family) and NAM (a deacetylase inhibitor of the sirtuin family), we observed a significant upregulation of Miro1 acetylation following NAM treatment (Figure 8B), indicating that the sirtuin family primarily regulates deacetylation of Miro1. Acetylation of H3K9 was used to demonstrate reagent accuracy. Subsequently, overexpression of Sirt6 or the Sirt6^H133Y^ mutant (with deacetylase functional domain mutations) in HK2 cells revealed that Sirt6 overexpression significantly decreased Miro1 acetylation level (Figure 8C), whereas Sirt6^H133Y^ overexpression showed no effect. This demonstrates that Miro1 acetylation is regulated by Sirt6 and correlates with its deacetylase activity.

**FIGURE 8:**
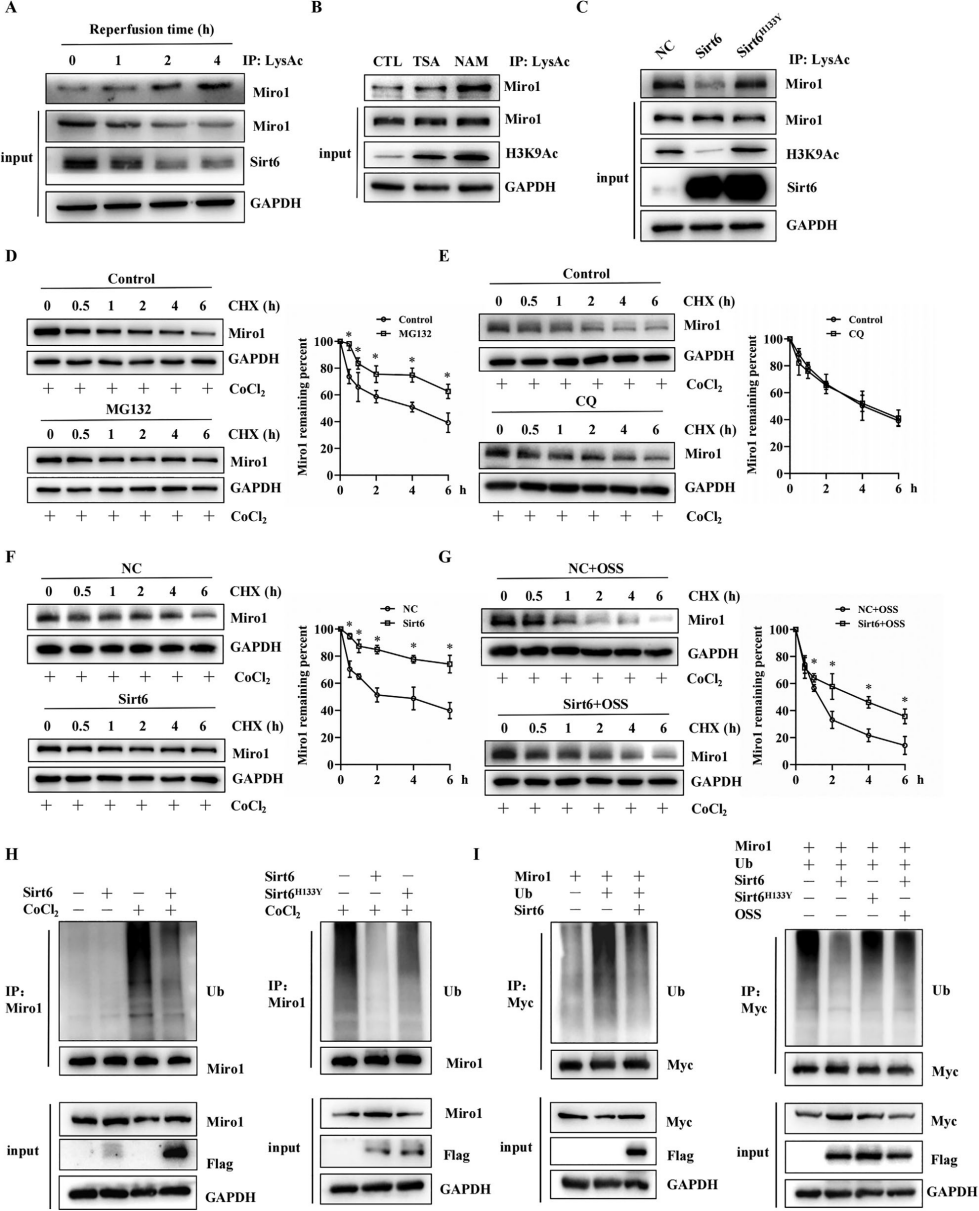
Sirt6 deacetylation inhibited ubiquitination degradation to promote Miro1 protein stability. (A) After CoCl_2_ treatment for different times, HK2 cell homogenates were immunoprecipitated with anti‐acetylated lysine antibody. Western blotting showed Miro1 acetylation was increased after CoCl_2_ treatment. GAPDH was used as a loading control. (B) Immunoprecipitation detection of the acetylation of Miro1 after treatment with TSA (250 µg/µL) and NAM (10 mM) for 24 h, respectively. (C) Immunoprecipitation detection of the acetylation of Miro1 after transfection with Sirt6 or Sirt6^H133Y^ plasmids in HK2 cells. (D‐G) HK2 cells were exposed to serum‐free medium with CoCl_2_ for 12 h to induce hypoxia, followed by reoxygenation with normal complete medium. CHX (0.1 mM) was then added at the indicated time points in a reverse time‐course manner, and cells were collected at 6 h post‐reoxygenation. Western blotting analysis of Miro1 protein stability was performed after (D) 10 µM MG132 treatment, (E) 20 µM CQ treatment, (F) transfection with Sirt6 plasmid, and (G) transfection with Sirt6 plasmid and treatment with Sirt6 inhibitor OSS. (H) Left: HK2 cells were transfected with control and Flag‐Sirt6 plasmids and treated with or without CoCl_2_ before harvest. Right: HK2 cells were transfected with control, Flag‐Sirt6, or Flag‐Sirt6^H133Y^ plasmids and treated with CoCl_2_ before harvest. Cell lysates were immunoprecipitated with anti‐Miro1 antibody and immunoblotted with anti‐ubiquitin (Ub) antibody. (I) Left: HEK293T cells were transfected with the Myc‐Miro1 plasmid, together with the HA‐Ub plasmid, or with both the HA‐Ub and Flag‐Sirt6 plasmids. Right: HEK293T cells were transfected with Myc‐Miro1 and HA‐Ub plasmids, together with Flag‐Sirt6, or Flag‐Sirt6^H133Y^ plasmids and treated with OSS before harvest. The anti‐Myc immunoprecipitation was followed by Western blotting with anti‐Ub antibody and anti‐Myc antibody. Data are shown as means ± SEM (n = 3). ∗*p* < 0.05 vs. Control.

Now that the acetylation level of Miro1 affects its stability, we further investigated through which pathway Sirt6 affects the degradation of Miro1. MG132, a classic proteasome inhibitor, dramatically enhanced the protein stability of Miro1 protein in HK2 cells after treatment with CoCl_2_ and the protein synthesis inhibitor cycloheximide (CHX) (Figure 8D). However, Miro1 protein stability did not show significant change under chloroquine (CQ, an autophagy inhibitor) treatment (Figure 8E). Overexpression of Sirt6 evidently prolonged the stability of Miro1 (Figure 8F). In addition, treatment with Sirt6 inhibitor OSS in cells overexpressing Sirt6 counteracted the effect of Sirt6 on Miro1 stability, and the use of OSS accelerated the degradation of Miro1 (Figure 8G). These data indicated that stabilization effect of Sirt6 on Miro1 was probably mediated by the proteasome but not the lysosome.

Proteasome protein degradation is usually associated with the ubiquitination of target proteins. We then examined the effect of Sirt6 on the ubiquitination level of Miro1. As shown on the left of Figure 8H, the ubiquitination level of Miro1 was upregulated under CoCl_2_ treatment in HK2 cells. In contrast, transfection of the Sirt6 plasmid resulted in a decrease in Miro1 ubiquitination. However, transfection of the mutant Sirt6^H133Y^plasmid had almost no effect on CoCl_2_‐induced Miro1 ubiquitination (Right of Figure 8H). Similar results were observed in HEK293T cells transfected with Myc‐Miro1 alone or together with HA‐Ub and Flag‐Sirt6 (Left of Figure 8I). Moreover, it was found that in HEK293T cells transfected with Miro1 and Ub plasmids, the ubiquitination level of Miro1 could not be improved by simultaneous transfection of a mutant Sirt6 plasmid or Sirt6 inhibitor OSS together with transfection of the Sirt6 plasmid (Right of Figure 8I). The above experiments demonstrated that the effect of Sirt6 on Miro1 ubiquitination was mainly mediated by its deacetylase activity.

### 2.9 Miro1 Acts Downstream of Sirt6 to Preserve Mitochondrial Homeostasis

To identify the acetylation sites of Miro1, we performed mass spectrometry on CoCl_2_‐treated HK2 cells, which identified K182 as a potential acetylation site (Figure S5). Based on previous reports of K92 and K512 as acetylation sites of Miro1 in humans [19], we generated three K→R mutants. In these mutants, the substitution of a lysine with an arginine mimics constitutive deacetylation. Validation experiments in HK2 cells (Figure 9A) showed that mutations at K92 and K182, but not K512, significantly diminished Miro1 acetylation, indicating that K92 and K182 are the major acetylation sites of Miro1 under AKI conditions. We next proceeded to determine which site serves as the direct target of Sirt6‐mediated deacetylation. As shown in Figure 9B, upon co‐transfection of Sirt6 with the K92R mutant, the acetylation level of Miro1 was further decreased, suggesting that K92 is not the target site for Sirt6‐mediated deacetylation. However, co‐transfection of Sirt6 with the K182R mutant failed to cause any additional reduction in Miro1 acetylation (Figure 9C), demonstrating that K182 is the specific residue through which Sirt6 exerts its deacetylating activity on Miro1.

**FIGURE 9:**
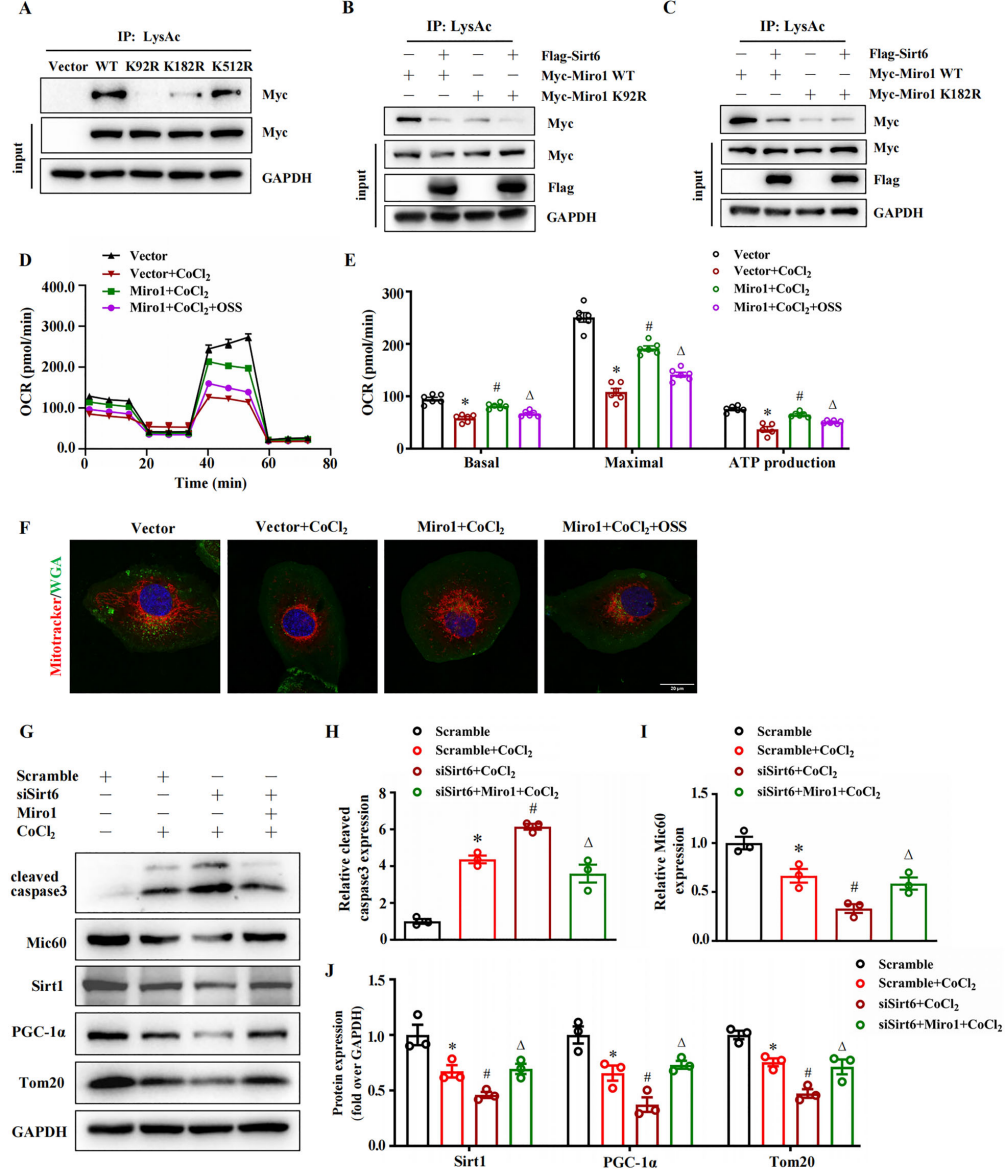
Sirt6‐mediated deacetylation of Miro1 regulates mitochondrial respiration, distribution, and homeostasis. (A) Validation of acetylation‐deficient mutants. Wild‐type (WT) and indicated K→R mutants were expressed in HK2 cells. Acetylated proteins were immunoprecipitated (IP) with an anti‐acetylated‐Lysine antibody and detected by western blotting with an anti‐Myc tag antibody. (B,C) Effects of Sirt6 on Miro1 acetylation. Flag‐Sirt6 was co‐transfected into HK2 cells with the Myc‐Miro1 wild‐type (WT) or K92R (B) or K182R (C) constructs with treatment of 200 µM CoCl_2_ for 12 h and reperfusion for 4 h. Immunoprecipitation was performed using anti‐acetylated‐Lysine antibody, and the acetylation level of Miro1 was measured by western blotting. (D) Mitochondrial respirometry analysis using XF96 Extracellular Flux Analyzer after sequential injection of Oligomycin, FCCP, and Rotenone+Antimycin. (E) OCR for basal respiration, maximal respiration, and ATP production. Data were shown as means ± SEM (n = 6). ∗*p* < 0.05 vs. Vector, #*p* < 0.05 vs. Vector+CoCl_2_ treatment group, Δ*p* < 0.05 vs. Miro1+CoCl_2_ treatment group. (F) Mitochondrial distribution in HK2 cells stained with WGA, MitoTracker Red and Hoechst 33342. Scale bar: 20 µm. (G) Representative western blotting of cleaved caspase3, Mic60, Sirt1, PGC‐1α and Tom20. (H) Densitometry analysis of cleaved caspase3. (I) Densitometry analysis of Mic60. (J) Densitometry analysis of Sirt1, PGC‐1α and Tom20. Data are expressed as the means ± SEM (n = 3). ∗*p* < 0.05 vs. Scramble, #*p* < 0.05 vs. Scramble + CoCl_2_ treatment group, Δ*p* < 0.05 vs. siSirt6+CoCl_2_ treatment group.

To determine whether Miro1 functions as a critical downstream effector of Sirt6, we performed both loss‐ and gain‐of‐function rescue experiments. First, we overexpressed Miro1 in cells and concurrently inhibited Sirt6 by OSS. Notably, Sirt6 suppression partially abolished the beneficial effects of Miro1 overexpression on mitochondrial oxidative phosphorylation (Figure 9D,E) and mitochondrial distribution (Figure 9F), indicating that Sirt6 activity is indispensable for the optimal action of Miro1. In a complementary approach, we observed that Miro1 overexpression after Sirt6 knockdown rescued the exacerbated mitochondrial injury induced by CoCl_2_ treatment. As shown in Figure 9G, compared with scramble controls, Sirt6‐knockdown cells exhibited exacerbated apoptosis (Figure 9H) and decreased expression of mitochondrial inner membrane protein Mic60 (Figure 9I), as well as a marked further decline in the levels of mitochondrial regulators (Sirt1, PGC‐1α, Tom20, Figure 9J) upon CoCl_2_ treatment. However, ectopic Miro1 overexpression in this setting partially ameliorated the cellular damage and mitochondrial impairment. These reciprocal rescue experiments firmly establish that Miro1 serves as a principal executor downstream of Sirt6, and their coordinated action is essential for counteracting mitochondrial impairment.

## 3 Discussion

Here, we characterized the critical role of a novel Sirt6‐Miro1 pathway in AKI. Miro1 was downregulated in acute renal tubular cell injury. Miro1 provided a protective effect in AKI both in vitro and in vivo. At the molecular level, Miro1 exerted a reno‐protective effect by regulating mitochondrial homeostasis, including the modulation of mitochondrial distribution and dynamics, the inhibition of mitochondrial ROS production, and the maintenance of oxidative phosphorylation and biogenesis. Mechanistically, Sirt6 interacted with Miro1 and stabilized its expression via deacetylation. Under CoCl_2_ treatment, the combination of these two proteins reduced the acetylation of Miro1, leading to an increase in Miro1 degradation, ultimately causing mitochondrial dysfunction and tubular cell damage (Figure 10).

**FIGURE 10:**
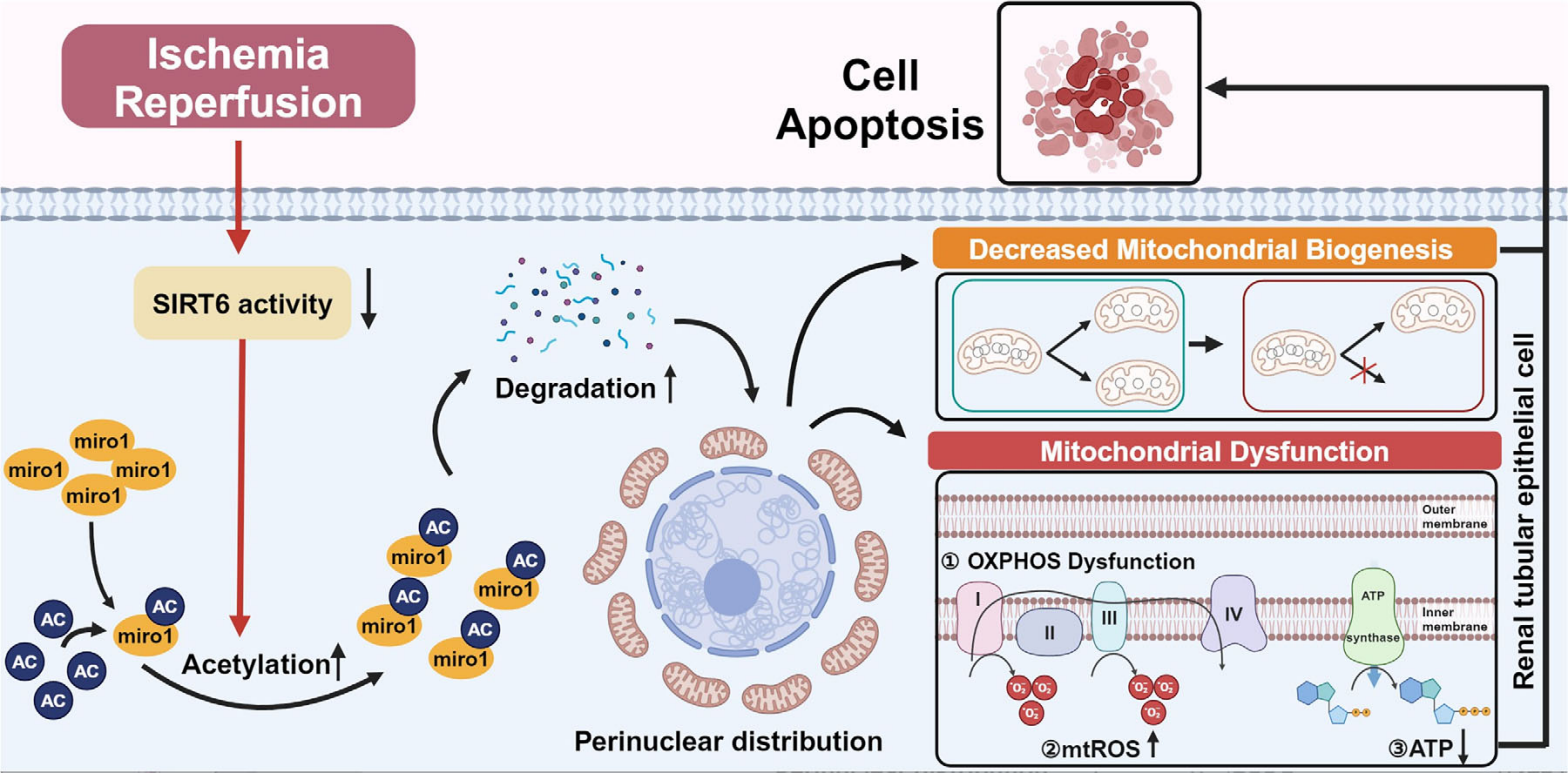
Schematic illustration of the mechanism of Miro1 in AKI. In the process of AKI, the decrease in deacetylase Sirt6 activity leads to an increase in Miro1 acetylation and a decrease in Miro1 expression, resulting in mitochondrial distribution disorders, ultimately leading to mitochondrial homeostasis imbalance and renal tubular cell damage.

Defects in mitochondrial homeostasis are a key part of the etiology of AKI [4]. Mitochondrial dynamics, mitochondrial biogenesis, and mitophagy are the key steps in mitochondrial quality surveillance. They are coordinated to maintain mitochondrial homeostasis and restore damaged mitochondrial function [20]. Multiple mitochondrial proteins play critical roles in AKI. Our previous study showed that mitochondrial fission protein dynamin‐related protein 1 (Drp1) promoted cisplatin‐induced acute renal tubular epithelial cell injury via stimulating mitochondrial fragmentation [21]. Modulating mitochondrial fission provided a protective effect in IR‐induced AKI [22]. Moreover, Pink1‐Parkin mediated mitophagy attenuated cisplatin nephrotoxicity by improving mitochondrial function [23]. Miro1 is a mitochondrial Rho GTPase on the outer mitochondrial membrane (OMM) that maintains proper mitochondrial distribution and structure, which is essential for normal mitochondrial function and cellular health [24]. Our current study found that the expression of Miro1 was decreased in renal tubular cells of ATN patients and AKI mice induced by IR and cisplatin. Miro1 protected against acute renal tubular cell injury induced by CoCl_2_ and cisplatin. Using a conditional Miro1 knock‐in mouse, we confirmed the protective effects of Miro1 in IR‐induced AKI. Similarly, Miro1‐mediated mitochondrial transport pathway exerted a neuroprotective role in traumatic brain injury [25]. It has been proven that Miro1 was related to the occurrence and development of various neurological disorders [26, 27]. The role of Miro1 in other kidney diseases deserves further in‐depth investigations.

Mitochondrial distribution and dynamics are indispensable components in shaping the mitochondrial network [28]. The interplay between motility, fusion, and fission works to coordinate the dynamic distribution of mitochondria within a cell. In accordance with the function of Miro1 for mitochondrial transport, our data showed that the inhibition of Miro1 or the treatment of CoCl_2_ also affected the positioning of mitochondria, with mitochondria becoming more perinuclear in distribution, and Miro1 overexpression could reverse this effect. Several studies have shown that overexpressing Miro1 increased mitochondrial length in neurons [29]. Consistently, our results demonstrated that Miro1 protected against CoCl_2_‐induced mitochondrial fission. Mitochondrial dynamics are primarily regulated by fusion and fission. Mitochondrial fission is conducted by Drp1 [30]. A recent finding indicated that Ca^2+^ signals could induce a rapid mitochondrial morphological change via a Miro1‐dependent way rather than a Drp1‐dependent way [31]. In the present study, we found Miro1 regulated mitochondrial dynamics through modulating the expression of OPA1, Mfn1 and Mfn2. Further research is needed on how Miro1‐mediated mitochondrial movement regulates mitochondrial morphology. Moreover, it has been reported that peroxisomally localized Miro1 can modulate long‐range trafficking and the subsequent redistribution of peroxisomes [32]. Peroxisomal dysfunction has been linked to severe metabolic disorders [33]. A previous study revealed that peroxisomal fatty acid oxidation might be a common feature in the progression of AKI to CKD [34]. The role of Miro1‐mediated peroxisome movement in AKI is still unknown, which needs further study in the future.

Excessive mitochondrial fission results in elevated ROS [30]. Mitochondrial ROS promotes mitochondrial dysfunction, increases tubular cell injury, and promotes AKI [35]. Our research confirmed that Miro1 could inhibit the generation of mtROS in AKI. Furthermore, the MICOS complex acts as a bridge between the OMM and the inner mitochondrial membrane (IMM). A previous study reported that endogenous human Miro1 binds to Mic60 in HEK293T cells [36]. Loss of Miro1 is associated with altered cristae morphology [37]. Mic60, a central subunit of the MICOS complex, is critical for efficient oxidative phosphorylation [38]. Our data also demonstrated that Miro1 could restore the expression of Mic60 and promote mitochondrial oxidative phosphorylation and energy generation. In addition, we showed that Miro1 could regulate mitochondrial biogenesis, as indicated by increased levels of mtDNA numbers, Sirt1, PGC‐1α, and Tom20. Therefore, we verified that Miro1 was a central point at the OMM to coordinate mitochondrial homeostasis. It was reported that the inhibition of Mic60 compromised mitochondrial transcription and oxidative phosphorylation [39]. However, the exact mechanism by which Miro1 regulates mitochondrial homeostasis needs further exploration.

Post‐transcriptional modifications of RNA play a major role in disease pathogenesis [40]. We found the interaction of Miro1 with Sirt6. Sirt6 is an NAD^+^ dependent deacetylase that belongs to the mammalian sirtuin family, which provides a protective role in AKI [41]. Of note, Miro1 has recently been identified as a deacetylation substrate of HDAC6 in neuronal cells, where HDAC6‐mediated Miro1 deacetylation at K105 impairs mitochondrial transport and inhibits axonal regeneration following central nervous system injury [19]. In our study, Shotgun Proteomics screening in renal tubular cells identified Sirt6, but notably not HDAC6, as a specific Miro1‐interacting partner under AKI‐related stress, indicating a cell type‐specific interactome that distinguishes the kidney from the neuronal context. We found that CoCl_2_ treatment increased the levels of acetylated Miro1 along with decreased protein levels of Miro1. Therefore, the protective effect of Miro1 in AKI might depend on the binding and the deacetylation modification of Sirt6. Sirt6 is mainly located in the nucleus. It was demonstrated that cytoplasmic proteins could be deacetylated by the cytoplasmic accumulation of Sirt6 [42]. Sirt6 could deacetylate nuclear and cytoplasmic proteins [43]. Through co‐IP and immunofluorescence assay, we demonstrated that Sirt6 and Miro1 interacted with each other. Overexpression or activation of Sirt6 significantly inhibited the decrease in Miro1 protein level and cell apoptosis induced by chemical hypoxia stimulation. Further experiments on protein stability and ubiquitination levels suggested that Sirt6 might inhibit degradation of Miro1 through the proteasomal pathway, in which its deacetylase activity is crucial. However, how to drive Sirt6 to aggregate from the nucleus to the cytoplasm and bind to Miro1 in AKI is currently an unsolved problem. A previous study found that Sirt6‐mediated deacetylation of PGC‐1α could also prevent mitochondrial dysfunction and tubular cell apoptosis in AKI [42]. We propose that Sirt6 might regulate PGC‐1α and Miro1 in a temporally and spatially coordinated manner. Under basal conditions, nuclear Sirt6 maintains PGC‐1α‐mediated mitochondrial biogenesis. Upon AKI stress, a pool of Sirt6 translocates to the cytoplasm to directly deacetylate Miro1 and preserve mitochondrial trafficking, a stress‐inducible rapid response that complements the slower transcriptional regulation via PGC‐1α. Whether these two pathways operate sequentially or in parallel, and how their relative contributions vary with injury severity and phase, warrant further investigation. Importantly, it is noteworthy that while Sirt6 protein abundance was elevated in AKI kidneys, its deacetylase activity was diminished, consistent with the NAD^+^ depletion characteristic of AKI [44]. This protein‐activity dissociation indicates that Sirt6 function is controlled by its enzymatic activity rather than its protein level. An increase in Sirt6 protein levels may be a form of compensation, but ultimately, it is insufficient to offset the damage.

Sirt6 nucleocytoplasmic shuttling has emerged as a key regulatory mechanism in various pathological contexts. Under hyperglycemic stress, Sirt6 translocates from the nucleus to the cytoplasm via Importin 13 in vascular smooth muscle cells, where it deacetylates ENO3 to drive glycolytic reprogramming [45]. This provides a mechanistic precedent for stress‐induced Sirt6 cytoplasmic translocation, supporting our observation that Sirt6 accumulates in the cytoplasm upon CoCl_2_ treatment and interacts with the mitochondrial outer membrane protein Miro1. Regarding Sirt6 in AKI, several recent studies have expanded our understanding of its renoprotective functions. Sirt6 mitigates AKI by enhancing lipid metabolism and reducing tubular epithelial cell apoptosis via suppression of the ACMSD signaling pathway [46]. Additionally, Sirt6 promotes macrophage polarization from M1 to M2 subtype during the AKI‐to‐CKD transition, highlighting its immunomodulatory role in renal injury and repair [47]. Together with our finding that Sirt6 directly deacetylates Miro1 to stabilize mitochondrial trafficking machinery, these studies establish Sirt6 as a multifaceted renoprotective molecule acting through distinct mechanisms: transcriptional regulation of metabolism, immunomodulation, and direct post‐translational regulation of mitochondrial proteins.

Our finding that Sirt6‐mediated deacetylation stabilizes Miro1 adds to a growing list of regulatory mechanisms targeting Miro1. Notably, armadillo repeat‐containing X‐linked protein 1 (Armcx1) has been shown to interact with Miro1 and promote mitochondrial transport, exerting neuroprotective effects in traumatic brain injury [48]. By using live‐cell time‐lapse imaging, that study provided direct evidence that Armcx1 enhances mitochondrial movement and distribution through its interaction with Miro1 [25]. Armcx1 directly associates with Miro1 to facilitate mitochondrial recruitment to the trafficking machinery and promote anterograde transport, whereas our current study suggests Sirt6 regulates Miro1 at the post‐translational level by deacetylating it at K182, thereby inhibiting its ubiquitin‐proteasome degradation and maintaining its protein abundance. Thus, Armcx1 and Sirt6 operate through distinct modalities—one enhancing Miro1 activity, the other preserving Miro1 stability. Whether Armcx1 and Sirt6 cooperate to regulate Miro1 in a cell‐type‐specific or injury‐stage‐specific manner remains unknown. Alternatively, whether Sirt6‐mediated deacetylation influences Armcx1‐Miro1 interaction. These possibilities warrant further exploration. Moreover, a recent study demonstrated that PRDX5 regulates Miro1 and Milton expression to promote mitochondrial transport during myotube formation, revealing that Miro1 expression is transcriptionally controlled by redox‐sensitive pathways during muscle differentiation [49]. Also, oxidative modulation of Miro1‐mediated mitochondrial trafficking is a key redox‐sensitive mechanism governing intercellular mitochondrial transfer in mesenchymal stromal cells [50]. This aligns with our finding that Sirt6 couples Miro1 regulation to the cellular redox and metabolic state during AKI, further supporting the concept that Miro1 is a central node integrating metabolic and oxidative stress signals.

Several limitations of this study should be acknowledged. First, although we have demonstrated that Sirt6 deacetylates Miro1 and identified K182 as a critical acetylation site, the specific E3 ubiquitin ligase responsible for recognizing acetylated Miro1 has not been identified. The precise molecular mechanism by which Miro1 acetylation regulates its protein stability remains to be fully elucidated. Whether acetylation at K182 directly influences Miro1 conformation, its interaction with TRAK1/TRAK2 motor adaptors, or its recognition by the ubiquitin‐proteasome system requires further structural and biochemical investigation. Second, our mitochondrial functional assays focused on measurements of respiration, ROS, and ATP production. Direct visualization of mitochondrial movement dynamics, such as live‐cell time‐lapse imaging to track mitochondrial trafficking in real time, would provide more definitive evidence for Miro1‐mediated mitochondrial transport regulation. Third, the exclusive use of male C57BL/6J mice in this study represents a notable limitation. A previous study has established Sirt6 as a key contributor to gender differences in AKI susceptibility [42]. Whether the Sirt6‐Miro1 axis exhibits similar protective effects in female mice remains unknown. Future studies incorporating both male and female animals, as well as gonadectomy and hormone replacement models, are essential to fully elucidate the gender‐specific regulation of the Sirt6‐Miro1 pathway and its therapeutic implications. Finally, while our findings were validated in two well‐established murine AKI models and in renal biopsies from ATN patients, the translational potential of targeting the Sirt6‐Miro1 axis requires further evaluation. Whether pharmacological modulation of Sirt6 deacetylase activity or Miro1 stabilization can be safely and effectively achieved in human AKI remains to be determined.

## 4 Conclusion

Collectively, our findings presented that Miro1 reduced tubular cellular injury via regulating mitochondrial homeostasis in AKI. Our work also revealed that the regulation of mitochondrial function by Miro1 may be dependent on Sirt6‐mediated deacetylation. All in all, Strategies that preferentially activate Miro1 in tubular cells may represent novel and effective approaches to prevent and treat AKI.

## 5 Materials and Methods

### 5.1 Ethics Statement

The study was approved by the Ethics Committee of the First Affiliated Hospital of Nanjing Medical University. All patients provided their written informed consent prior to inclusion in the study.

### 5.2 Reagents and Antibodies

CoCl_2_, cisplatin, wheat germ agglutinin (WGA), MitoTracker Red, and MitoSOX were purchased from Sigma‐Aldrich (St. Louis, MO). Cycloheximide (CHX), MG132, Chloroquine (CQ), OSS‐128167, UBCS039 were purchased from MCE (Shanghai, China), anti‐Sirt6, anti‐cleaved caspase3, anti‐Tom20 and anti‐acetylated‐Lysine antibodies were derived from CST (Danvers, MA, USA), anti‐Miro1, anti‐Miro2, anti‐Mic60, anti‐Mfn1, anti‐Mfn2, anti‐Sirt1 and anti‐PGC‐1α antibodies were derived from Abcam (Cambridge, UK), anti‐L‐OPA1 was provided by BD Biosciences (Franklin Lake, NJ, USA), anti‐H3K9Ac, anti‐ubiquitin (Ub) and anti‐GAPDH were provided by Proteintech (Wuhan, China), anti‐Myc, anti‐Flag were provided by Abmart (Shanghai, China). All secondary antibodies for immunoblot analysis were from Zhongshan Golden Bridge Biotechnology (Beijing, China).

### 5.3 Human Specimens

The samples of renal biopsies from patients with ATN and MCD were obtained from the Department of Nephrology, the First Affiliated Hospital of Nanjing Medical University. Diagnoses of ATN and MCD were established in accordance with renal biopsy pathology. The clinical information of these patients is provided in the Tables S1 and S2.

### 5.4 Animals

All the procedures for animal studies were approved by the Institutional Animal Care and Use Committee of Nanjing Medical University. 8–12 weeks old C57BL/6J male mice were supplied by the Experimental Animal Center of Nanjing Medical University. Renal IR injury was induced by bilateral kidney pedicle clamping for 30 min, followed by reperfusion for 24 h. For cisplatin‐induced AKI, cisplatin solution was intraperitoneally injected into the mice at a dose of 30 mg/kg. Mice were sacrificed 3 days later, and the kidneys and blood samples were harvested.

### 5.5 Generation of Renal Proximal Tubular Miro1 Conditional Knockin Mouse Strains

We bred mice with loxP‐flanked Miro1 alleles (Miro1^fl/fl^ mice) with mice that expressed Cre recombinase selectively in proximal tubular epithelia (Cre recombinase under the control of KAP‐Cre). Routine PCR was used to genotype the mice.

### 5.6 Cell Culture and Treatment

Human kidney tubular cells (HK2) were purchased from ATCC. Cells were cultured in DMEM/F12 supplemented with 10% FBS, penicillin (100 U/ml) and streptomycin (100 mg/ml). Small interfering RNA was obtained from GeneChem (Shanghai, China) to knock down the Miro1 gene. Cells were treated with serum‐free medium containing 200 µM CoCl_2_ for 12 h, followed by replacement with complete medium and further incubation for 24 h to mimic hypoxia‐reoxygenation conditions in vitro. For Miro1 overexpression, the human Miro1 gene cloned into a pcDNA3.1 vector was purchased from Addgene (plasmid #47888). Transfection was performed with Lipofectamine 2000 reagent (Invitrogen, Carlsbad, CA) following the manufacturer's instructions. Cells were transfected with the siRNA or plasmid for one day before CoCl_2_ or cisplatin treatment.

### 5.7 Renal Histology

Tissues were embedded in paraffin and kidney sections were cut into 2 µm and stained with periodic acid‐Schiff (PAS), according to a standard protocol. Tubular damage scores were assessed by the percentage of injured tubules described previously.

### 5.8 Kidney Function

Serum creatinine (Scr) levels were detected by a quantitative colorimetric method using a Creatinine Assay Kit supplied by Jiancheng Bioengineering Institute (Nanjing, China) following the manufacturer's instructions. Blood urea nitrogen (BUN) levels were detected by a quantitative Urea Assay Kit EIABUN (Thermo Fisher Scientific, Waltham, MA) according to the manufacturer's instructions.

### 5.9 Western Blotting

Total protein was isolated from HK2 cells or kidney tissues using the RIPA buffer method according to the manufacturer's protocol. Thirty micrograms of protein were separated by 10% SDS‐PAGE and were transferred onto nitrocellulose membranes. After blocking with 5% milk in PBS for 1 h, the corresponding primary anti‐Miro1 (1:1000), anti‐Miro2 (1:1000), anti‐cleaved caspase 3 (1:1000), anti‐Mic60 (1:1000), anti‐L‐OPA1 (1:1000), anti‐Mfn1 (1:1000), anti‐Mfn2 (1:1000), anti‐Sirt1 (1:1000), anti‐PGC‐1α (1:1000), anti‐Tom20 (1:1000), anti‐Sirt6 (1:1000), anti‐acetylated‐Lysine (1:1000), anti‐Ub (1:5000), anti‐Myc (1:5000), anti‐Flag (1:5000) and anti‐GAPDH (1:100000) antibodies were added, and the membranes were incubated overnight at 4°C. Then, the membranes were incubated with secondary antibodies accordingly and visualized with enhanced chemiluminescence reagent (Fude Biotechnology, Hangzhou, China). The gray value of the bands of interest was analyzed using Image J software.

### 5.10 Apoptosis

For in vitro apoptosis analysis, Annexin V‐FITC Apoptosis Detection Kit (BD Biosciences) was applied to evaluate HK2 cell apoptosis following the manufacturer's protocols. After treatment, HK2 cells were resuspended with FITC‐conjugated annexin V and propidium iodide (PI) for 10 min. After that, flow cytometry was performed within 1 h. In situ detection of cell death was performed using a commercially available TUNEL kit (Roche, Germany) following the manufacturer's protocols.

### 5.11 Immunohistochemistry

Paraffin kidney sections of each specimen were cut at a thickness of 4 µm and deparaffinized and rehydrated. After antigen retrieval and blocking endogenous peroxidase, the tissues were then incubated with Miro1 antibody (1:200) or Sirt6 antibody (1:50) overnight at 4°C. After three washes with PBS, the biotinylated goat anti‐rabbit secondary antibody was applied, and the signals were observed under a light microscope.

### 5.12 Immunofluorescence

Paraffin kidney sections of each specimen were cut at a thickness of 4 µm and deparaffinized and rehydrated. After antigen retrieval, blocking endogenous peroxidase and blocking with 10% BSA for 1 h, the sections were incubated with the indicated primary antibodies overnight at 4°C. After this, the sections were washed and stained with fluorescence‐conjugated secondary antibodies. Then the nuclei were co‐stained with DAPI. The acquisition of pictures was performed utilizing a microscope (Leica, Germany).

### 5.13 Measurement of Oxygen Consumption Rate (OCR)

HK2 cells were counted and seeded on XF‐96‐well culture microplates (1.5 × 10^4^ cells/well). After treatment, oxygen consumption was measured using the Seahorse Bioscience metabolic analyzer as previously described. Reagents were optimized using the Mitostress kit from Seahorse Bioscience (Agilent, 100850‐001). Basal mitochondrial respiration, maximal respiration, and ATP‐linked respiration were determined as described [51].

### 5.14 Mitochondrial Morphology

The change in mitochondrial morphology in vitro was detected by using MitoTracker Red. After treatment, HK2 cells were stained with MitoTracker Red (10 nM) for 10 min before confocal microscopic analysis described previously.

### 5.15 Mitochondrial Distribution

Live HK2 cells were co‐incubated with 10 nM MitoTracker Red, 2.5 µg/mL WGA, and 1×Hoechst 33342 diluted in HBSS buffer at 37°C for 10 min. Hoechst 33342 was purchased from Beyotime (Shanghai, China). Cells were then washed three times with HBSS and examined for mitochondrial distribution using confocal microscopy.

### 5.16 Mitochondrial Reactive Oxygen Species (ROS) Production

The mitochondrial ROS levels were measured using MitoSOX. HK2 cells were reacted with MitoSOX (5 µmol/L) for 10 min at 37°C with or without CoCl_2_ treatment following the manufacturer's instructions.

### 5.17 ATP Generation

ATP levels were determined using a luciferase‐based ATP Determination Kit (Beyotime, Nanjing, China) according to the manufacturer's protocol. ATP content was expressed as nmol/mg protein.

### 5.18 Mitochondrial DNA (mtDNA) Copy Number

Isolation of DNA from HK2 cells was carried out using a Universal Genomic DNA Kit (CW2298S, CWBIO, Beijing, China) according to the manufacturer's protocol. Relative levels of mtDNA copy number were determined by qPCR as previously described. Relative mtDNA copy number was presented as the mtDNA to nuclear DNA ratio.

### 5.19 Electron Microscopy

Renal tissues were fixed in 2.5% glutaraldehyde, then fixed in 2% osmium tetroxide, dehydrated with graded alcohols, and immersed in epoxy resin. Prepared ultra‐thin sections were observed using TEM (JEOL JEM‐1230, Japan).

### 5.20 Shotgun Proteomics

Label‐free quantitative proteomics were performed by Shanghai Genechem Co., Ltd as previously published [52]. HK2 cells were transfected with Miro1 or vector plasmid. Whole cell lysate was immunoprecipitated with Miro1 antibody. Four independent immunoprecipitated protein extracts from Miro1 overexpression and control groups were analyzed by LC‐MS/MS, and the raw data files were subjected to data analysis for peptide and protein identification.

### 5.21 Co‐Immunoprecipitation

Whole cell lysate was immunoprecipitated with the indicated antibody or respective IgG with Protein G magnetic beads. The antibodies used for immunoprecipitation were anti‐Miro1, anti‐Sirt6, anti‐acetylated‐Lysine, anti‐Myc, anti‐Flag, and normal IgG. The immunoprecipitates were eluted in loading buffer and subjected to western blotting.

### 5.22 GST Pull‐Down Assay

Recombinant GST and GST‐Miro1 fusion protein were obtained from GenScript (Nanjing, China). GST pull‐down kit was purchased from Abmart (Shanghai, China). GST pull‐down assay was performed according to the manufacturer's instructions. Briefly, 10 µg of GST or GST‐Miro1 was immobilized on 40 µL of glutathione‐Sepharose beads in binding buffer with gentle rotation at 4°C overnight. The beads were then washed three times, and 500 µg of HK2 cell lysates was added to the beads for incubation at 4°C overnight. After washing three times, bound proteins were eluted by boiling in 30 µL of 1× SDS loading buffer, separated by SDS‐PAGE, and subjected to western blotting with specific antibodies against GST and the prey protein Sirt6.

### 5.23 Identification of Acetylation Sites in Miro1

To identify the acetylation sites, proteins immunoprecipitated by anti‐Myc antibody in Miro1 overexpressing cells were separated by SDS‐PAGE and subjected to in‐gel digestion, followed by liquid chromatography‐tandem mass spectrometry (LC‐MS/MS) analysis (Applied Protein Technology, Shanghai, China). The MS raw data were searched against the corresponding protein database using MaxQuant. For validation, acetylation‐deficient (lysine‐to‐arginine, K→R) point mutations were introduced into the target gene using site‐directed mutagenesis (IBSBIO, Shanghai, China). The wild‐type and the resulting mutant constructs were transiently transfected into HK2 cells. After 24 h, cells were harvested, and the acetylation levels of wild‐type and mutant proteins were assessed by western blotting using an anti‐Myc antibody following immunoprecipitation by an anti‐acetylated lysine antibody. Protein expression levels were verified using an anti‐Myc antibody.

### 5.24 Statistical Analysis

Values were presented as means ± standard error of the mean (SEM). Statistical analysis was performed by one‐way ANOVA and Bonferroni tests using SPSS for Windows version 22.0. Differences were considered significant at *p*<0.05.

## Author Contributions

YGY and HJM contributed to the conceptualization and design of the study. LW and QL performed the study. YYX, ZTW, HS, and YFW performed data acquisition, analysis, and interpretation. YGY and LW drafted the manuscript and figures. ZMH and FL collected human kidney samples. BZ analyzed histology images. HWL revised the manuscript. The project was supervised by HJM and CYX. All authors actively discussed and reviewed the manuscript.

## Conflicts of Interest

The authors declare no conflict of interest.

## Supporting information




**Supporting File**: advs76824‐sup‐0001‐SuppMat1.pdf.

## Data Availability

All data generated during these studies are included in the text, figures, and tables of this article and electronic supplementary material. Source data or materials will be supplied by the corresponding authors with reasonable request.
