# Supplementary material for: Miro1 Protects Against Acute Kidney Injury Through Modulating Mitochondrial Homeostasis via Interaction With Sirt6
Source: Adv Sci (Weinh). 2026 Aug 5;13(59):e76824. doi: 10.1002/advs.76824 (PMC13440030; doi:10.1002/advs.76824)

Miro1 protects against acute kidney injury through modulating mitochondrial homeostasis via interaction with Sirt6

Lin Wu[1#], Qing Li[1#], Yiyang Xu[1], Zitao Wang[1], Hua Shu[1], Yangfan Wu[1], Fang Lu[1], Zhimin Huang[1], Bo Zhang[1], Hongwei Liang[2], Changying Xing[1], Huijuan Mao[1], and Yanggang Yuan[1]**

[#] L. W. and Q. L. contributed equally to this work.

* Senior authors with equal contribution to this study.

[1]Department of Nephrology, the First Affiliated Hospital of Nanjing Medical University, Nanjing Medical University, Nanjing, China

Email: Yanggang Yuan, ygyuan@njmu.edu.cn; Huijuan Mao,13851746819@126.com

[2]School of Life Science and Technology, China Pharmaceutical University, Nanjing, Jiangsu, China.

Supplementary Methods

Single-cell RNA sequencing (scRNA-seq) analysis

For single-cell RNA sequencing (scRNA-seq) analysis, the mouse kidney ischemia-reperfusion injury (IRI) dataset GSE139107 (https://www.ncbi.nlm.nih.gov/geo/query/acc.cgi?acc=GSE139107) containing 24 samples (including Sham control and 4 hours, 12 hours, 2 days, 14 days, and 6 weeks post-IRI) was retrieved from the NCBI GEO database (https://www.ncbi.nlm.nih.gov/geo/), and human kidney datasets were obtained from the KPMP database (https://www.kpmp.org/), which comprised a healthy reference group (10 KPMP healthy living donor protocol biopsies, 5 KPMP percutaneous nephrolithotomy protocol biopsies, 16 tumor nephrectomy samples from 5 participants, and 20 living donor biopsies from 18 participants in the Human Cell Atlas) and an acute kidney injury (AKI) group (20 KPMP AKI protocol biopsy samples). Single-cell objects were constructed using Seurat (v4.0.2), followed by rigorous quality control where cells with mitochondrial content > 25%, genes expressed in fewer than 5 cells, and doublets identified by DoubletFinder (v2.0.4) were excluded. After normalization, the Harmony algorithm was applied to remove batch effects across datasets, and renal tubular cell subtypes (e.g., PT-S1/S2/S3, injured/repairing PT, and failed-repair PT) were annotated based on reference marker genes from a previously published study (https://doi.org/10.1101/2024.01.20.576421). Finally, candidate gene expression (e.g., Rhot1/2 and the Sirt family) across various states and time points was visualized in dot plots generated via the DotPlot function in Seurat and customized using ggplot2, where the dot size represents the percentage of expressing cells (Percent Expressed) and the color intensity represents the scaled mean expression level (Average Expression).

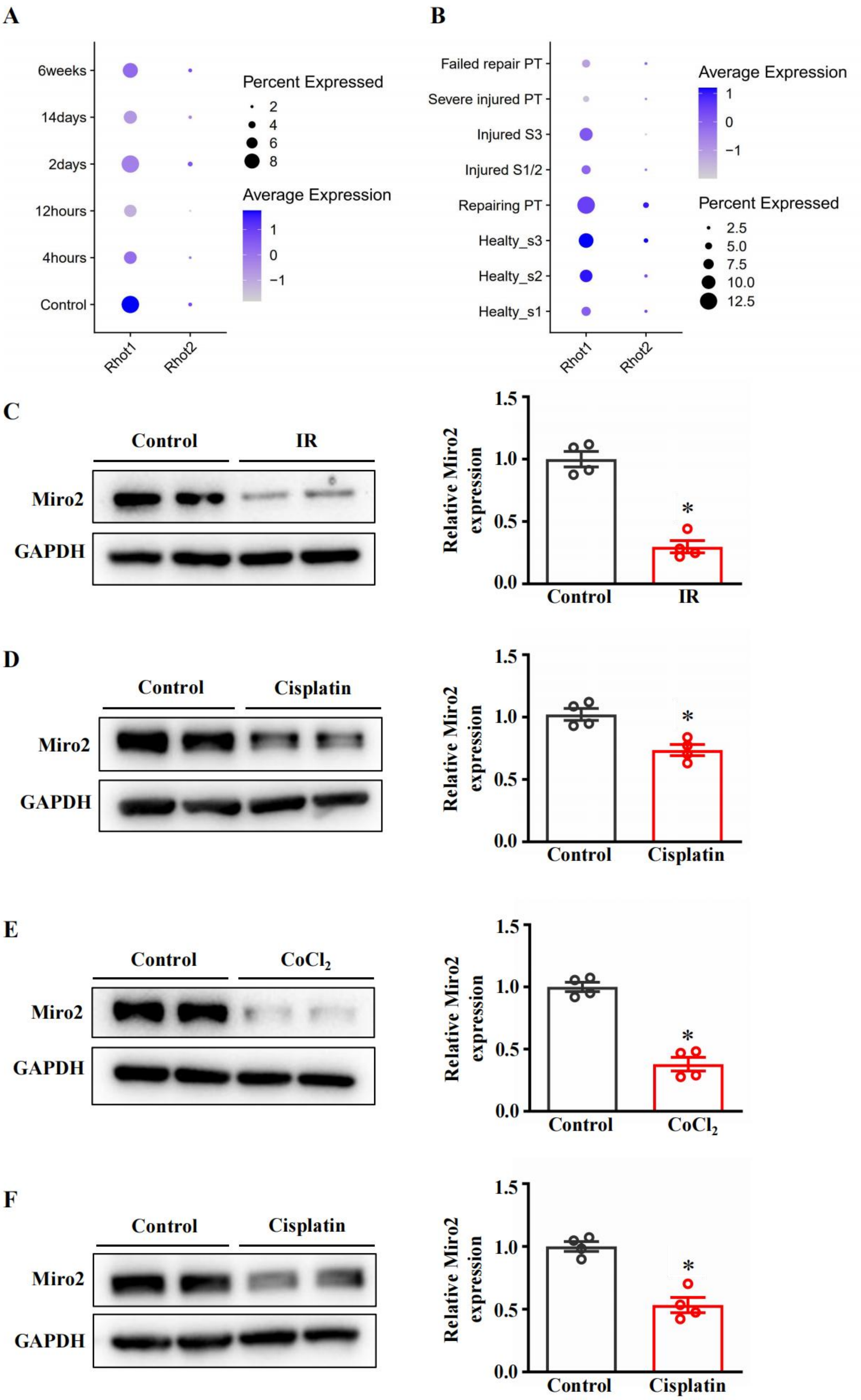
A
6weeks
14days
2days
12hours
4hours
Control
Rhot1
Rhot2
Percent Expressed
2
4
6
8
Average Expression
1
0
−1
B
Failed repair PT
Severe injured PT
Injured S3
Injured S1/2
Repairing PT
Healty_s3
Healty_s2
Healty_s1
Rhot1
Rhot2
Average Expression
1
0
−1
Percent Expressed
2.5
5.0
7.5
10.0
12.5
C
Control
IR
Miro2
GAPDH
Relative Miro2 expression
1.5
1.0
0.5
0.0
Control
IR
D
Control
Cisplatin
Miro2
GAPDH
Relative Miro2 expression
1.5
1.0
0.5
0.0
Control
Cisplatin
E
Control
CoCl2
Miro2
GAPDH
Relative Miro2 expression
1.5
1.0
0.5
0.0
Control
CoCl2
F
Control
Cisplatin
Miro2
GAPDH
Relative Miro2 expression
1.5
1.0
0.5
0.0
Control
Cisplatin

Supplementary Figure 1. (A-B) Expression of Rhot1 and Rhot2 in mouse kidney in IR model at different time points (A) and various states in renal tubular cell subtypes (B) retrieved from the original sequencing database GSE139107. The genes Rhot1 and Rhot2 encode the proteins Miro1 and Miro2, respectively. (C) Western blotting and densitometry analysis of Miro2 in kidney cortex of control and IR-induced mice. (D) Western blotting and densitometry analysis of Miro2 in kidney cortex of control and cispatin-induced mice. (E) Western blotting and densitometry analysis of Miro2 in cell lysate of control and $CoCl_2$-induced HK2 cells. (F) Western blotting and densitometry analysis of Miro2 in cell lysate of control and cispatin-induced HK2 cells. Data are shown as means ± SEM (n=4). $*p < 0.05$ vs. control group.

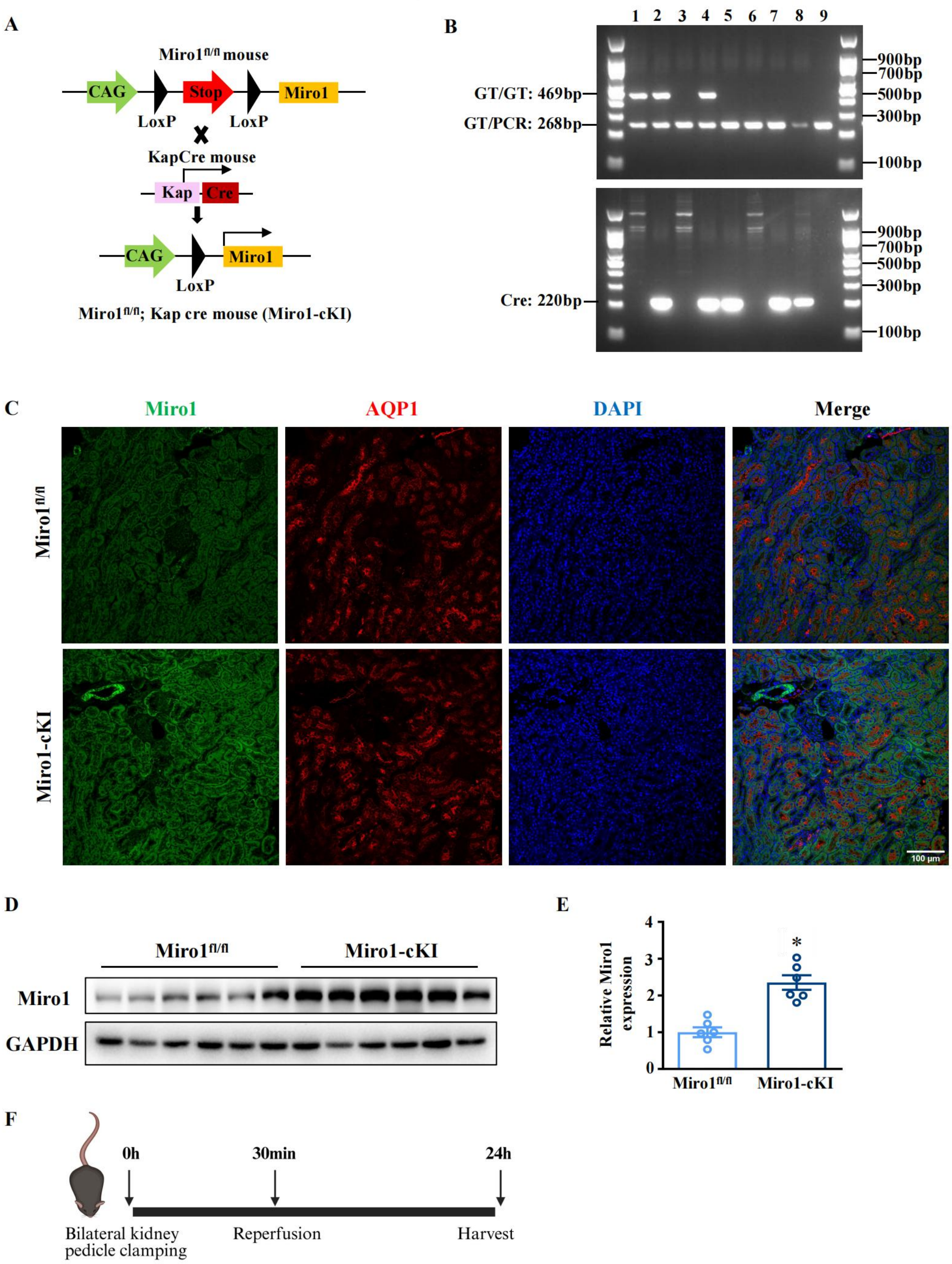

Supplementary Figure 2. (A) Scheme representing the approach to generating Miro1 conditional knockin mice. (B) Genotypes of the mice are determined by PCR. (C) Immunofluorescence staining of Miro1 and AQP1 in Miro1$^{fl/fl}$ and Miro1-cKI mice. Scale bar, 100 μm. (D) Western blotting of Miro1 in Miro1$^{fl/fl}$ and Miro1-cKI mice. (E) Densitometry analysis of Miro1. Data are shown as means ± SEM (n = 6). $p < 0.05$ vs. Miro1$^{fl/fl}$. (F) A flowchart for *in vivo* IR models.

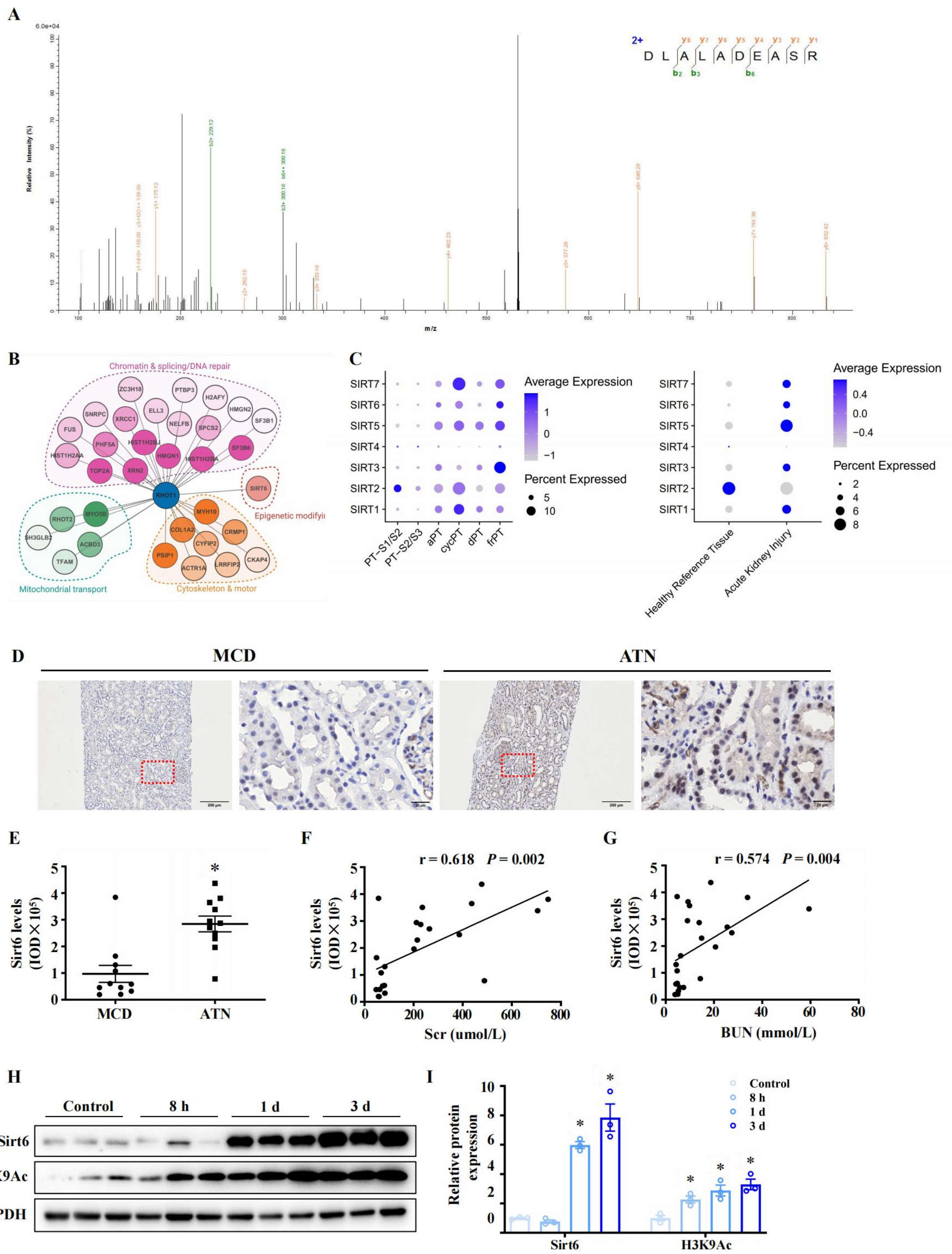

Supplementary Figure 3. (A) Representative MS/MS spectra of Sirt6 peptides identified from the co-IP products. (B) Protein-protein interaction (PPI) network of RHOT1 (Miro1) interacting partners. The network illustrates the candidate proteins interacting with RHOT1 (Miro1) identified by immunoprecipitation-mass spectrometry (IP-MS). A Mascot score threshold of >20 was applied for screening interacting partners, which are grouped into four functional modules: Chromatin & splicing/DNA repair (purple), Mitochondrial transport (green), Cytoskeleton & motor

splicing/DNA repair (purple), Mitochondrial transport (green), Cytoskeleton & motor (orange), and Epigenetic modifying (red). Darker colors indicate higher scores, reflecting stronger confidence or higher abundance in mass spectrometry identification. (C) Expression of Sirt1-Sirt7 in different states of renal tubular cell subtypes in human kidney datasets obtained from the KPMP database. aPT: adaptive proximal tubule; cycPT: cycling proximal tubule; dPT: degenerative proximal tubule; frPT: failed repair proximal tubule. These four subtypes represent injured tubules. (D) A representative photomicrograph of Sirt6 immunohistochemical staining in renal biopsies tissue from patients with acute tubular necrosis (ATN, n=12) and patients with minimal change disease (MCD, n=11) as the control. The patient samples employed here were from the identical cohort used for Miro1 immunohistochemistry..Scale bar of low magnification: 200 μm; Scale bar of high magnification: 20 μm. (E) Densitometry analysis of Sirt6 protein level. Data are presented as means ± SEM. $*p < 0.05$ vs. MCD. (F-G) Correlation analysis between Sirt6 protien levels and serum creatinine (Scr, F) and BUN (G) levels in patients. (H) Western blotting of Sirt6 and H3K9Ac in kidney cortex of control and IR-induced mice at different time points. (I) Densitometry analysis of Sirt6 and H3K9Ac. Data are expressed as the means ± SEM (n=3). $*p < 0.05$ vs. control group.

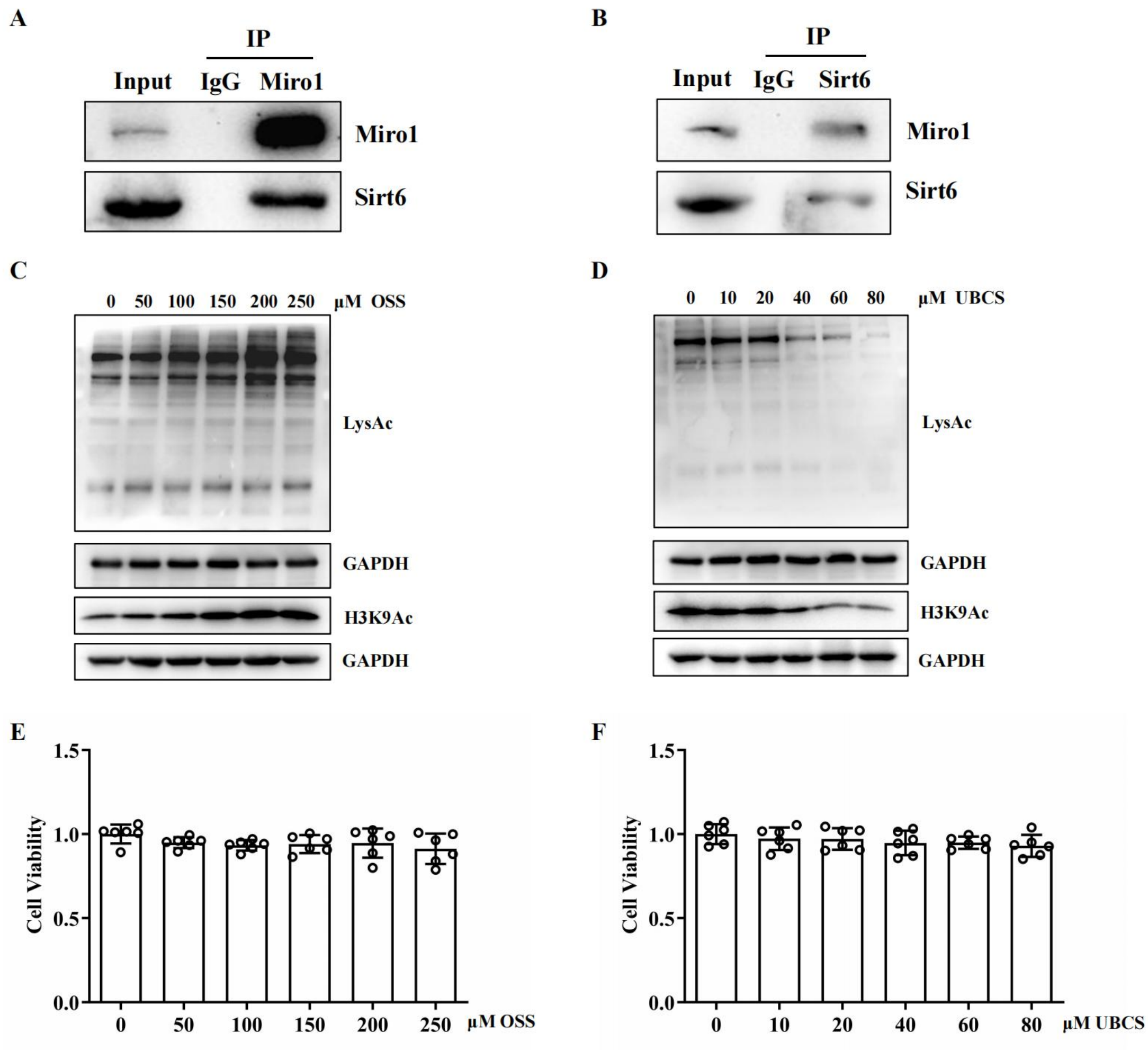

Supplementary Figure 4. (A-B) The interaction of Miro1 and Sirt6 in vivo. kidney cortex of normal mice were lysed and subjected to co-IP with anti-Miro1 antibody (A) and anti-Sirt6 antibody (B), respectively. IgG was used as a negative control. Immunoprecipitated Miro1 and Sirt6 were detected by western blotting. (C-D) Dose determination of OSS and UBCS. Assay of total acetylated-lysine and H3K9Ac levels under different doses of OSS (C) and UBCS (D) by Western blotting. (E-F) Safety evaluation of OSS (E) and UBCS (F). HK2 cells were treated with different doses of OSS and UBCS for 24 h, then cell viability was assayed by CCK-8 kit. Data are expressed as the means ± SEM (n=6).

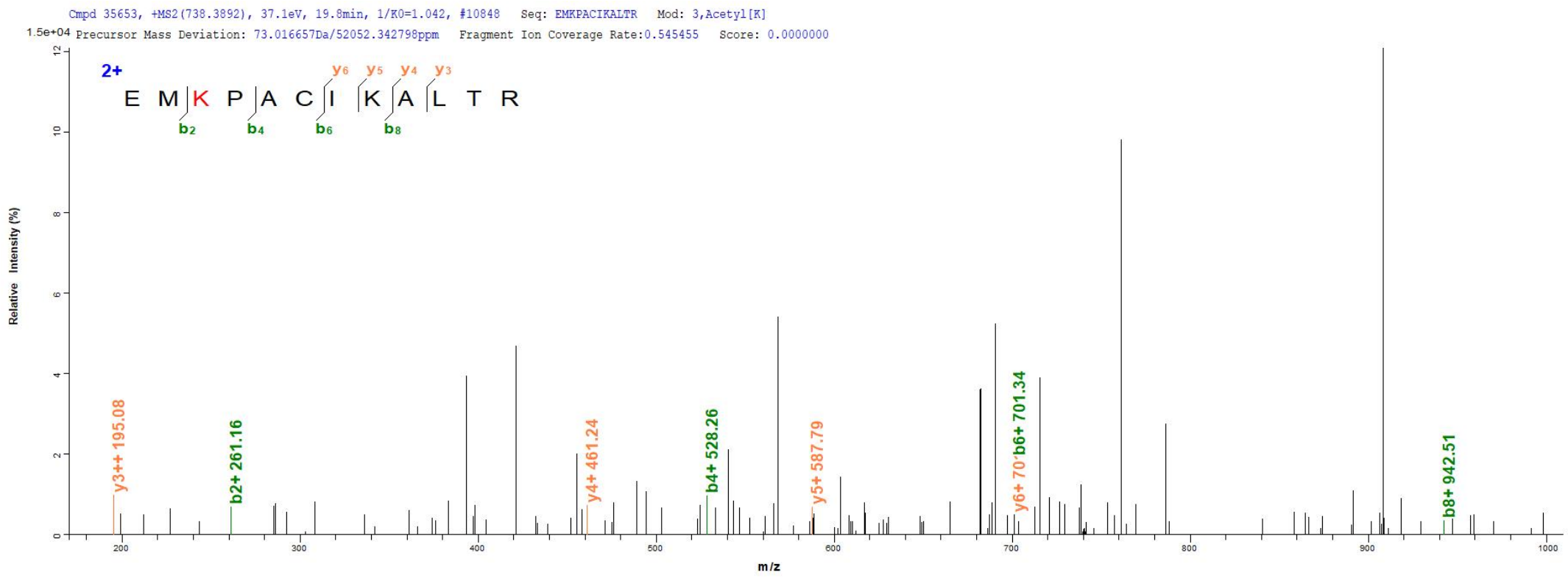

Supplementary Figure 5. Representative MS/MS spectra of Miro1 peptides identified from the co-IP products. The upper left mass spectrum shows the acetylated Miro1 peptide, where the third amino acid corresponds to lysine 182 of the protein

Supplementary Table 1. Baseline Characteristics of Patients with ATN and MCD

Characteristics	ATN Group (n=12)	MCD Group (n=11)	*p*-value
**Demographics**			
Male, n (%)	6 (50.0)	7 (63.6)	0.701
Age (years)	51.5 (40.3, 64.3)	29.0 (27.0, 37.0)	**0.006**
**Laboratory Parameters**			
Hb (g/L)	101.5 (80.5, 127.8)	141.0 (128.0, 152.0)	**0.004**
Alb (g/L)	29.3 (24.5, 37.0)	42.6 (39.5, 45.7)	**0.007**
FBG (mmol/L)	4.8 (4.3, 5.7)	4.7 (4.0, 4.8)	0.059
TC (mmol/L)	4.8 (3.8, 5.6)	5.0 (3.0, 5.5)	0.786
TG (mmol/L)	2.3 (1.8, 3.2)	1.1 (0.8, 1.7)	**<0.001**
ALT (U/L)	21.6 (11.7, 32.6)	19.5 (12.8, 27.3)	0.695
AST (U/L)	25.0 (18.3, 36.2)	19.4 (15.5, 21.7)	0.190
**Renal Function**			
SCr (μmol/L)	325.5 (218.5, 485.9)	62.1 (57.3, 80.6)	**<0.001**
BUN (mmol/L)	16.8 (13.4, 26.9)	4.8 (4.3, 5.9)	**<0.001**
UA (μmol/L)	436.0 (342.1, 621.9)	366.0 (293.0, 387.0)	0.235
**Other Biochemistry**			
RBP (μg/mL)	81.0 (55.6, 94.9)	39.2 (32.9, 43.9)	**<0.001**
Ca (mmol/L)	2.1 (1.9, 2.3)	2.4 (2.1, 2.4)	**0.032**
P (mmol/L)	1.4 (1.1, 1.7)	1.2 (1.1, 1.5)	0.566
K (mmol/L)	4.1 (3.4, 4.5)	4.0 (3.9, 4.1)	0.880
Na (mmol/L)	139.3 (135.2, 141.6)	140.1 (138.6, 140.6)	0.651
Cl (mmol/L)	103.0 (100.5, 105.2)	104.7 (103.8, 107.2)	0.260

Abbreviations: ATN, acute tubular necrosis; MCD, minimal change disease; Hb, hemoglobin; Alb, albumin; FBG, fasting blood sugar; TC, total cholesterol; TG, triglyceride; ALT, alanine aminotransferase; AST, aspartate aminotransferase; SCr, serum creatinine; BUN, blood urea nitrogen; RBP, retinol-binding protein.

Data are presented as median (interquartile range [IQR]) for continuous variables and n (%) for categorical variables. Group comparisons were performed using the Mann-Whitney U test for continuous variables and Fisher's exact test for categorical variables. Bold P-values indicate $p < 0.05$.

Supplementary Table 2. Clinical characteristics of patients with ATN

Patient ID	Gender	Age	Scr (μmol/L)	BUN (mmol/L)	Cause of ATN
ATN01	male	77	747.8	33.93	Nephrotoxicity
ATN02	male	62	488.6	14.34	Ischemia
ATN03	female	65	201.9	20.65	Ischemia
ATN04	male	20	211.6	9.00	Nephrotoxicity
ATN05	female	49	469.7	13.19	Nephrotoxicity
ATN06	male	72	228.8	13.90	Nephrotoxicity
ATN07	female	44	264.5	25.50	Sepsis
ATN08	male	49	705.5	59.40	Ischemia
ATN09	female	54	386.5	27.39	Ischemia
ATN10	male	29	215.1	14.90	Ischemia
ATN11	female	55	235.9	9.90	Nephrotoxicity
ATN12	female	39	477.6	18.67	Nephrotoxicity

Figure 1E

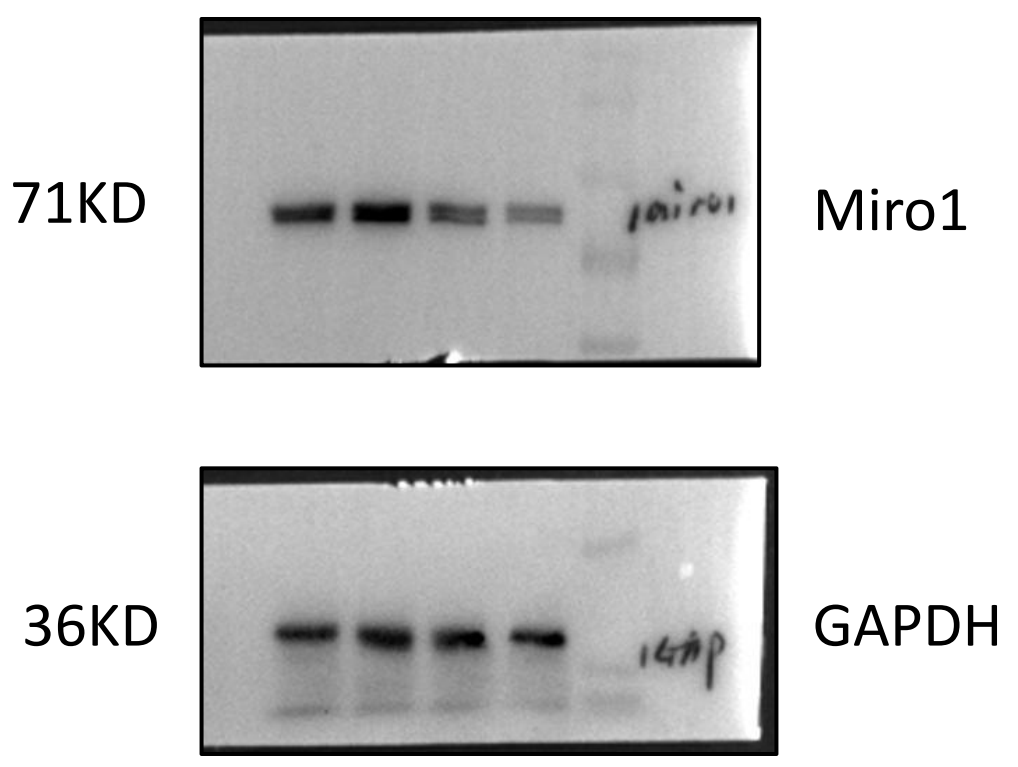

Figure 1F

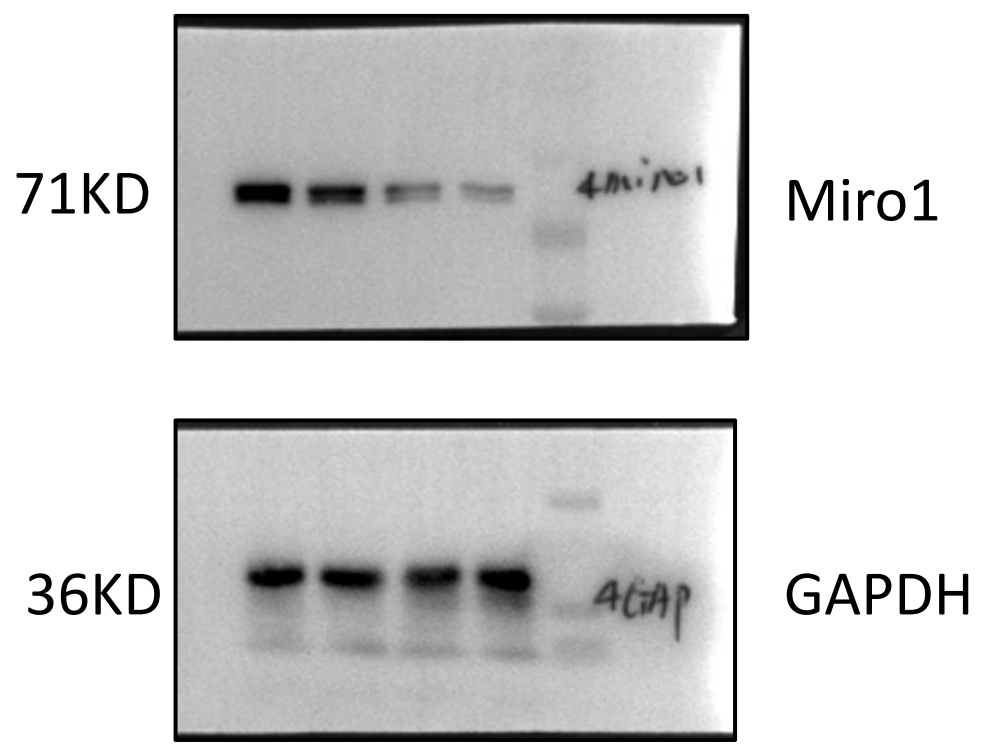

Figure 2A

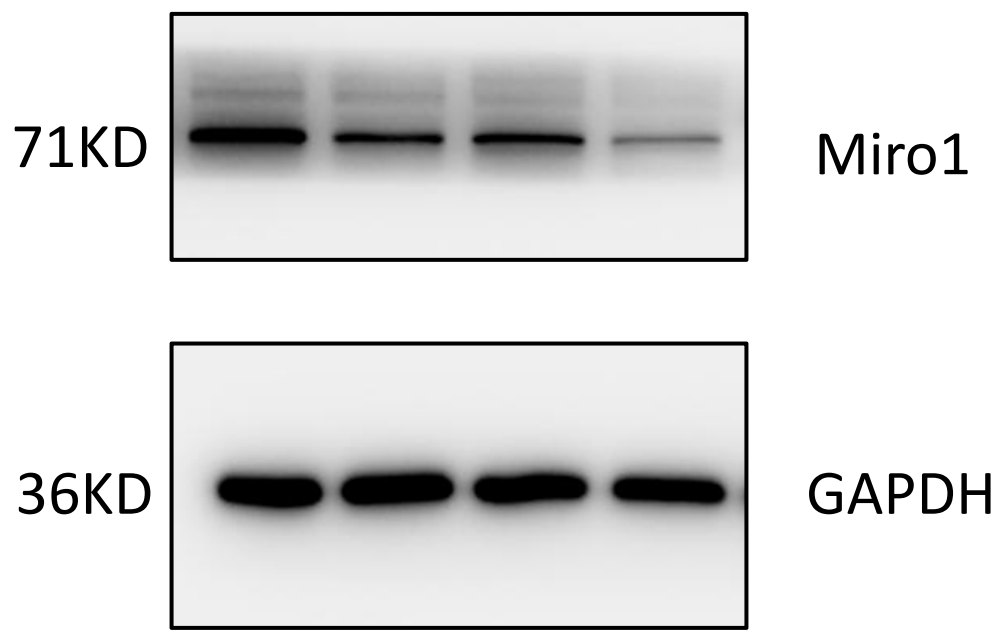

Figure 2J

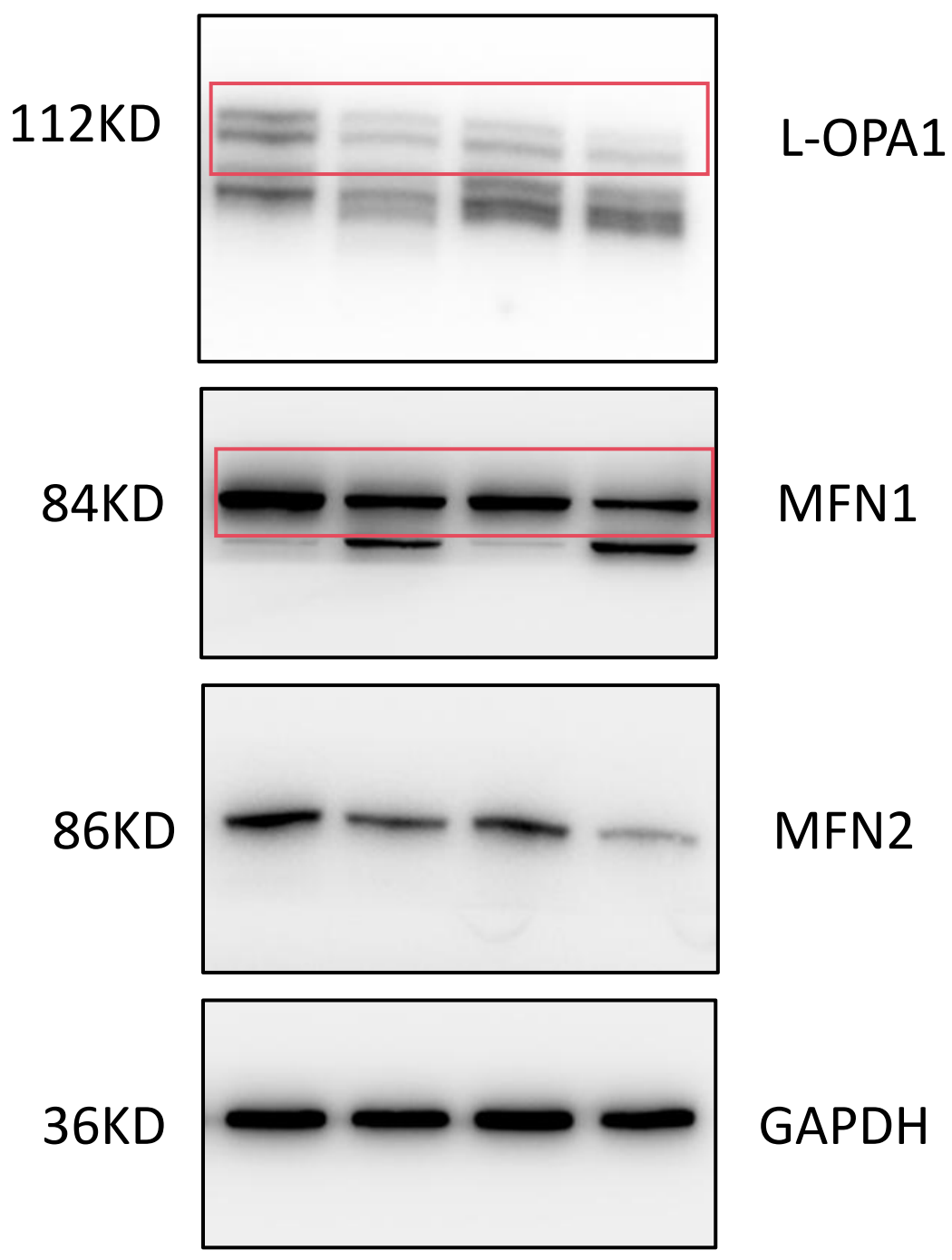

Figure 2B

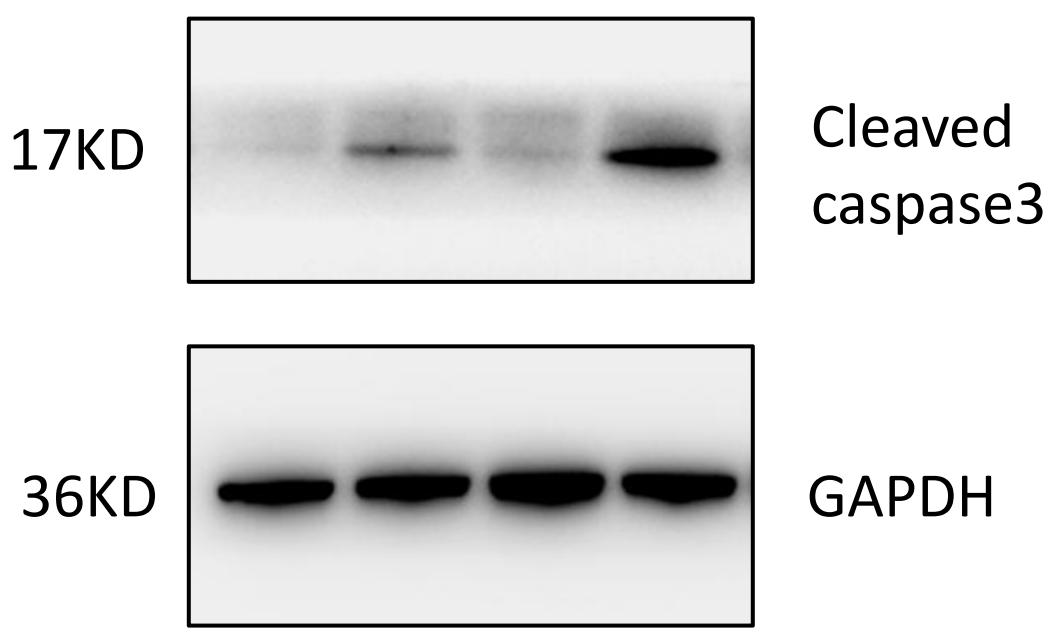

Figure 3F

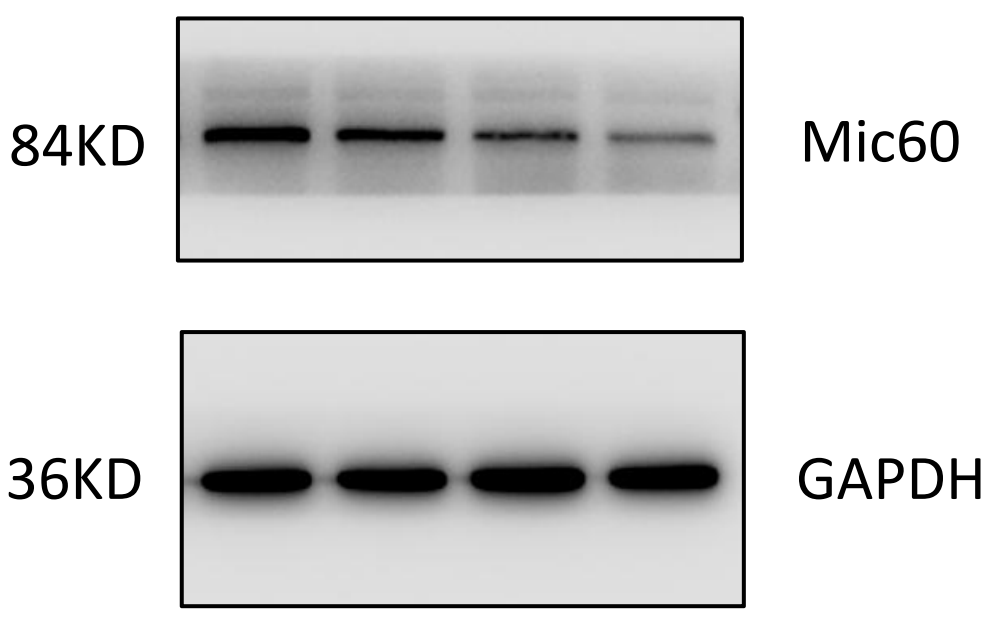

Figure 3I

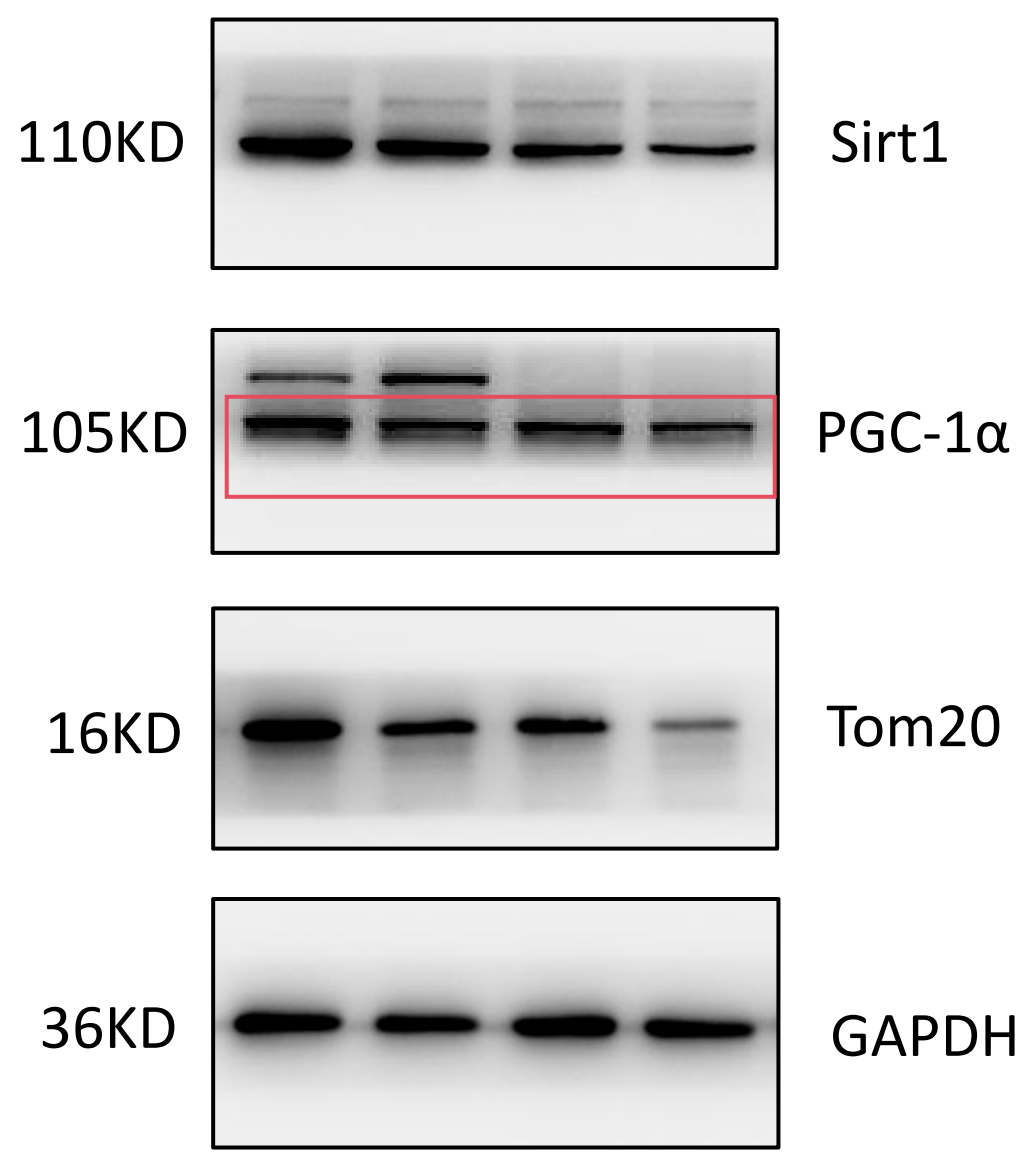

Figure 4A

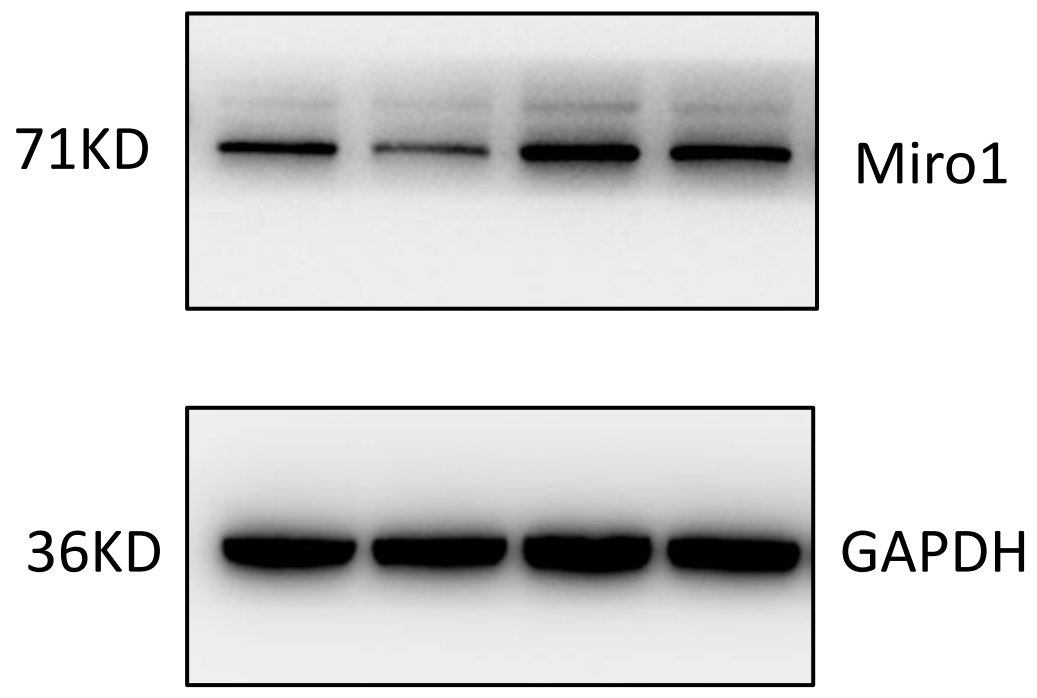

Figure 4B

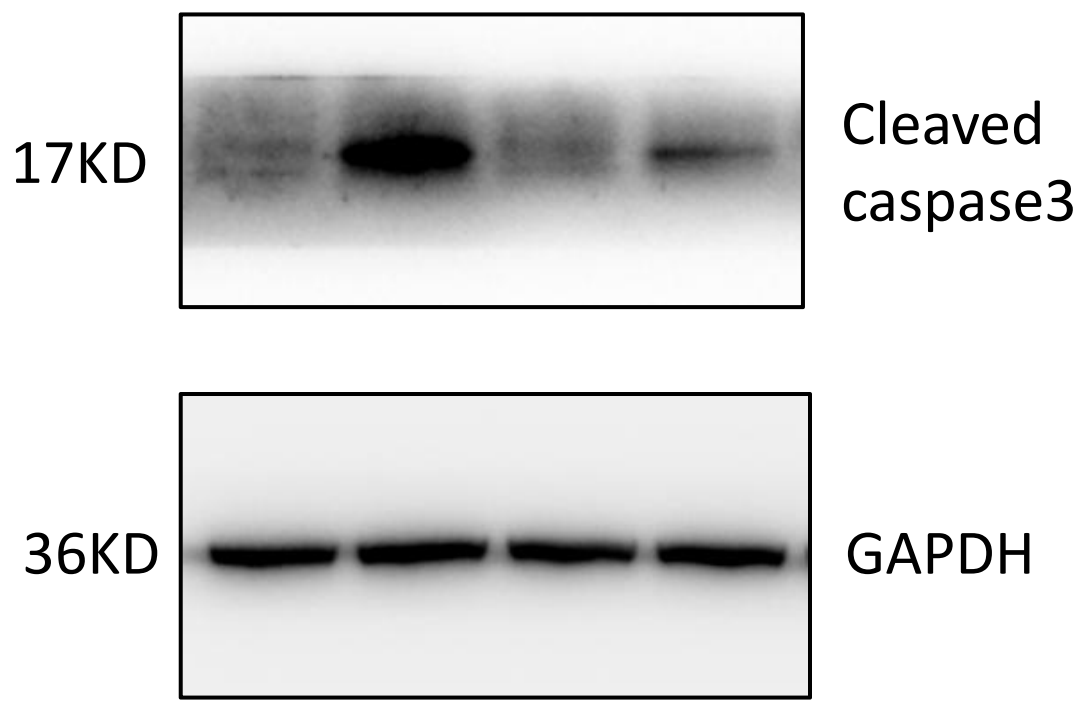

Figure 4J

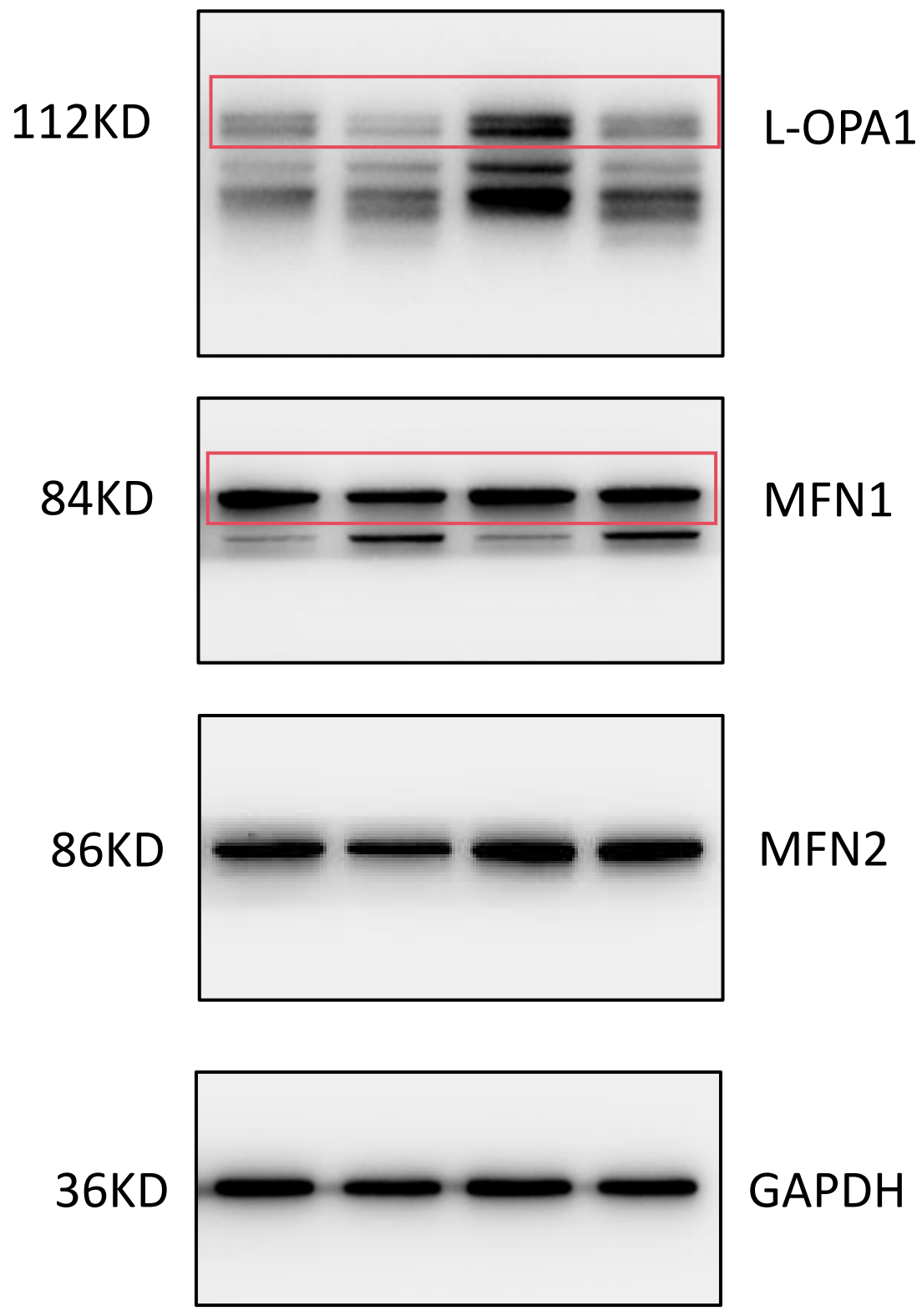

Figure 5F

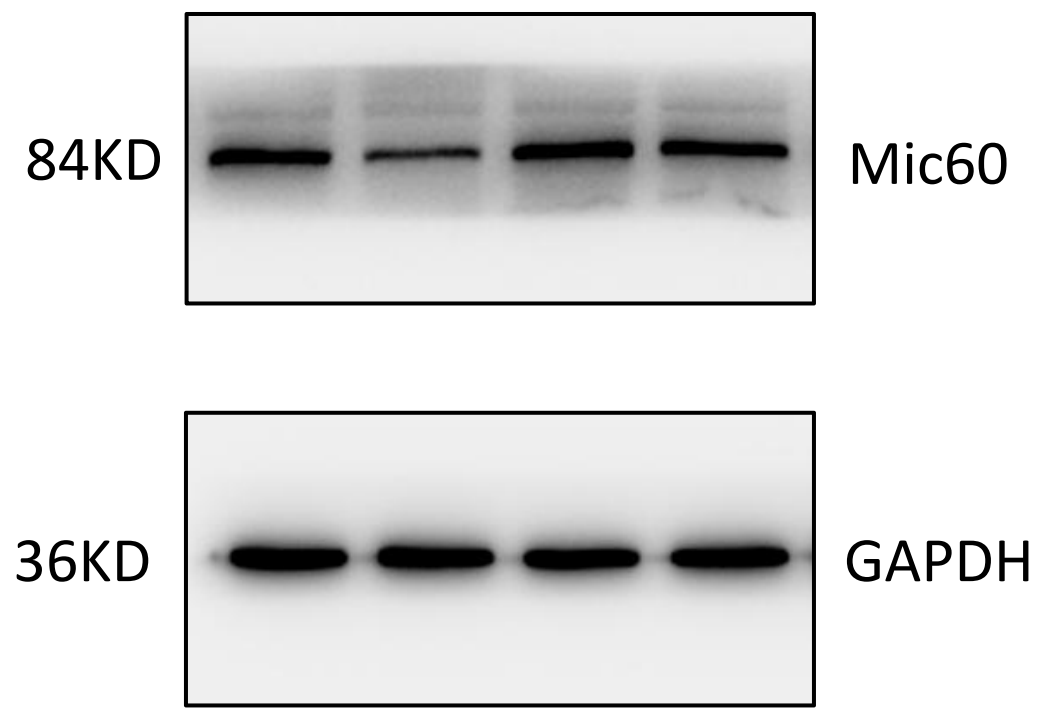

Figure 5I

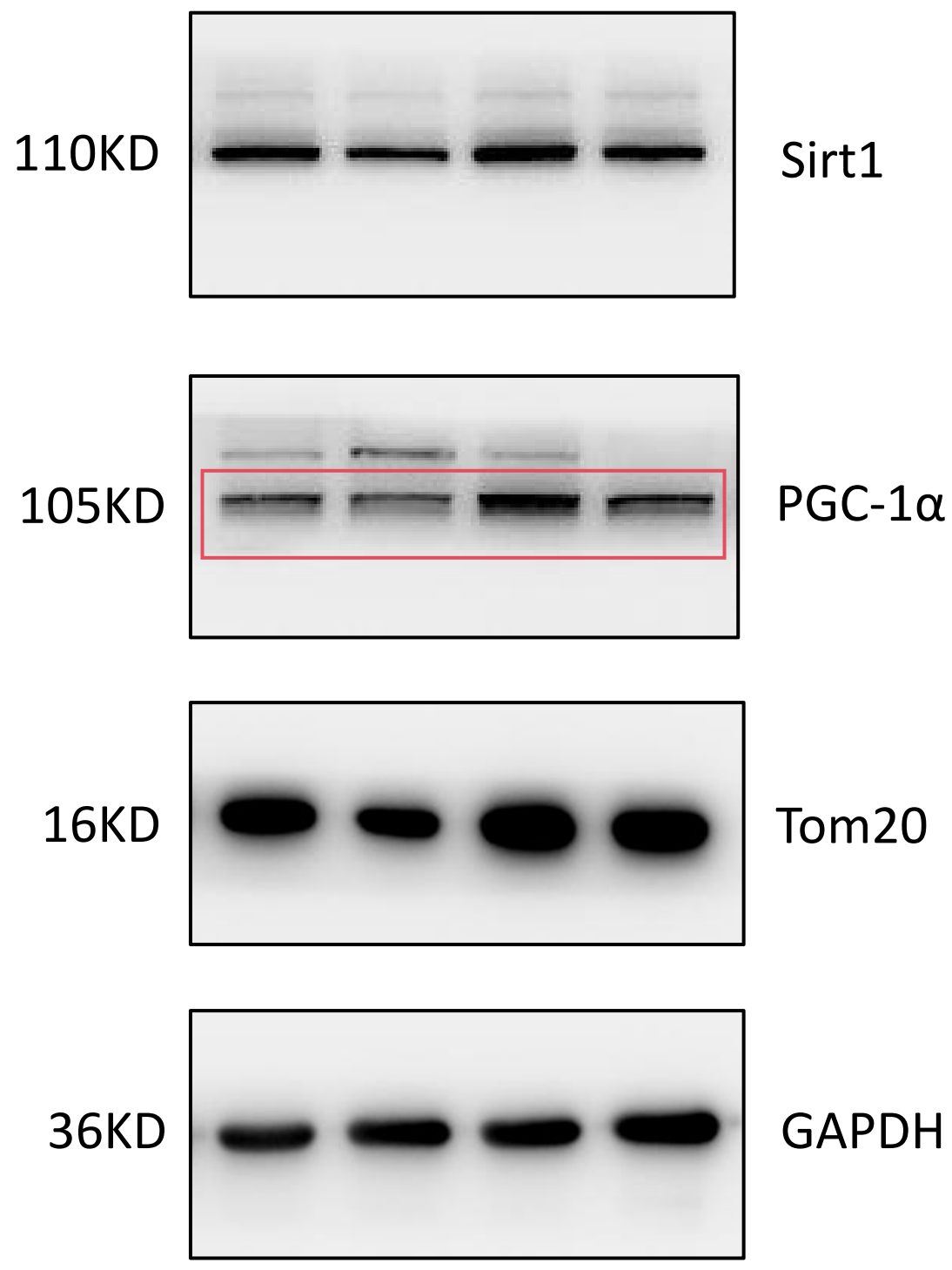

Figure 6E

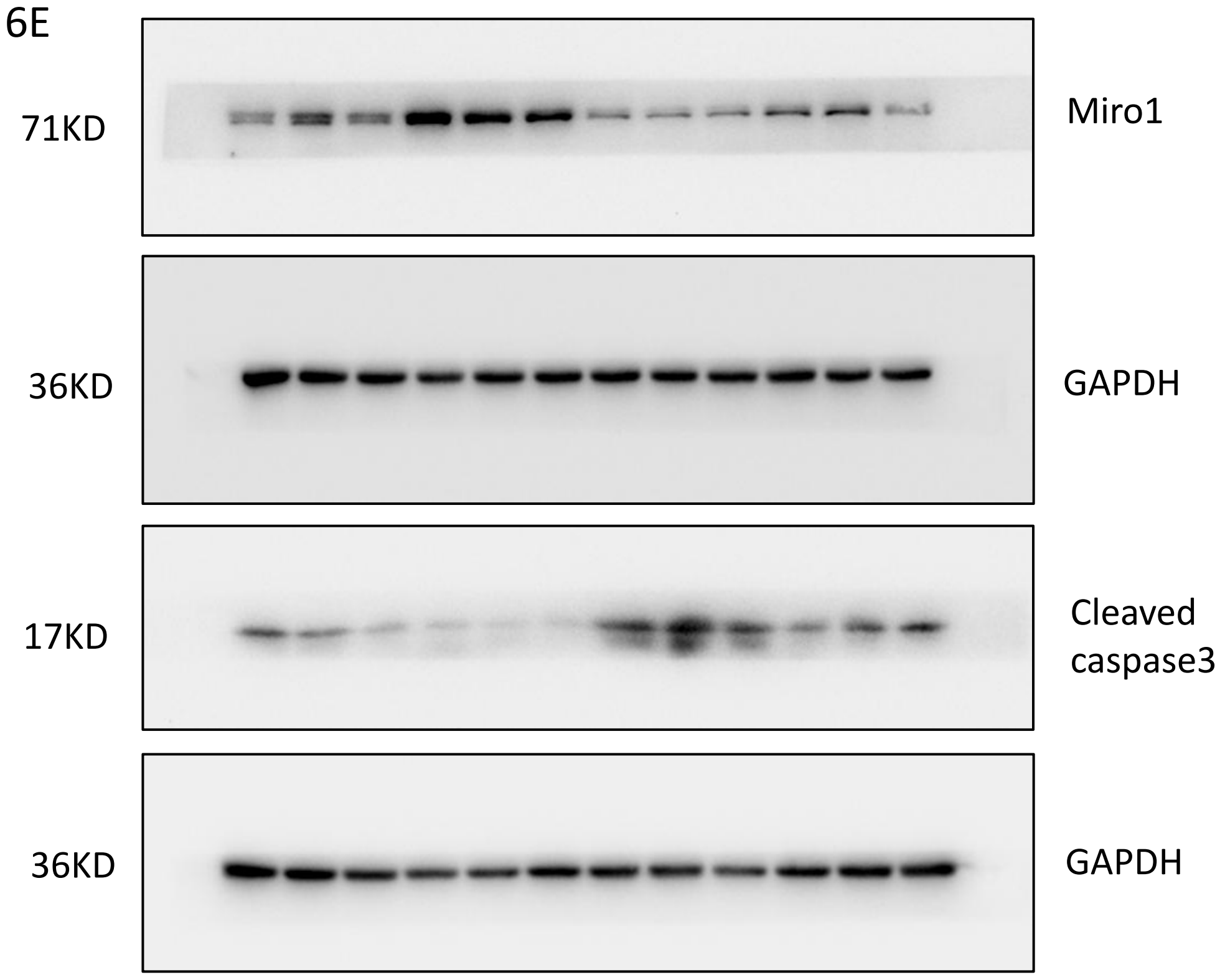

Figure 7A

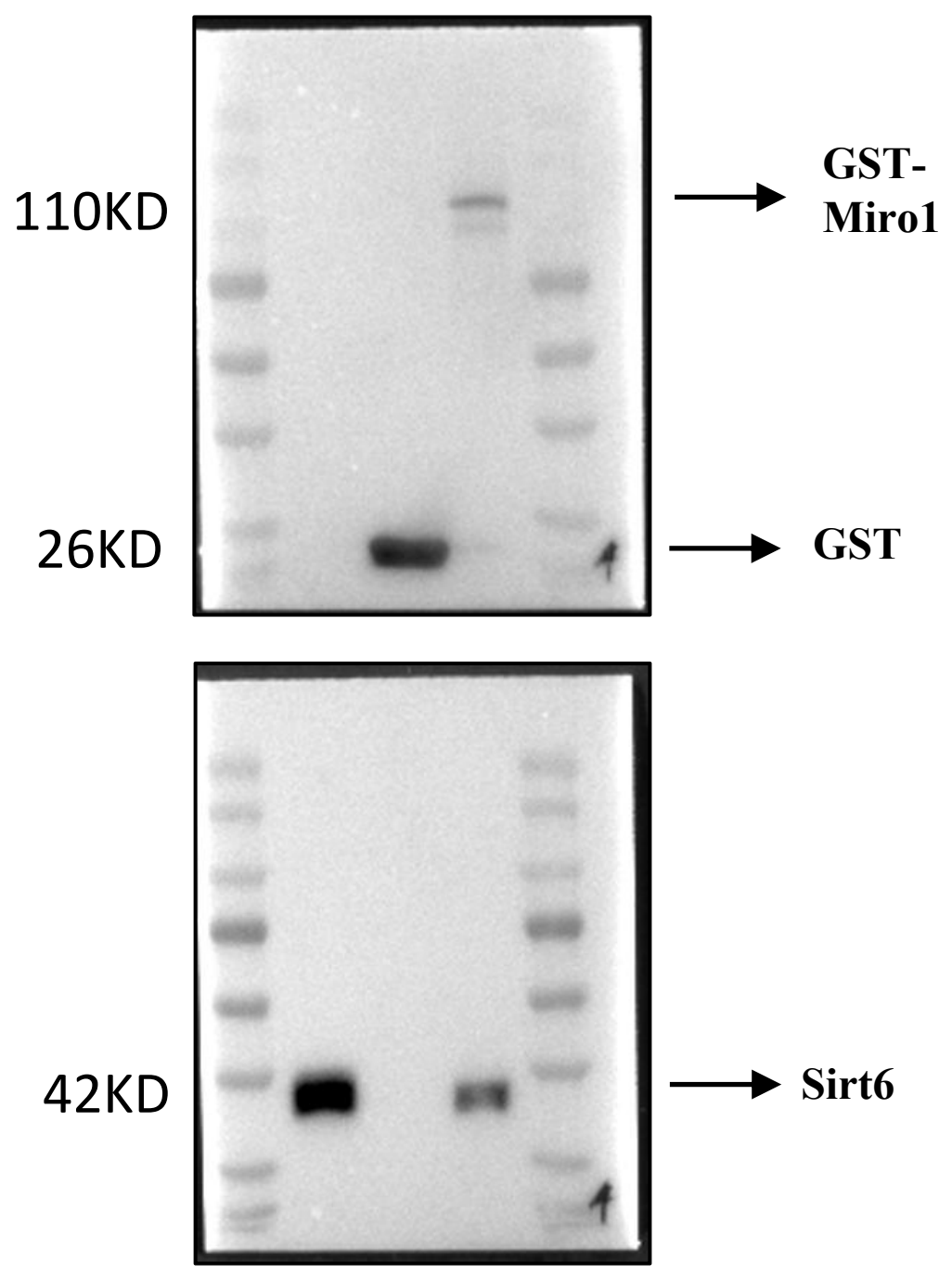

Figure 7B

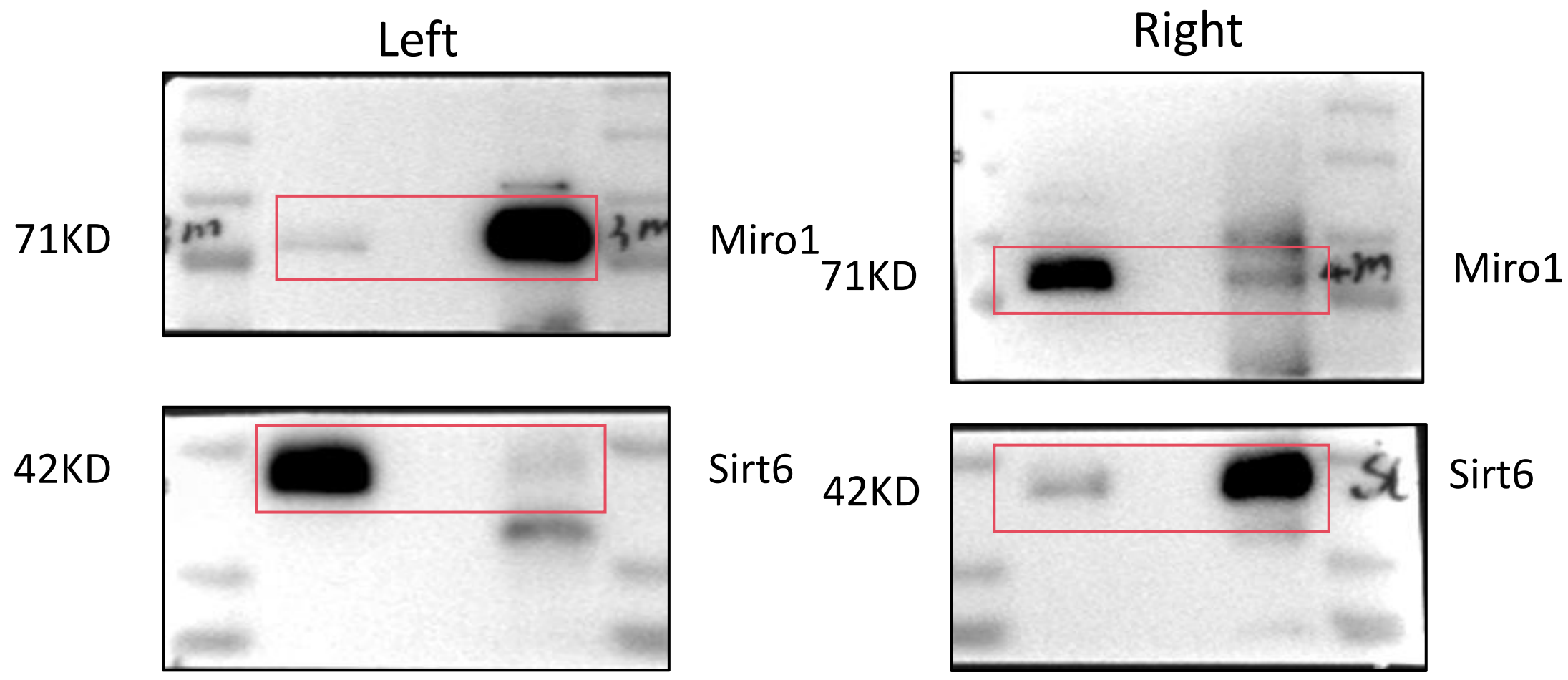

Figure 7C

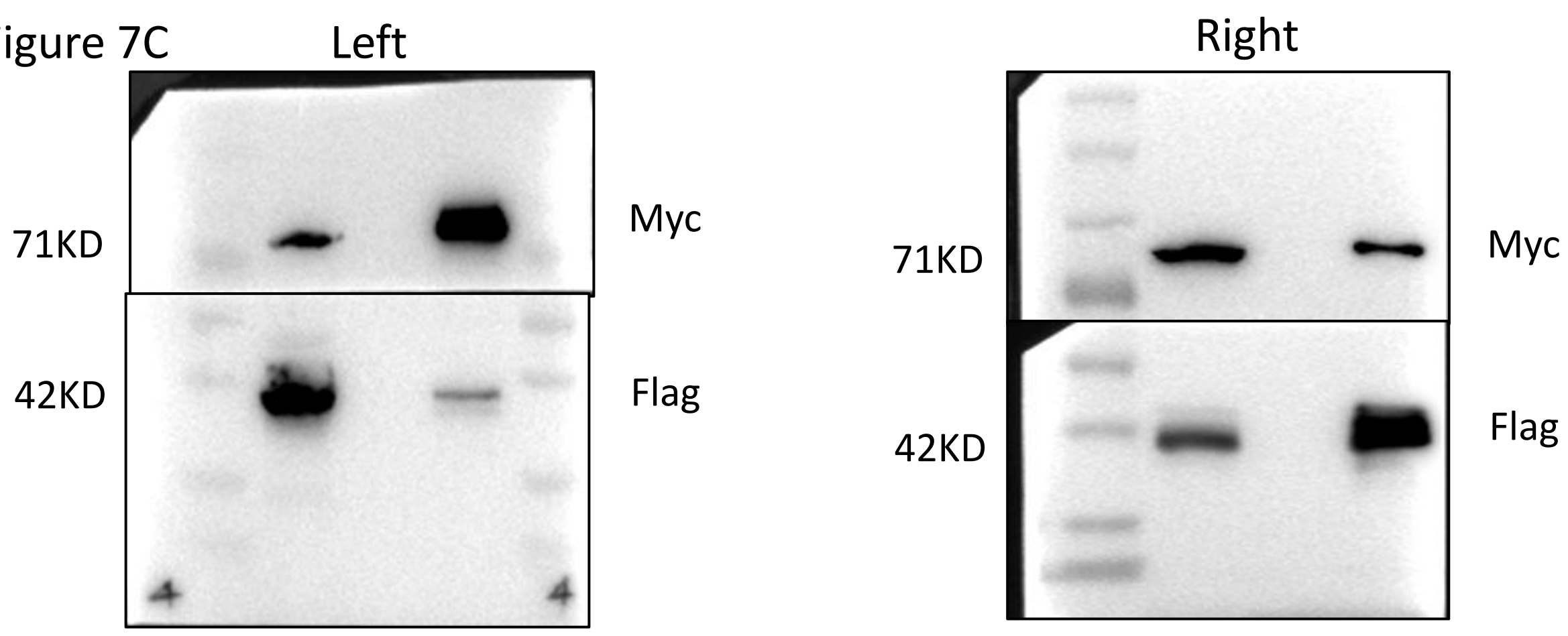

Figure 7E

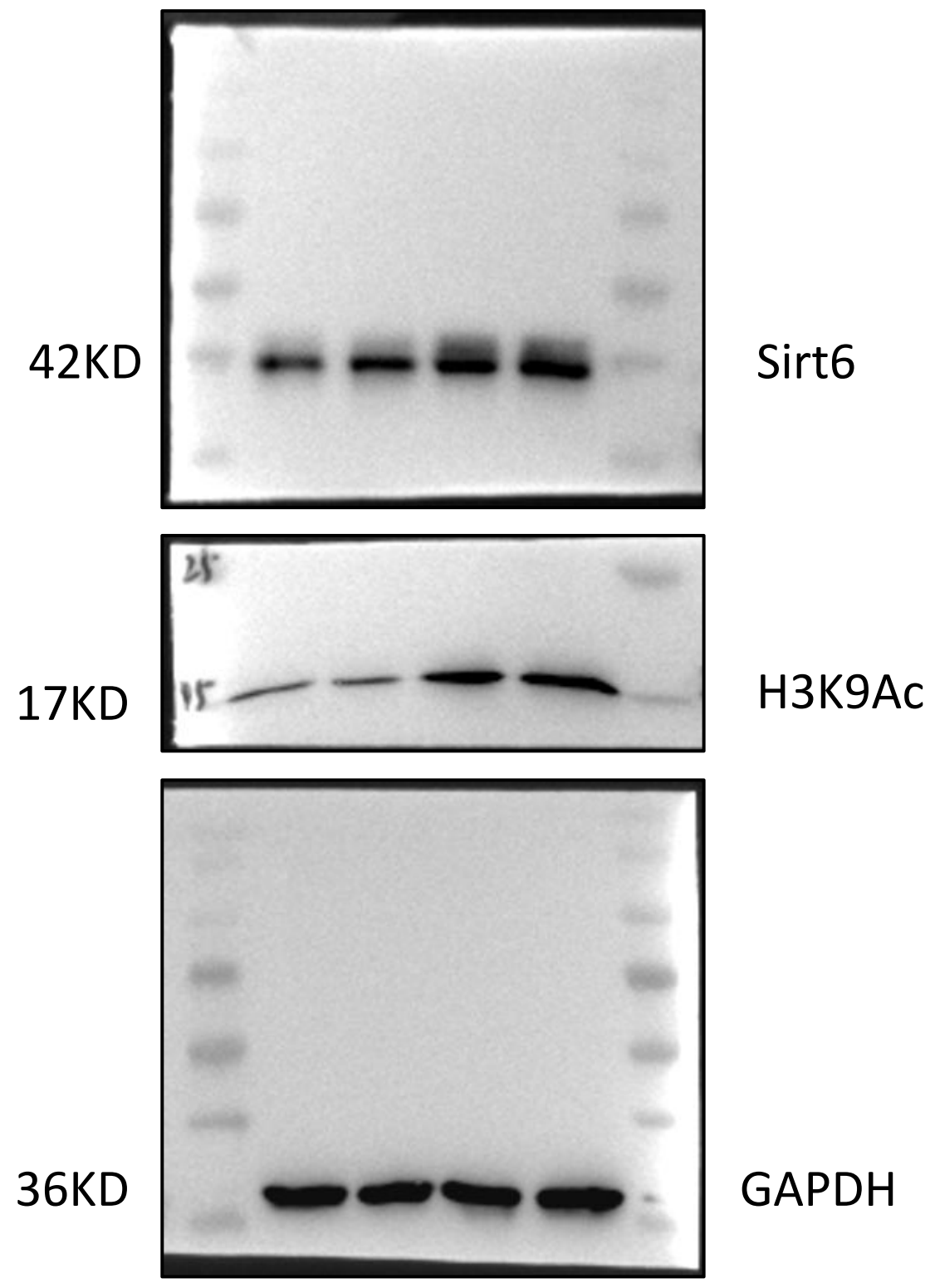

Figure 7F

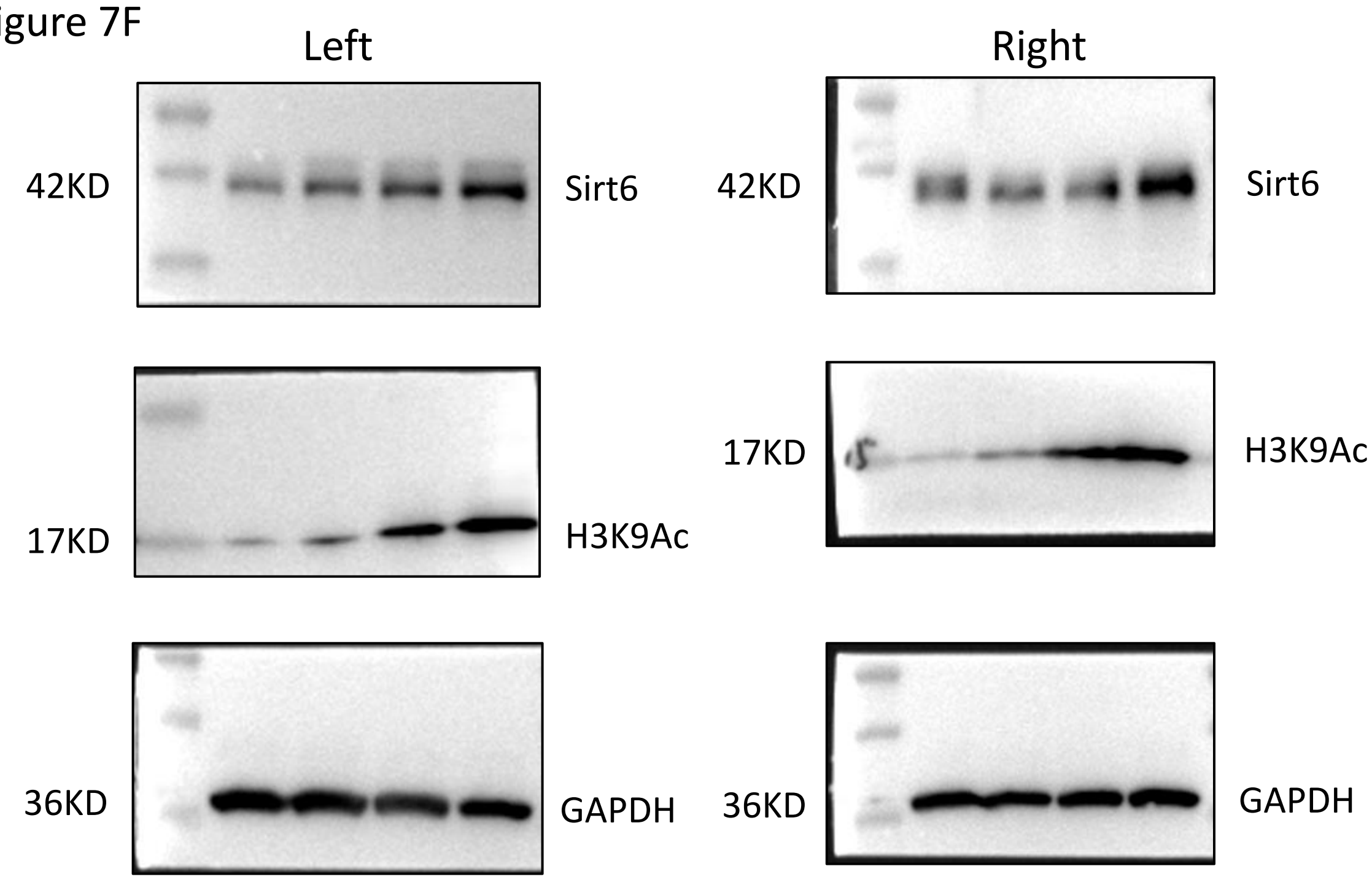

Figure 7G

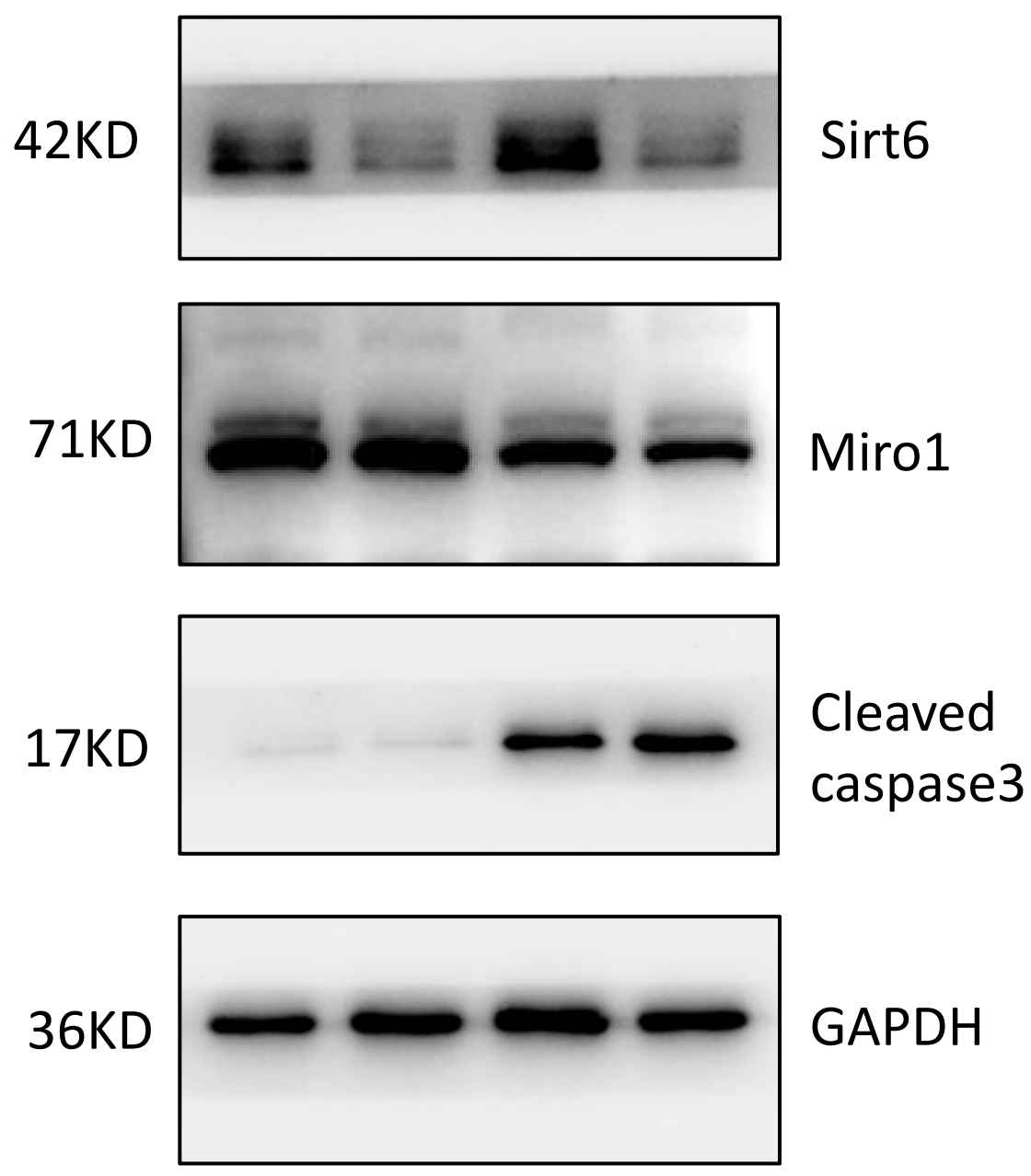

Figure 7H

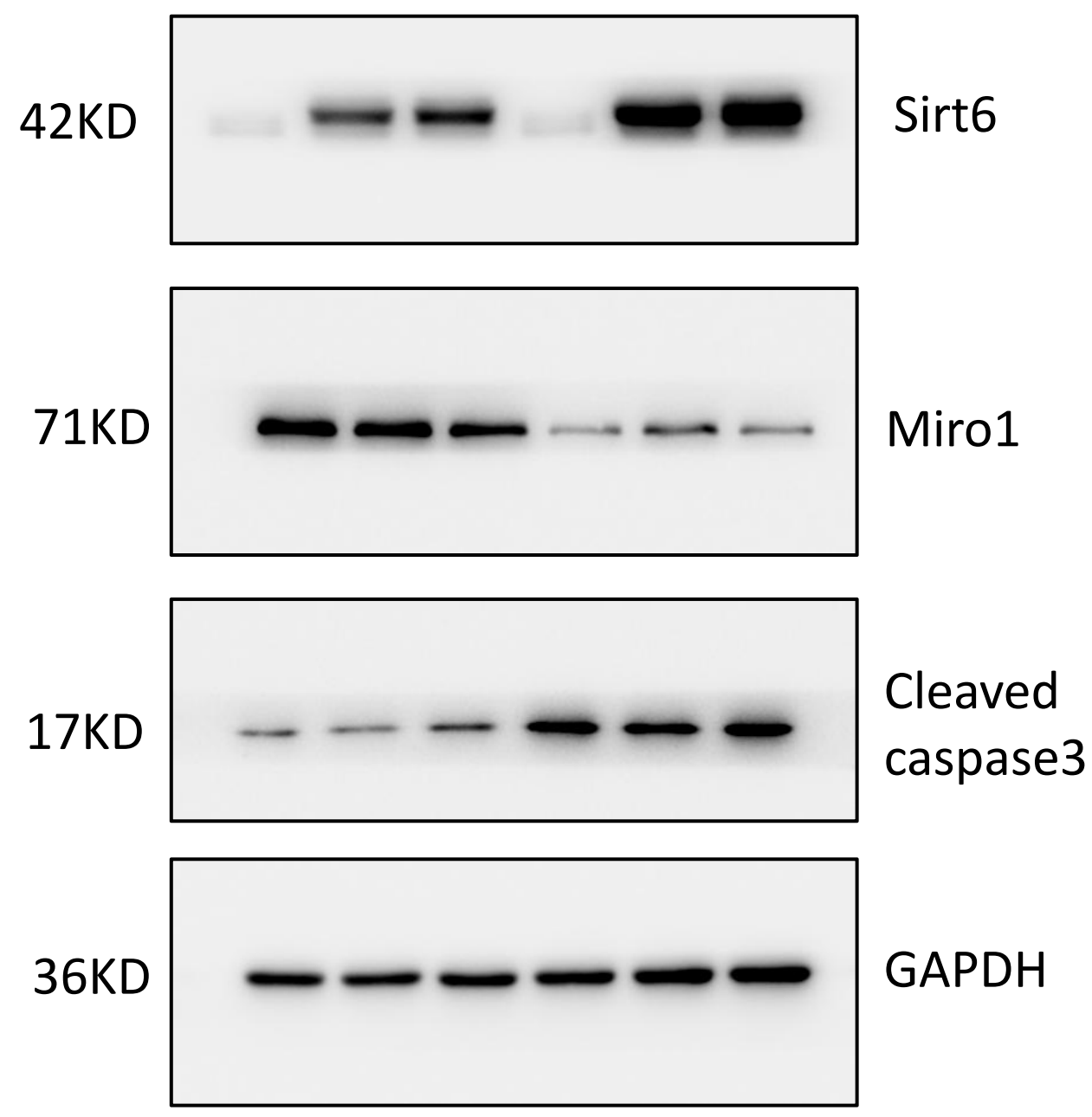

Figure 7I

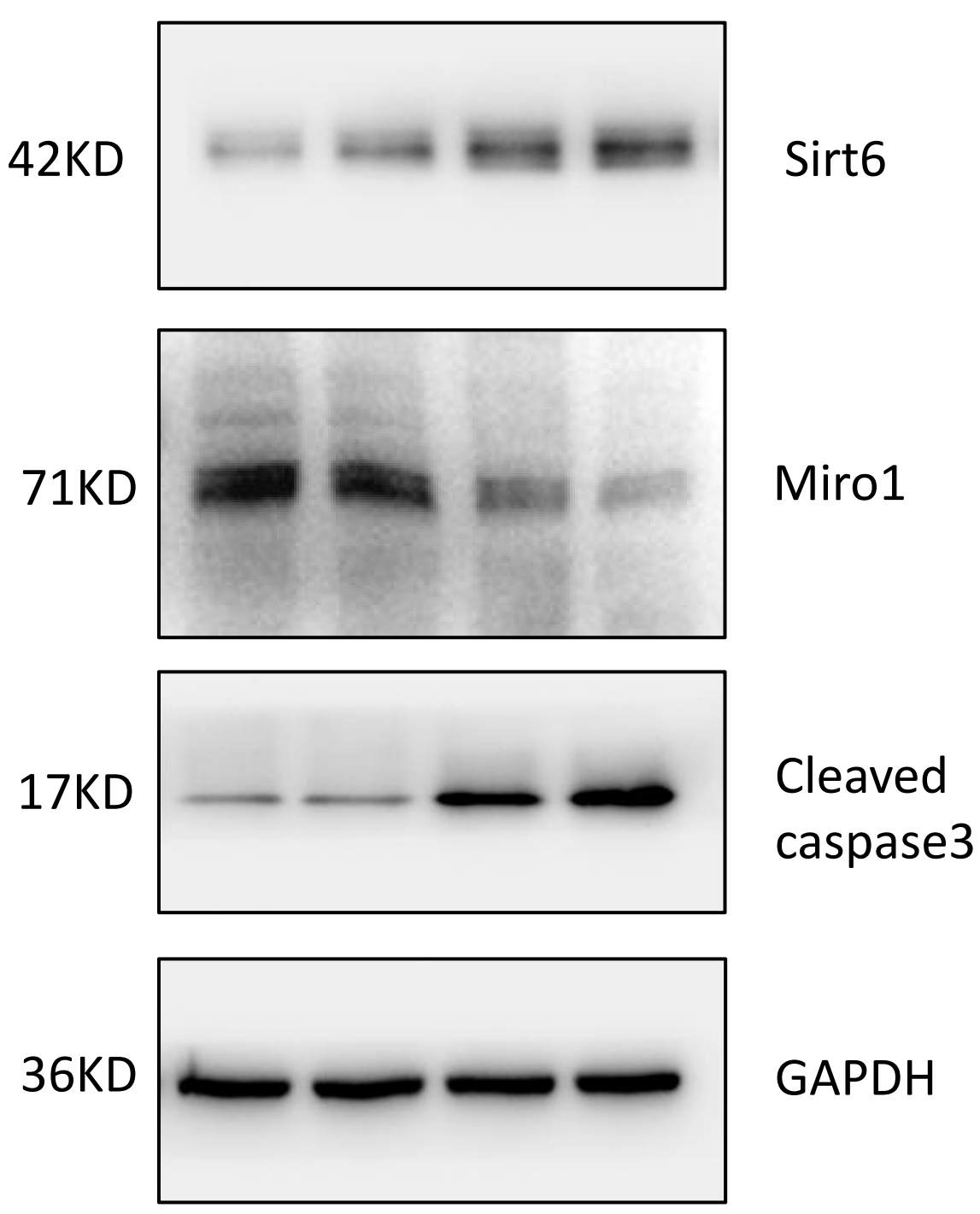

Figure 7J

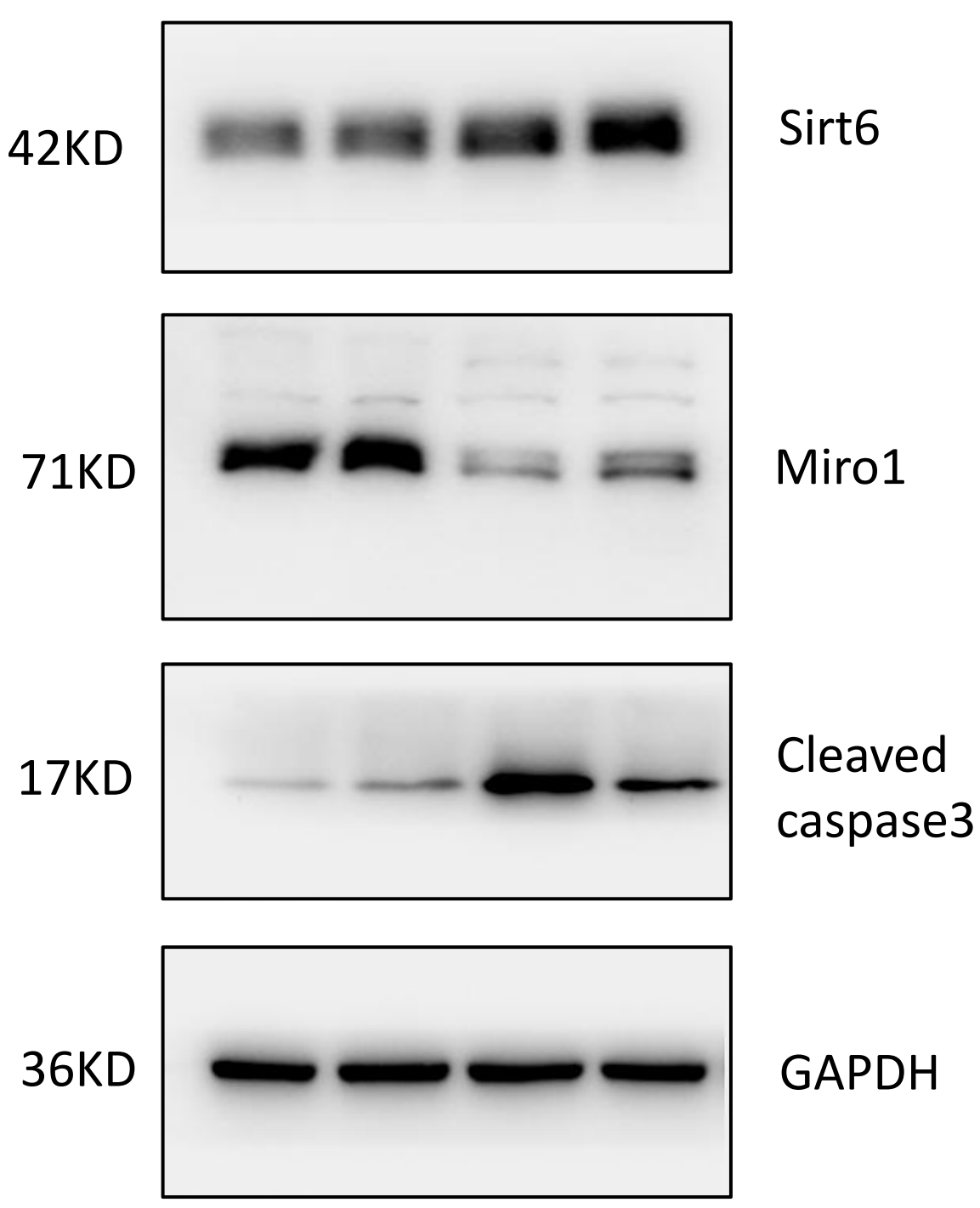

Figure 8A

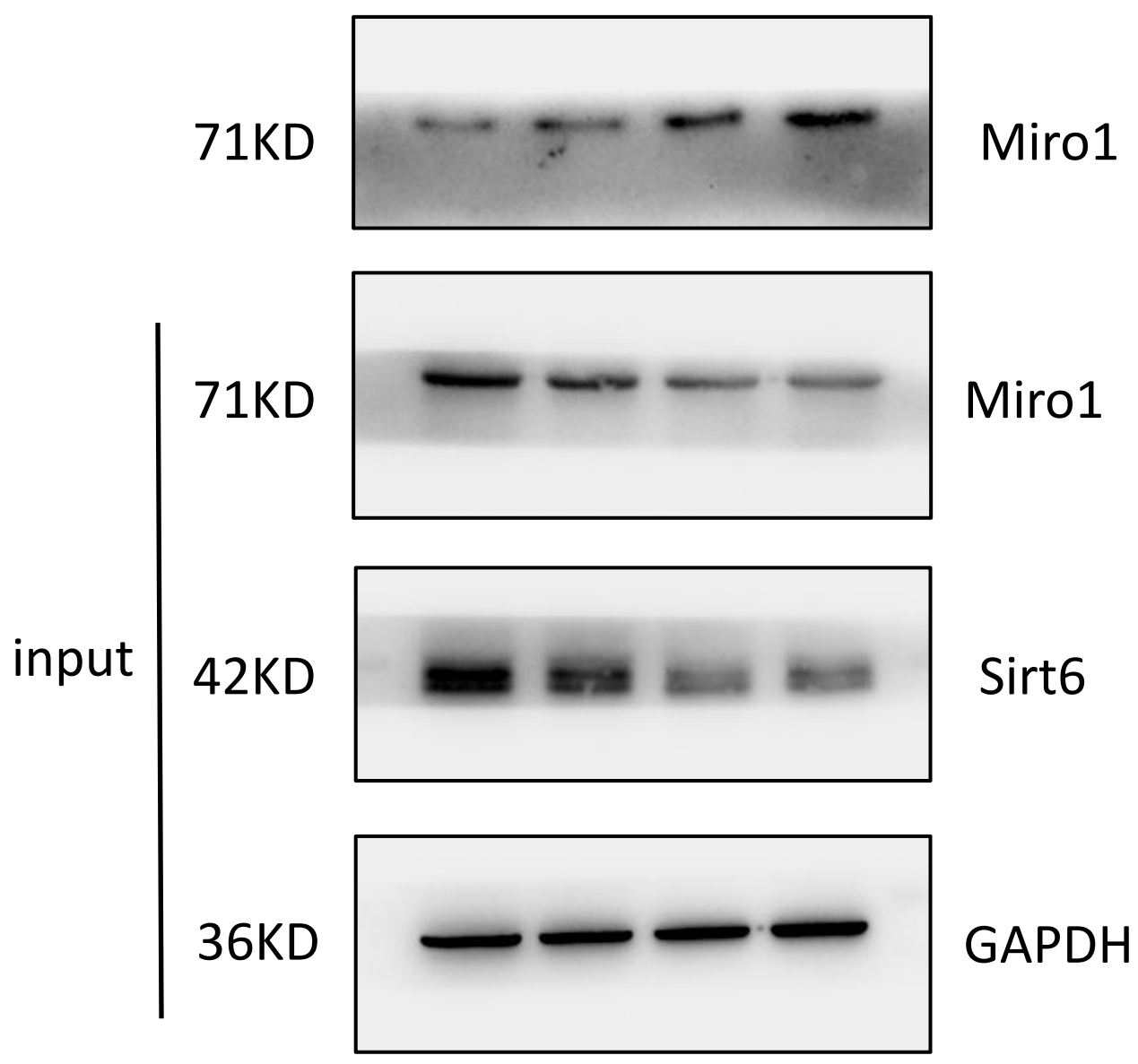

Figure 8B

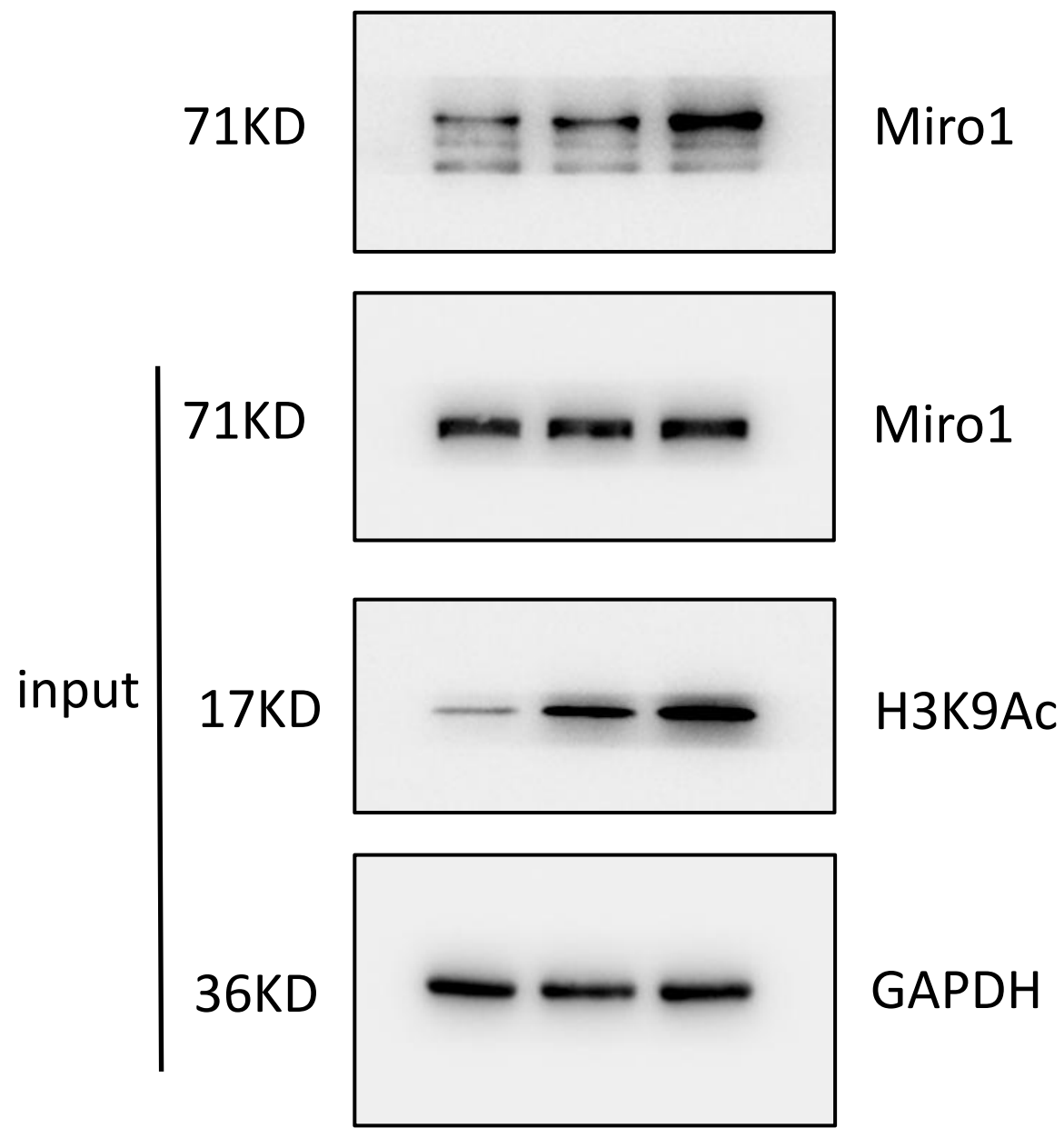

Figure 8C

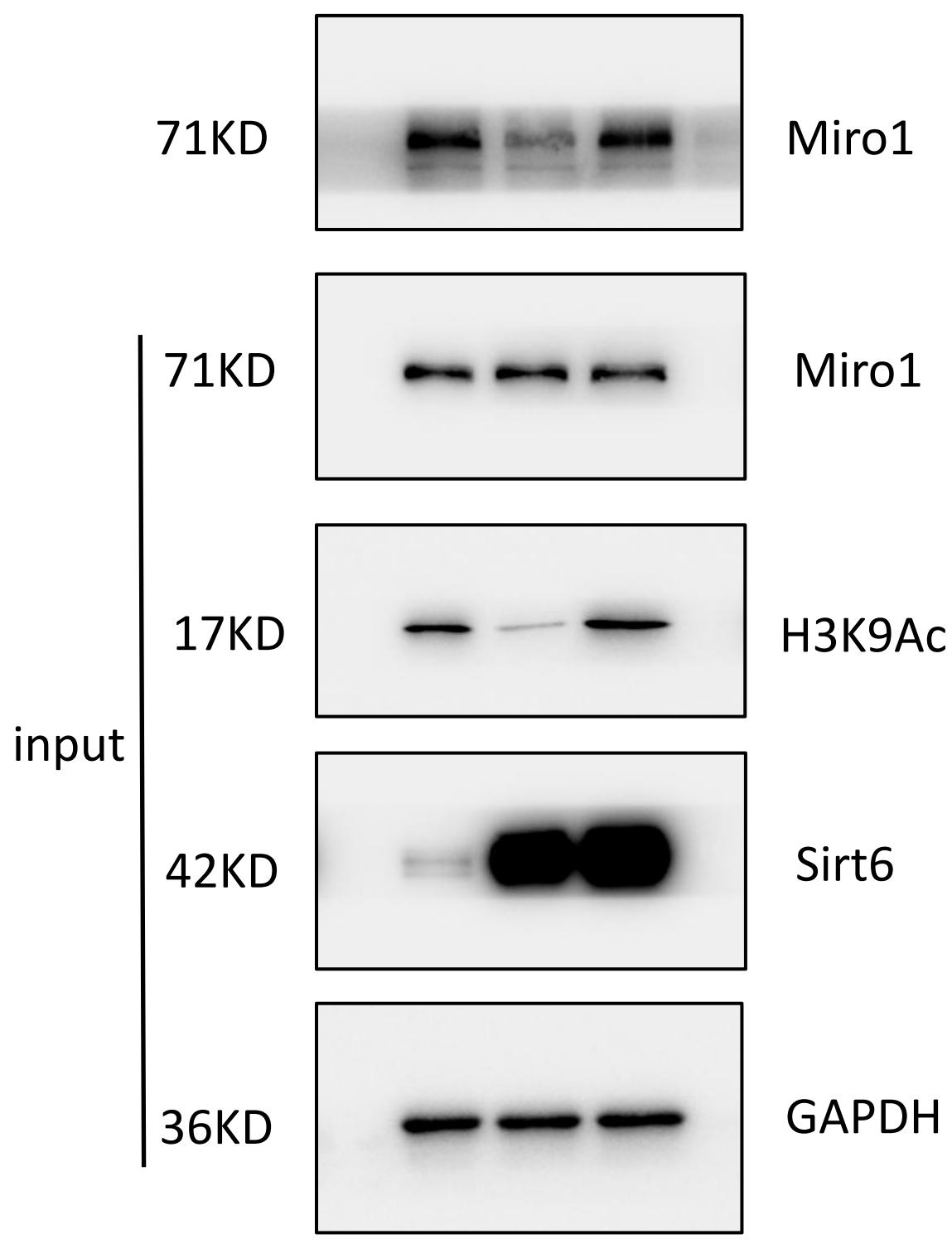

Figure 8D

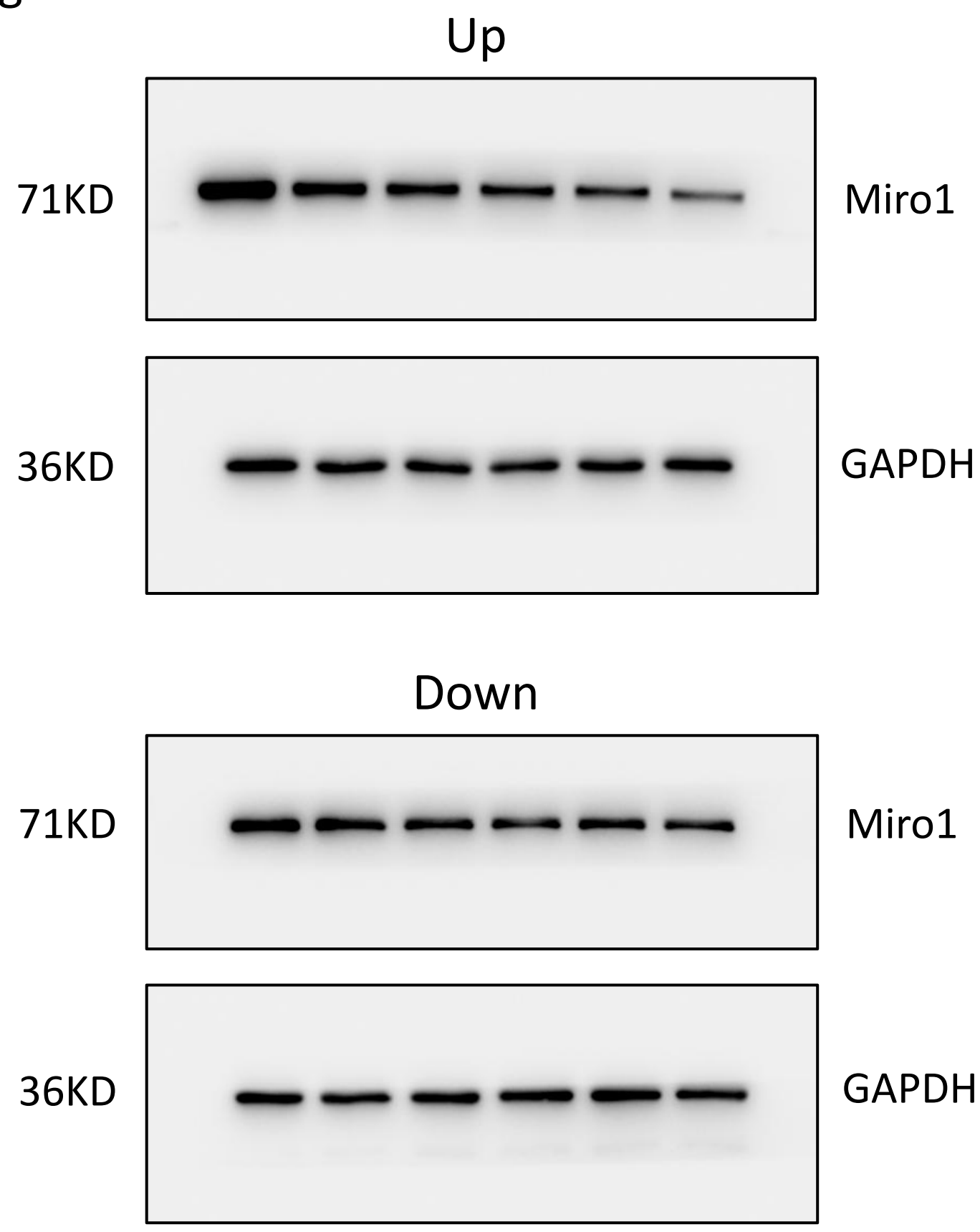

Figure 8E

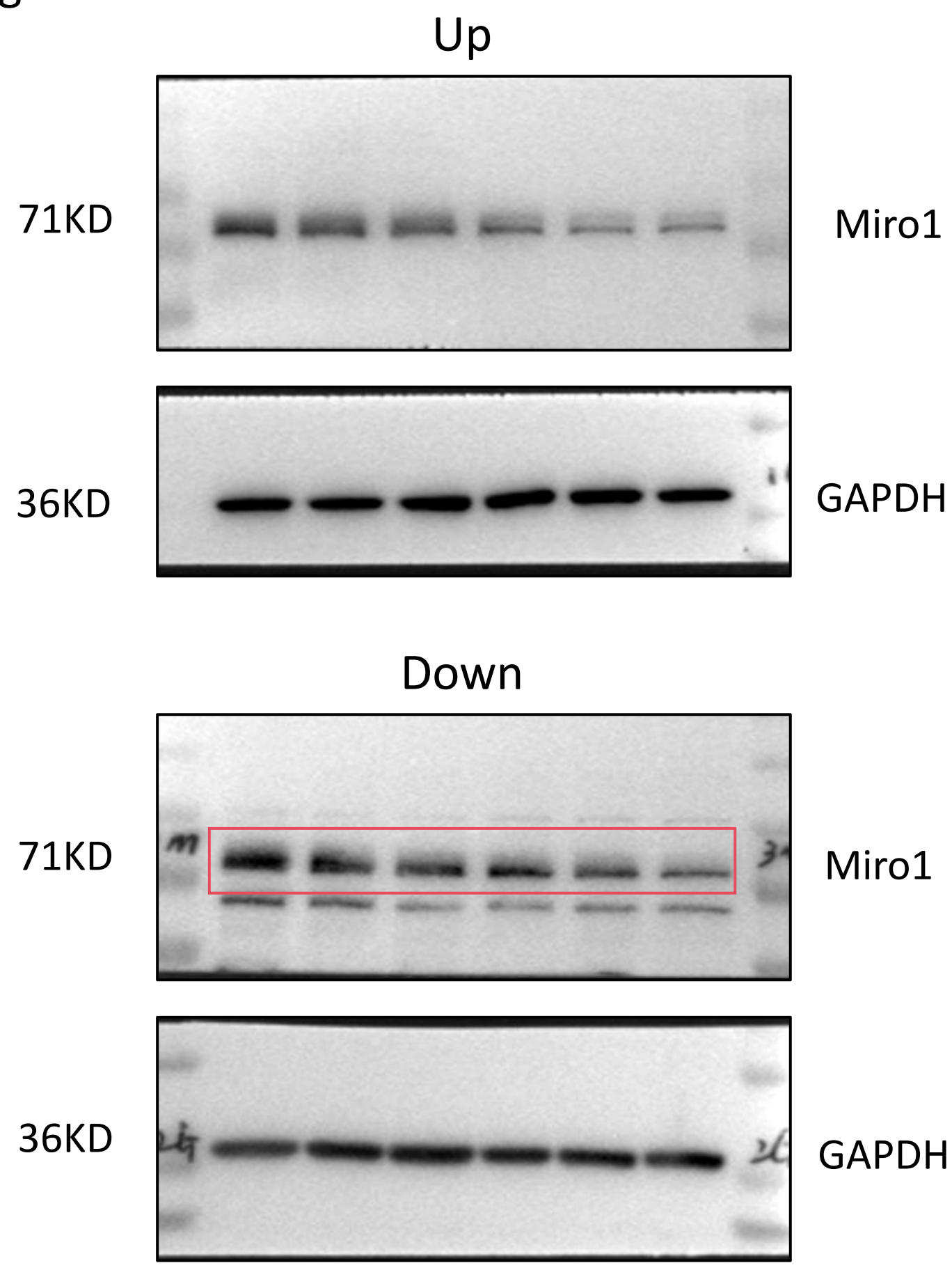

Figure 8F

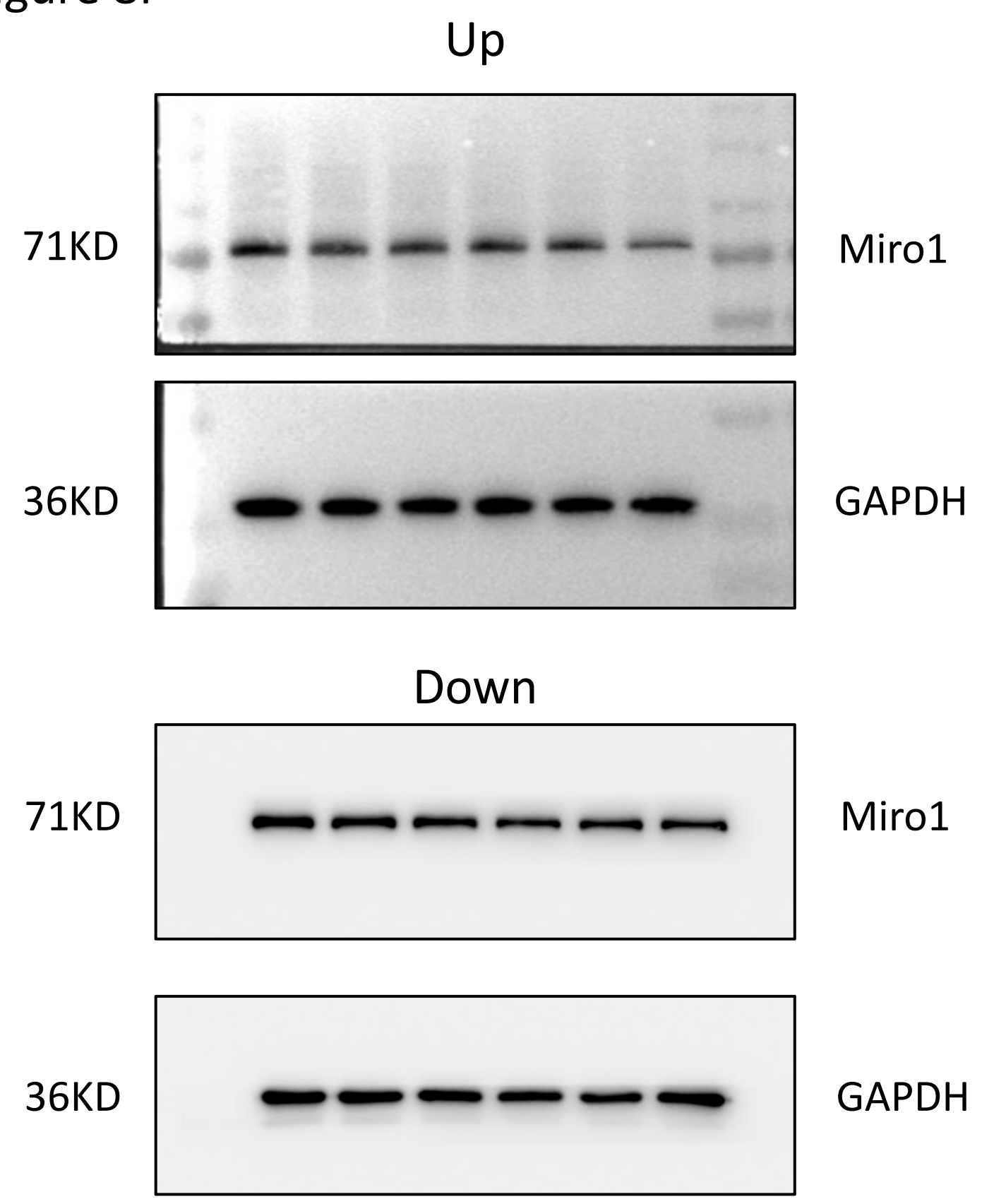

Figure 8G

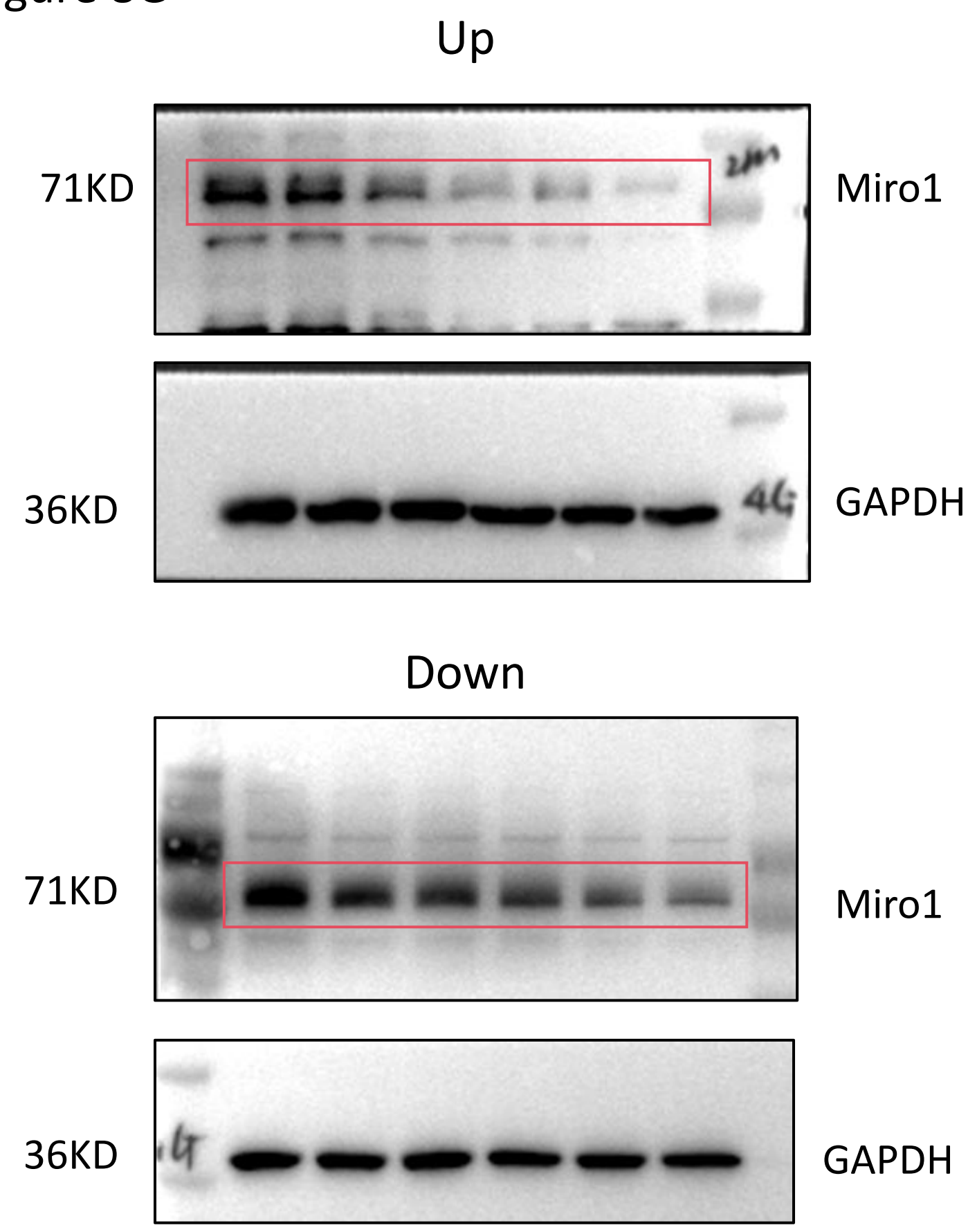

Figure 8H

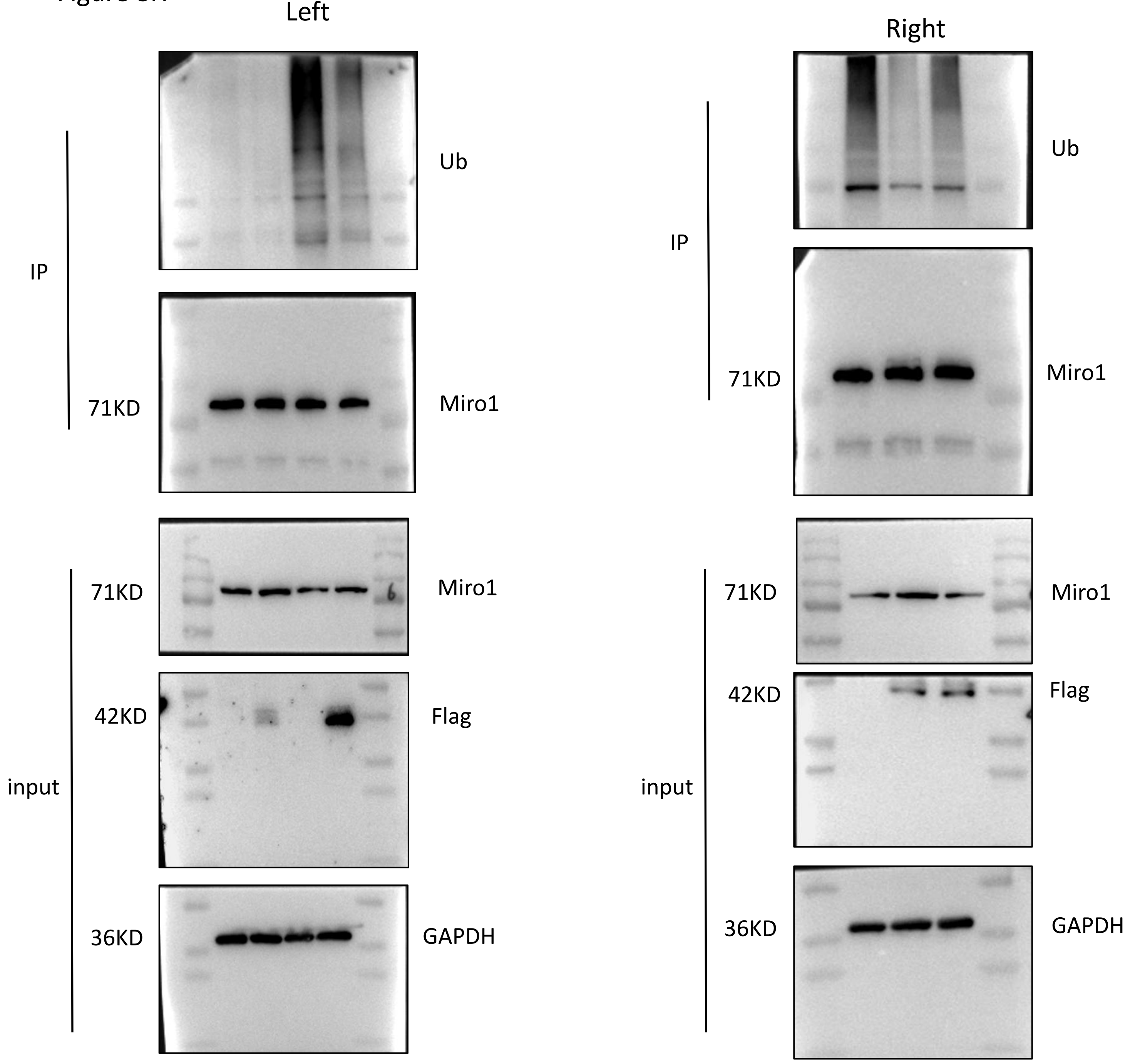

Figure 8I

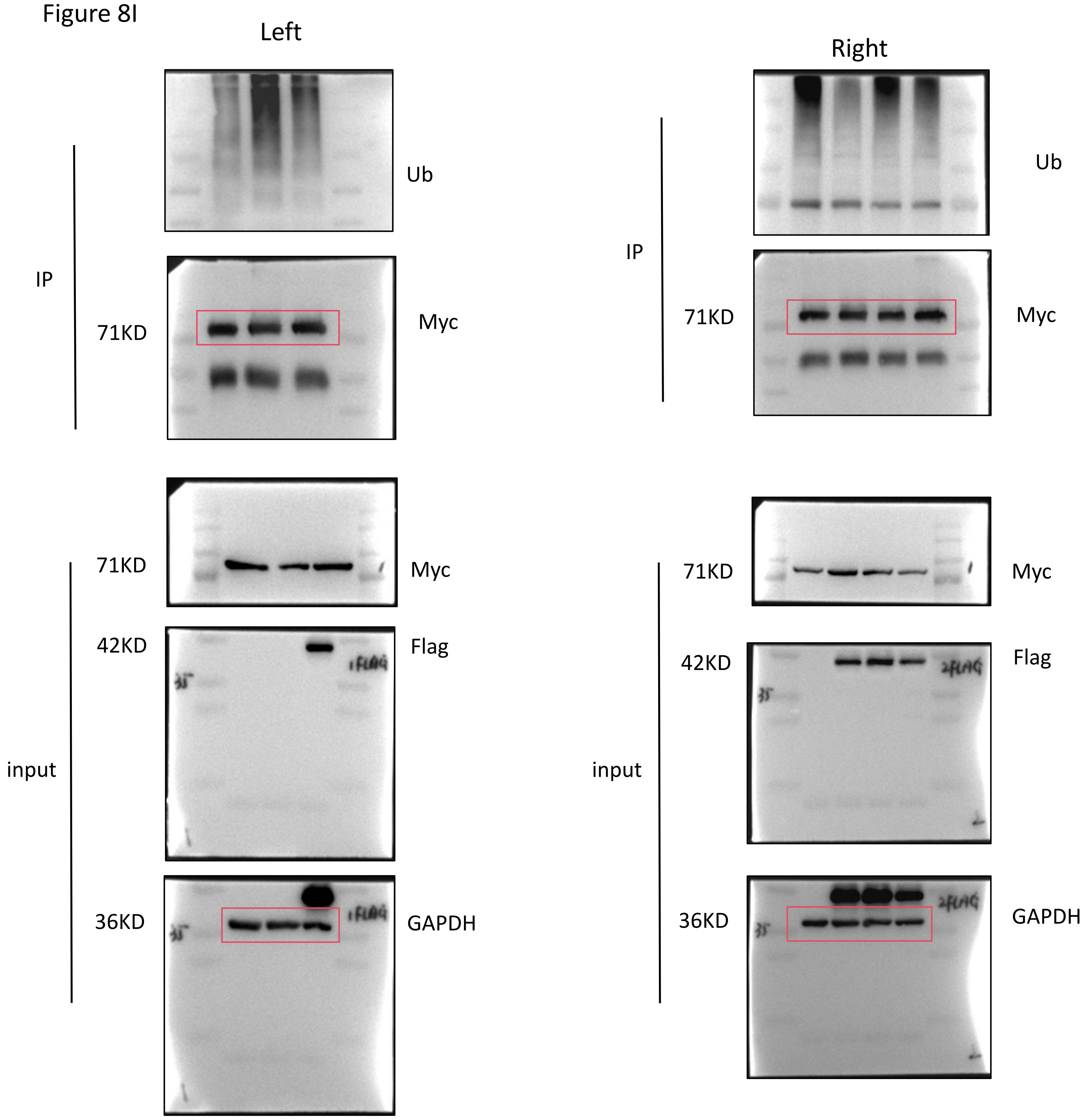

Figure 9A

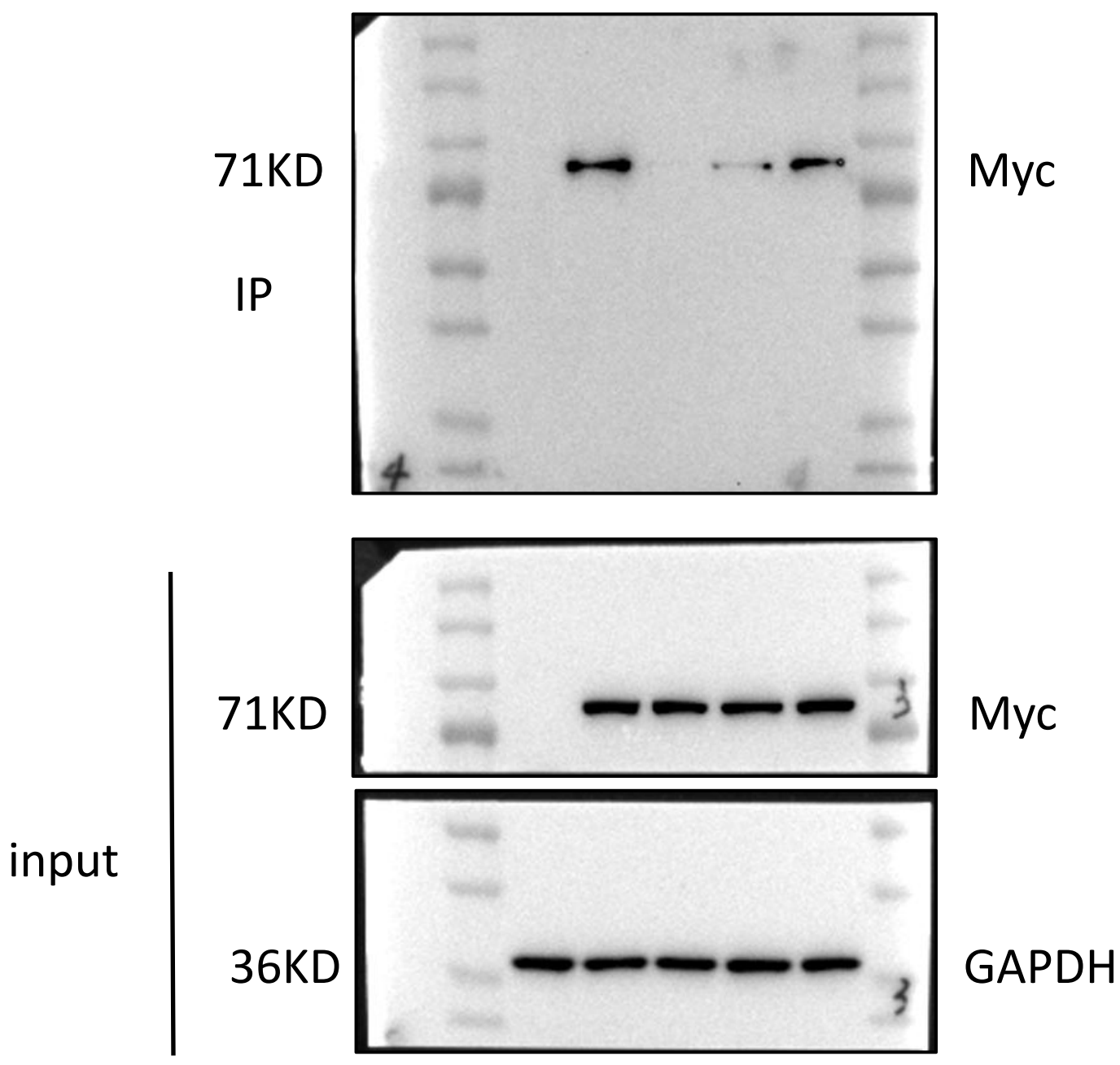

Figure 9B

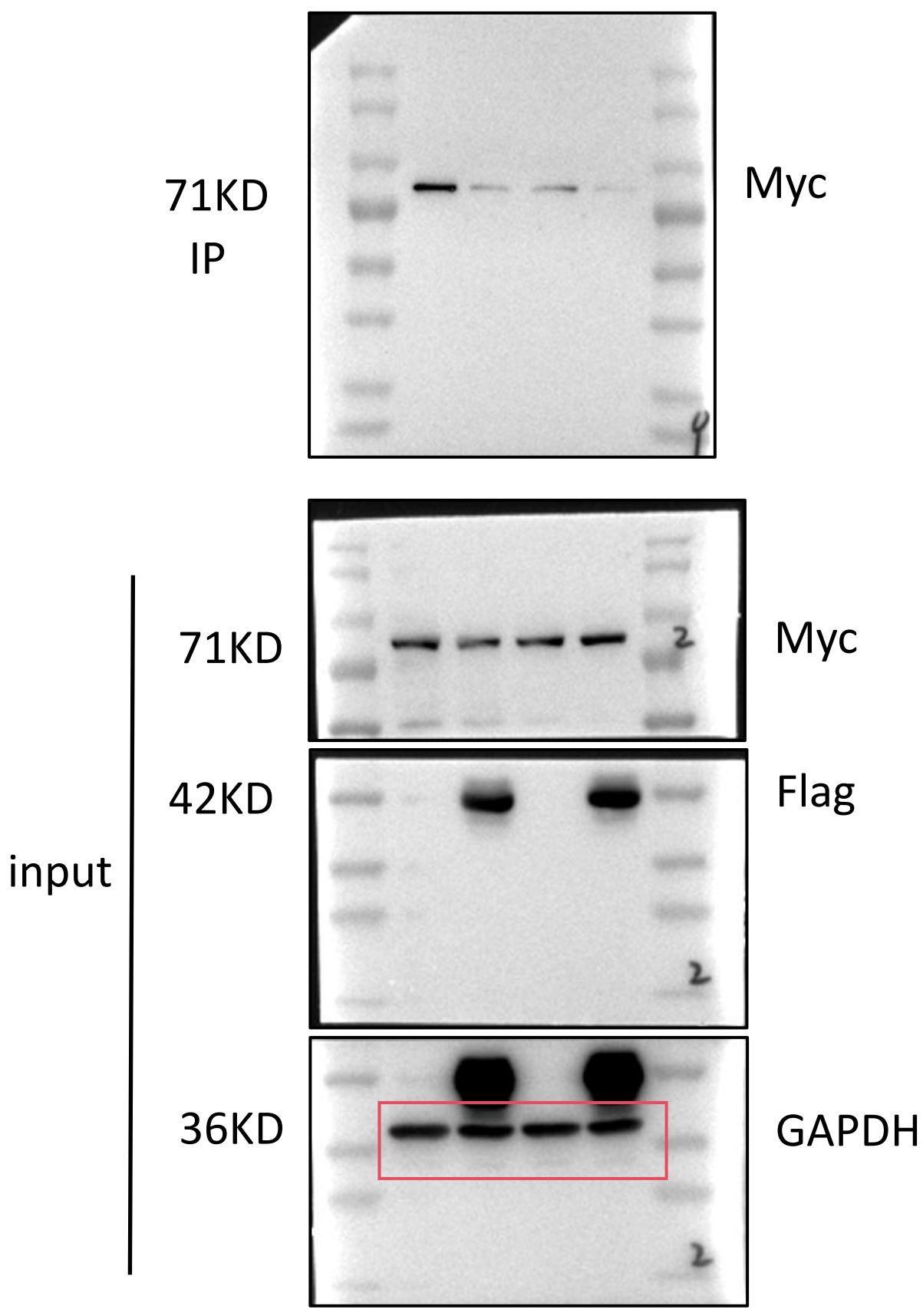

Figure 9C

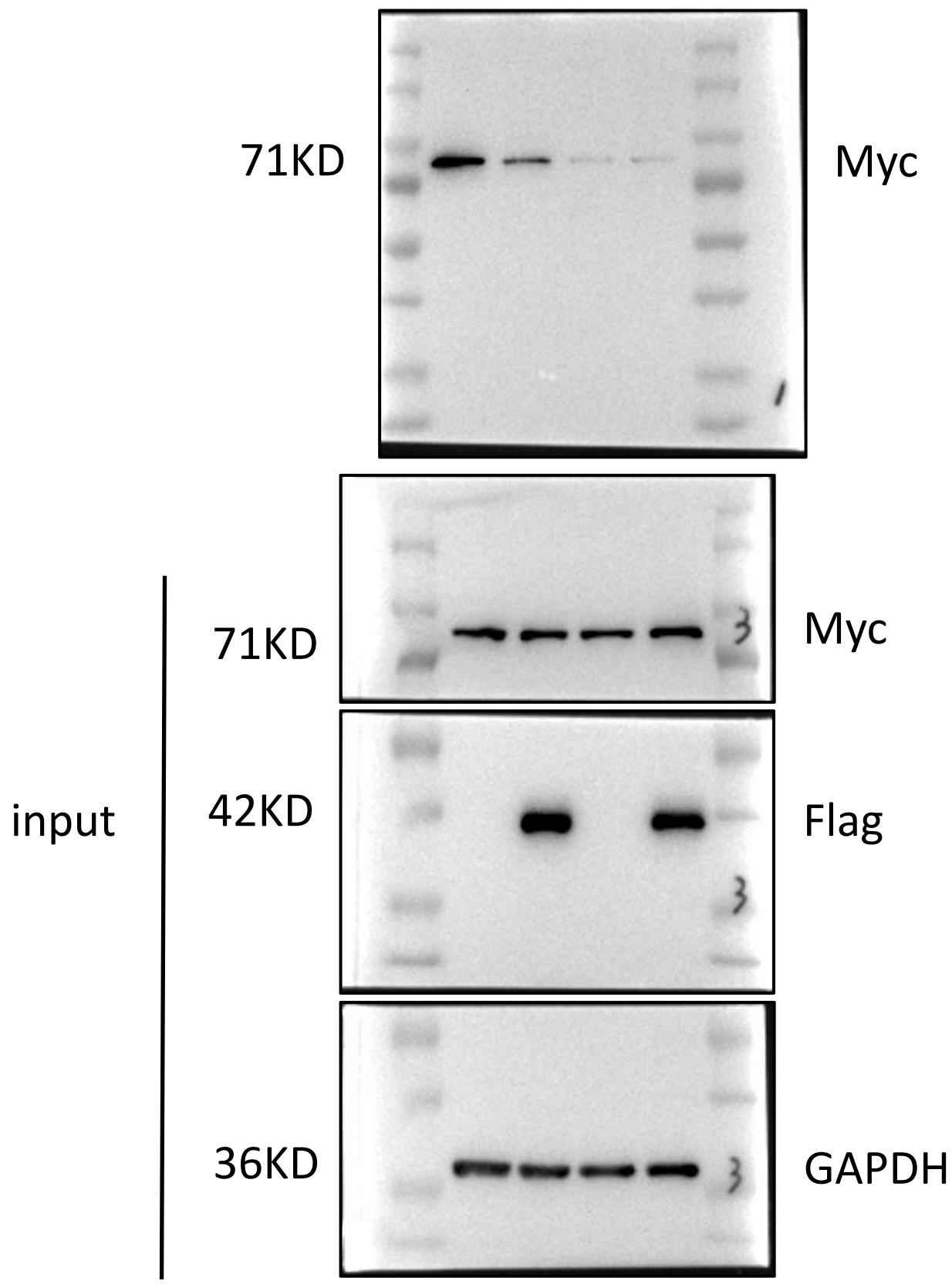

Figure 9G

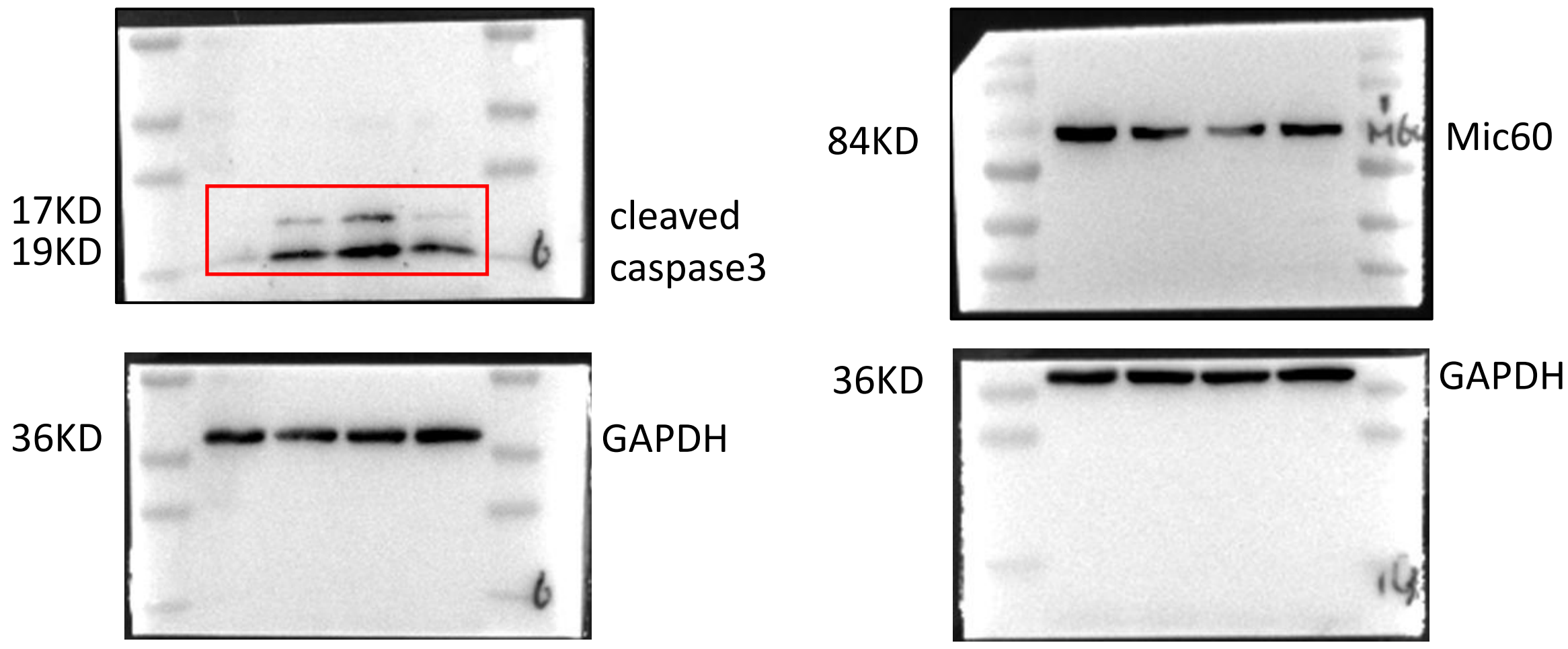

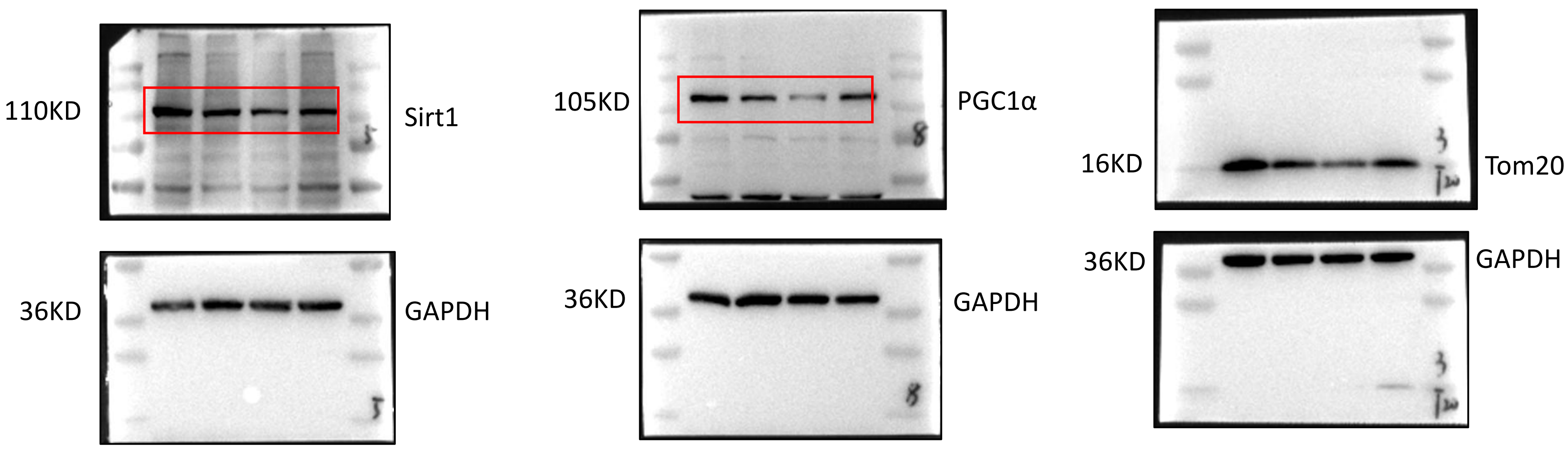

Supplementary Figure 1C

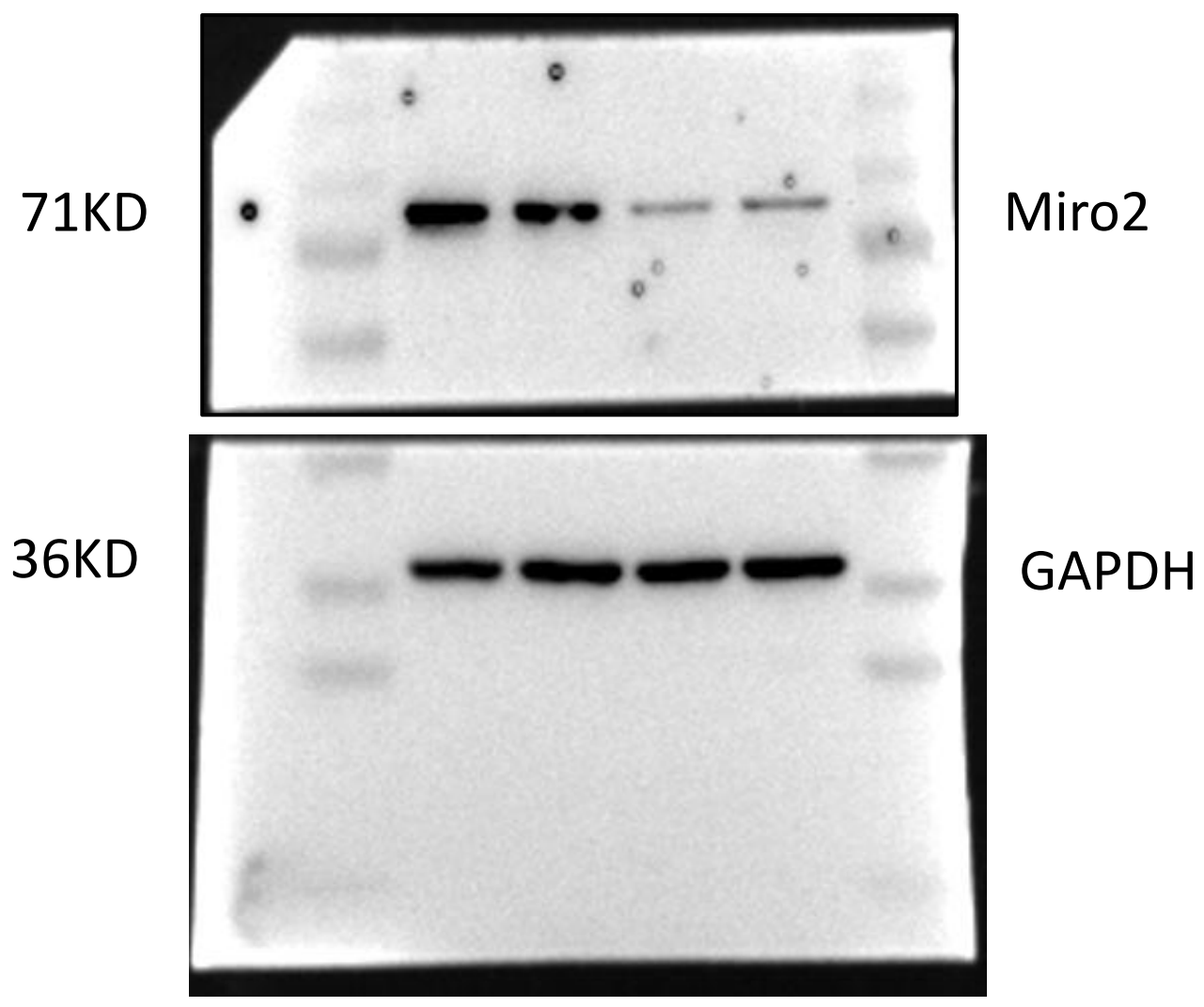

Supplementary Figure 1D

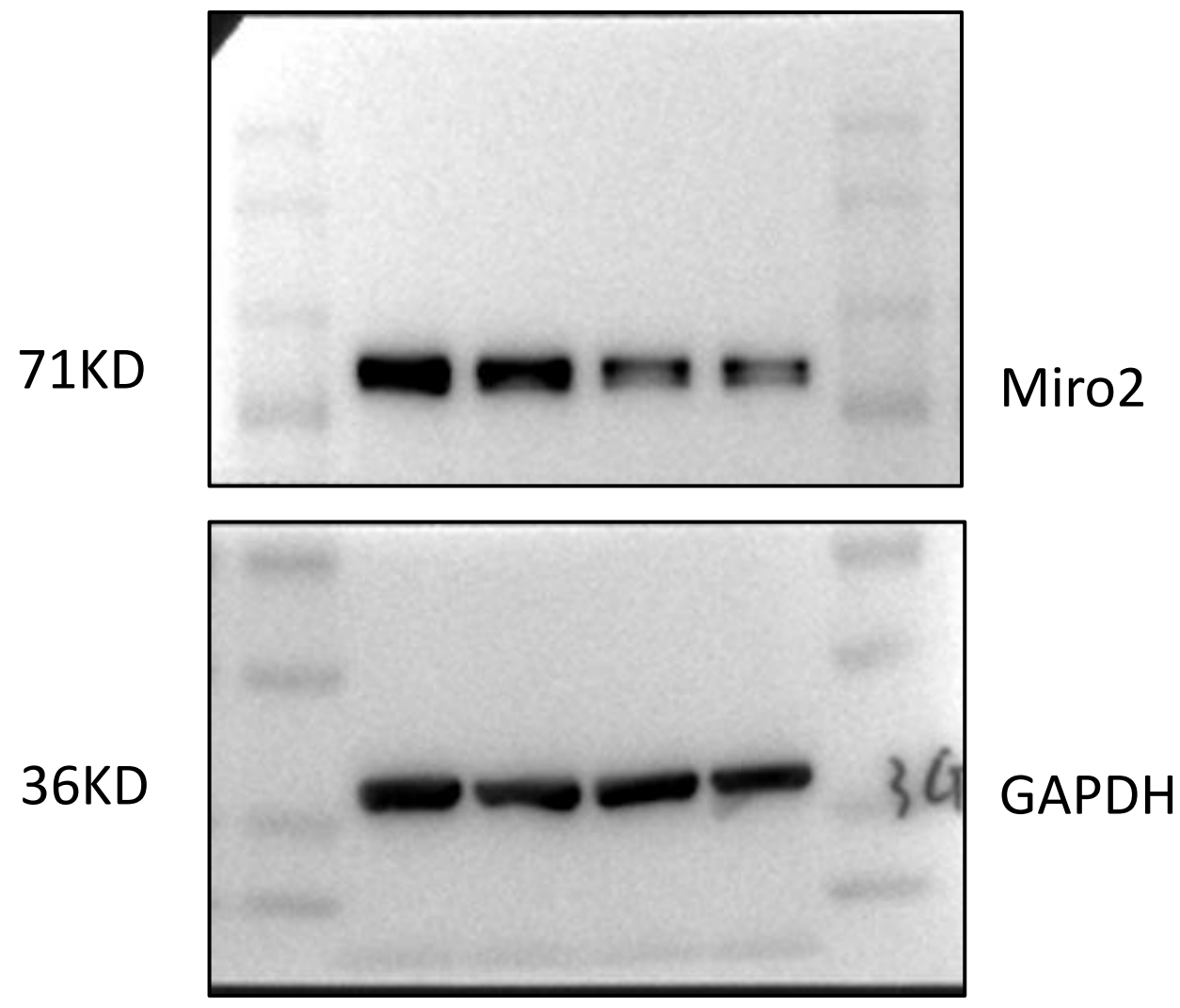

Supplementary Figure 1E

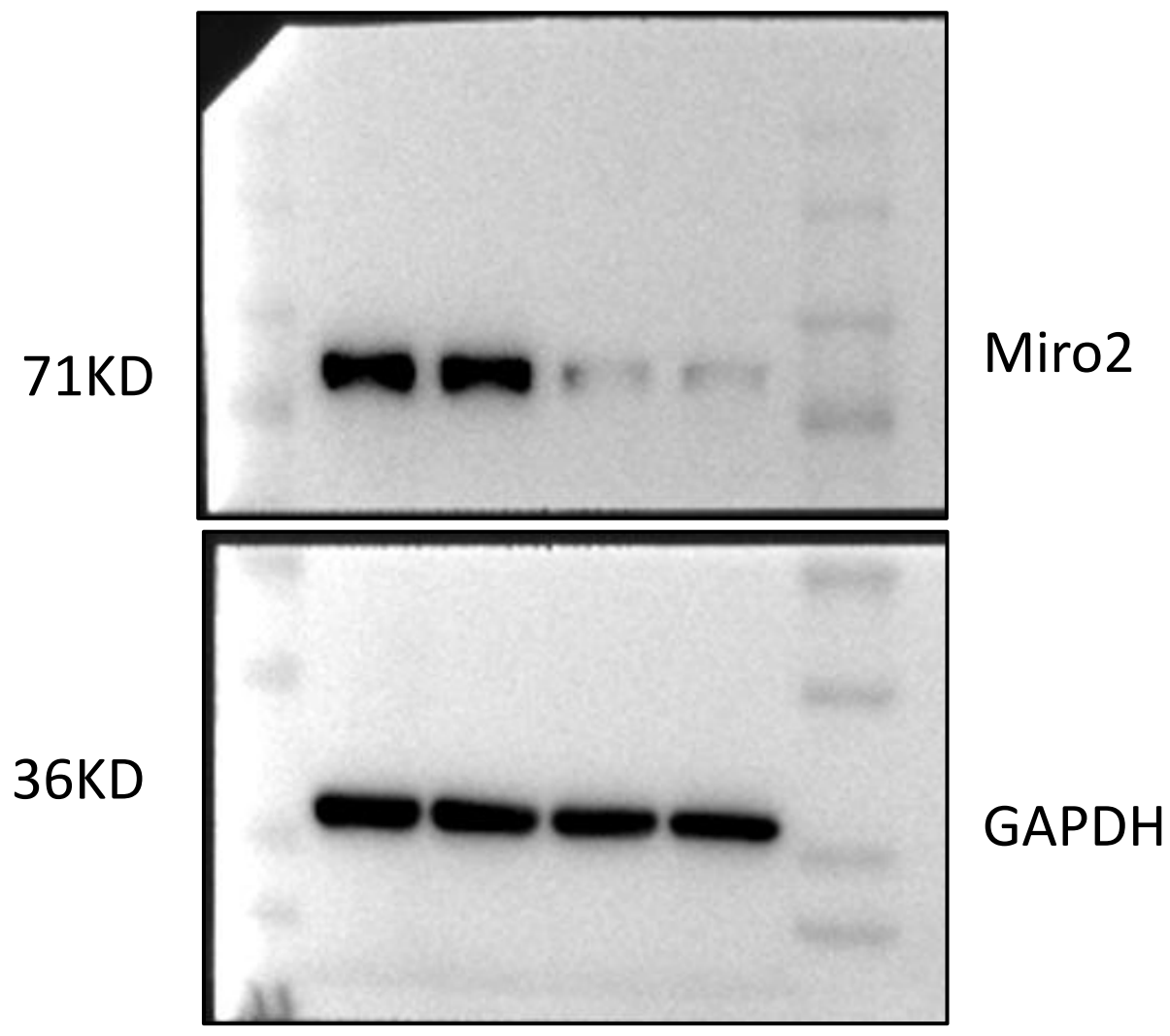

Supplementary Figure 1F

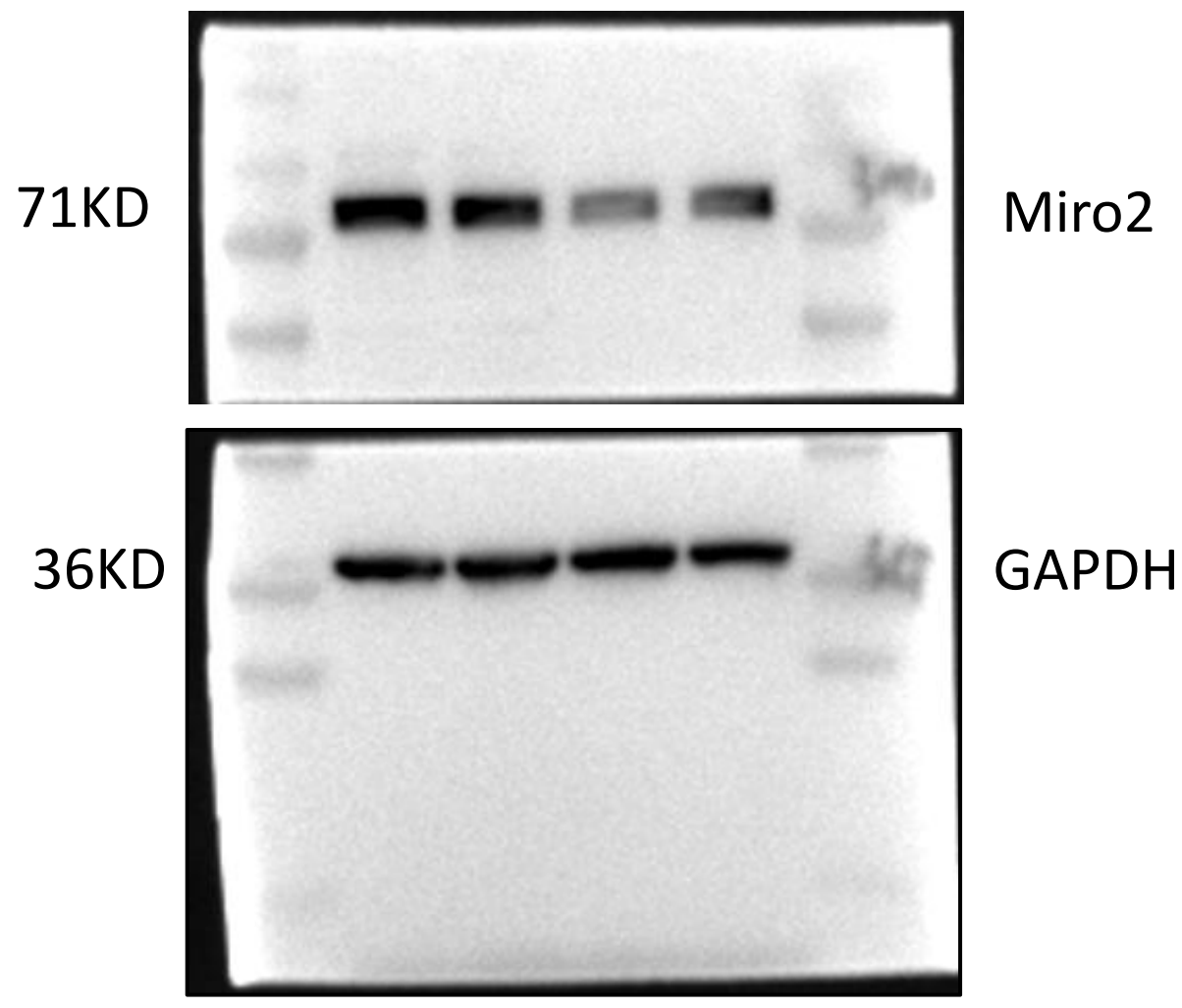

Supplementary Figure 2D

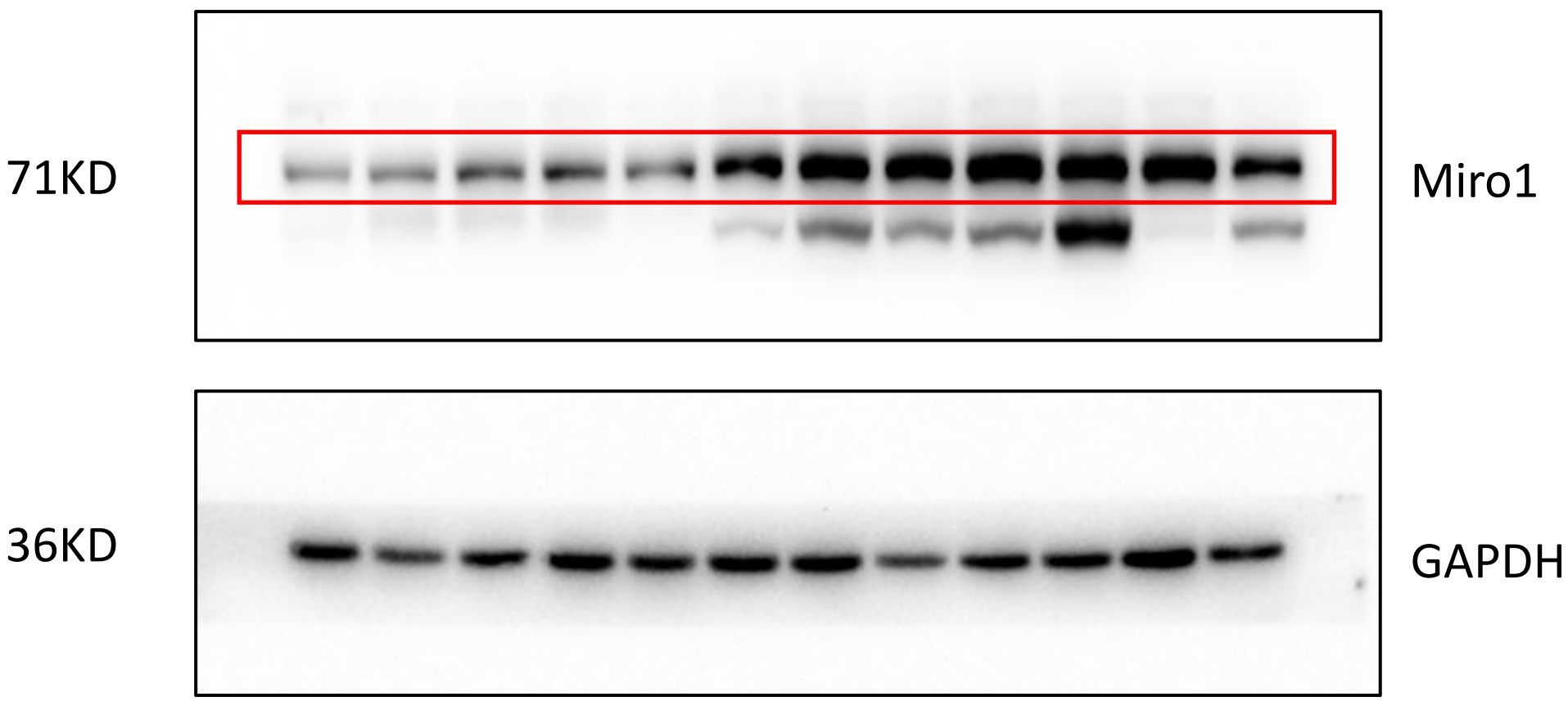

Supplementary Figure 3H

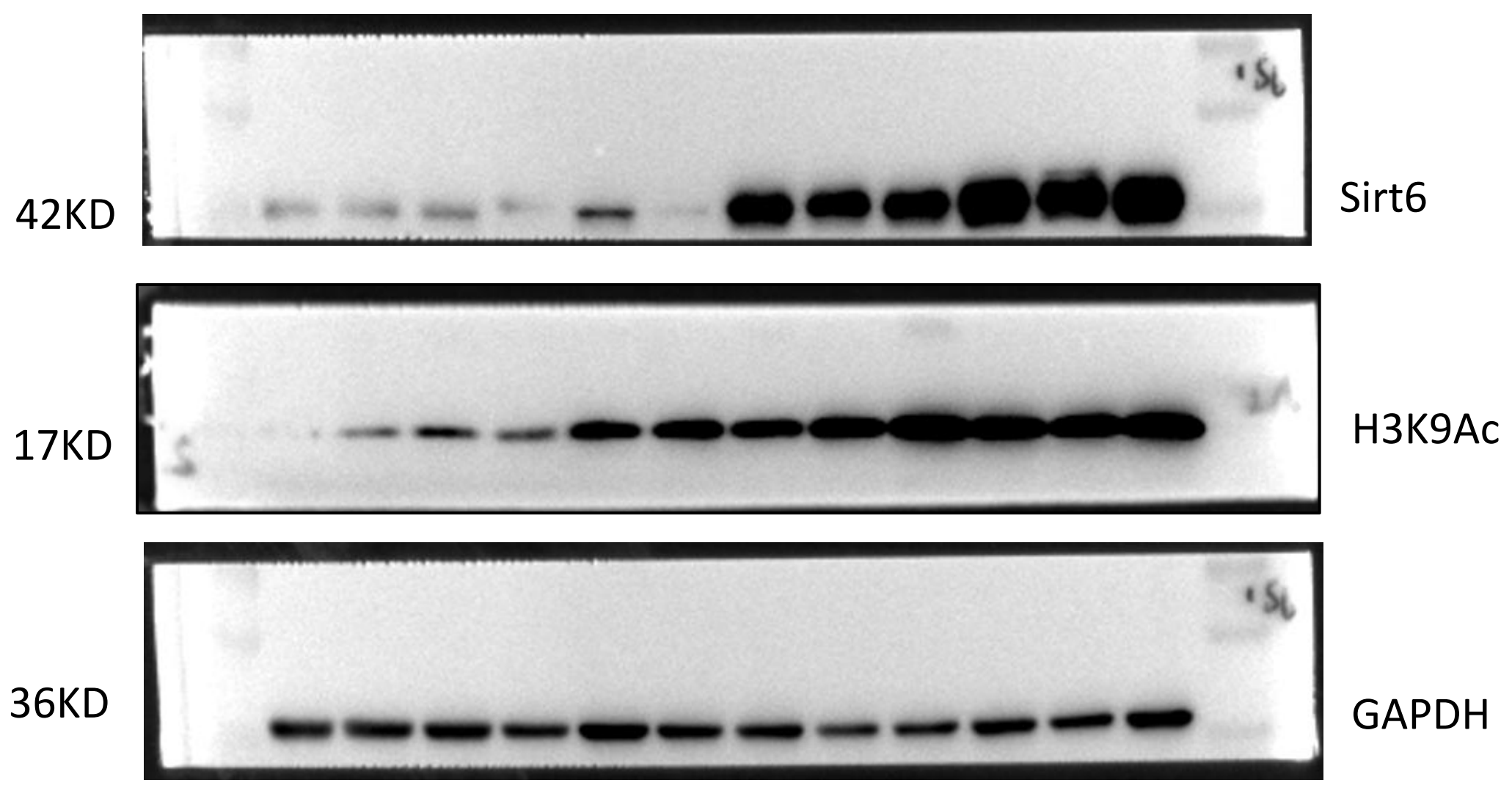

Supplementary Figure 4A

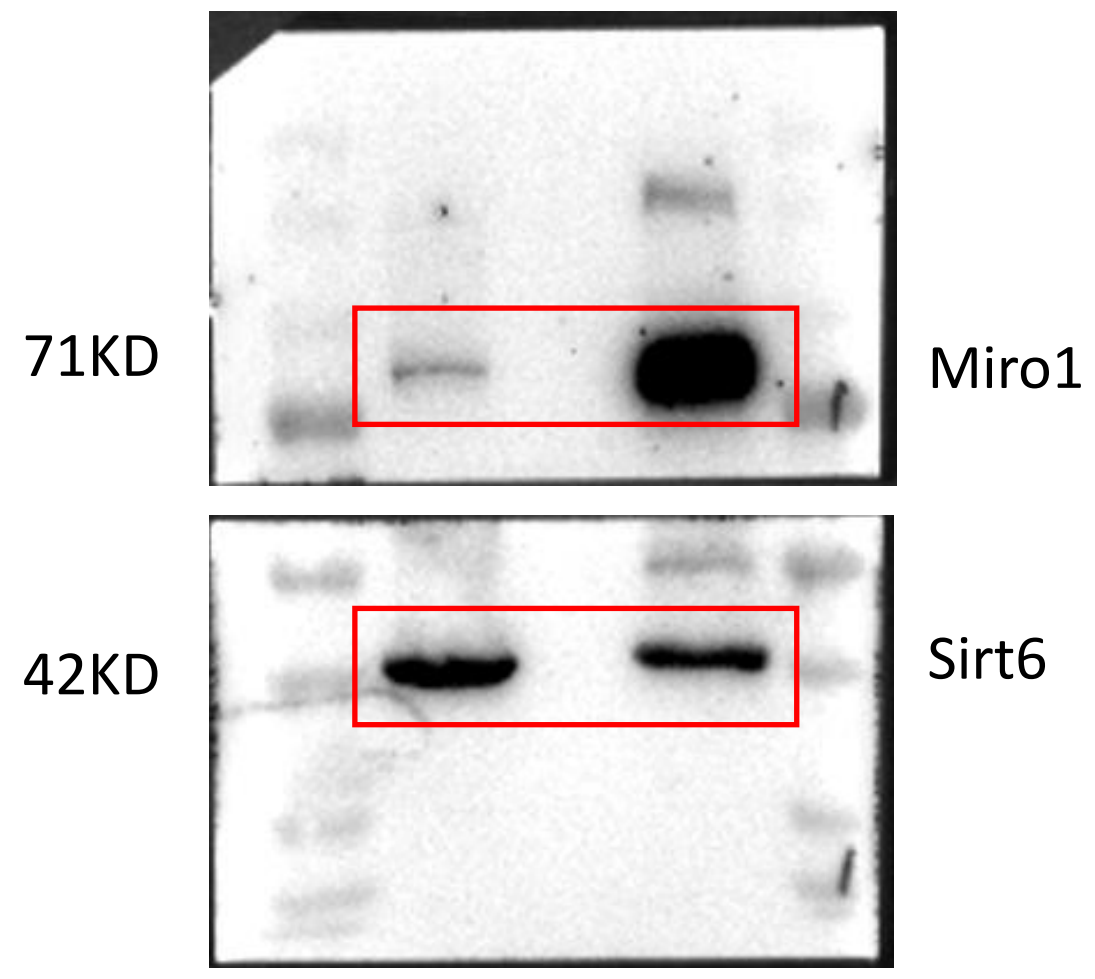

Supplementary Figure 4B

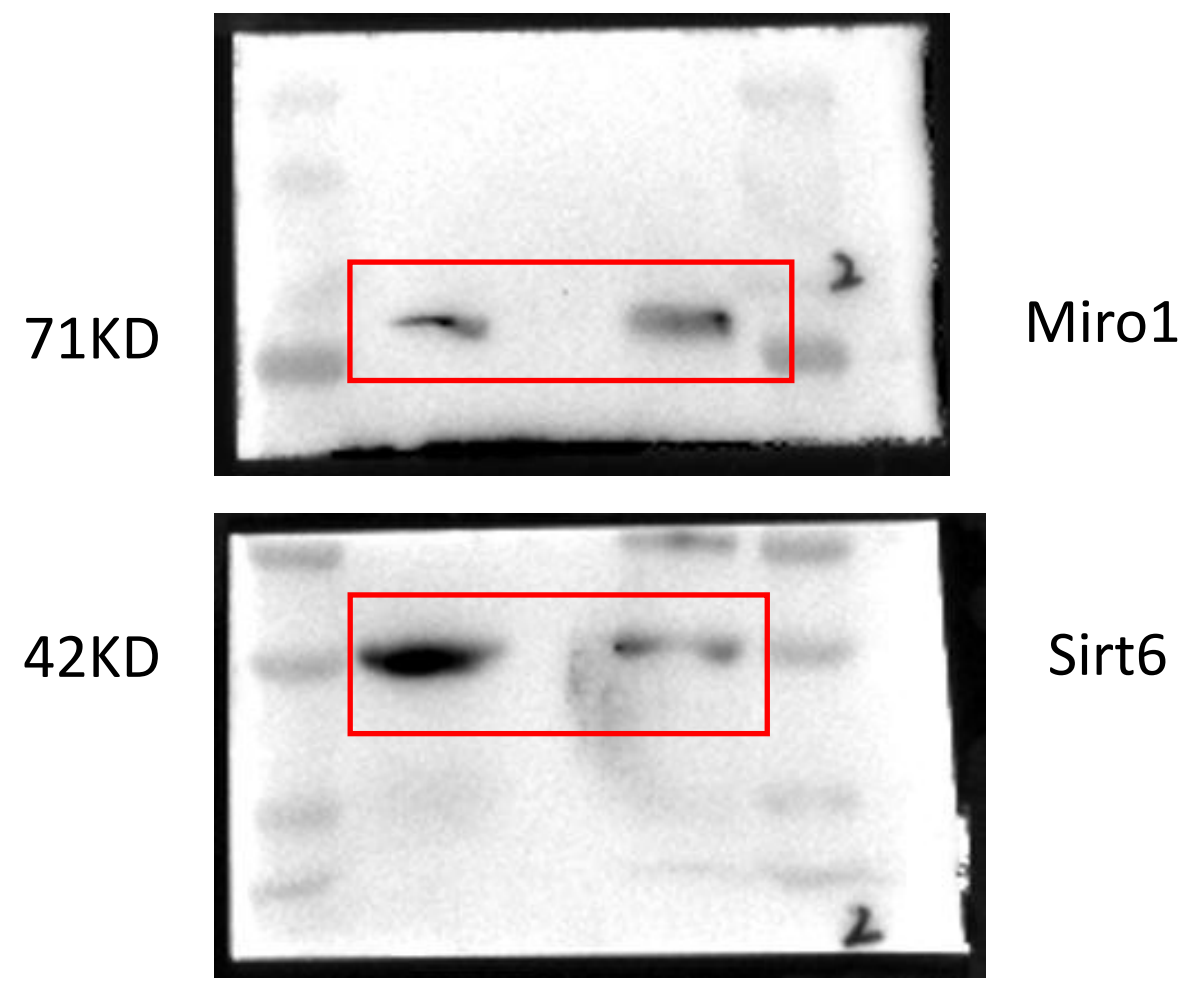

Supplementary Figure 4C

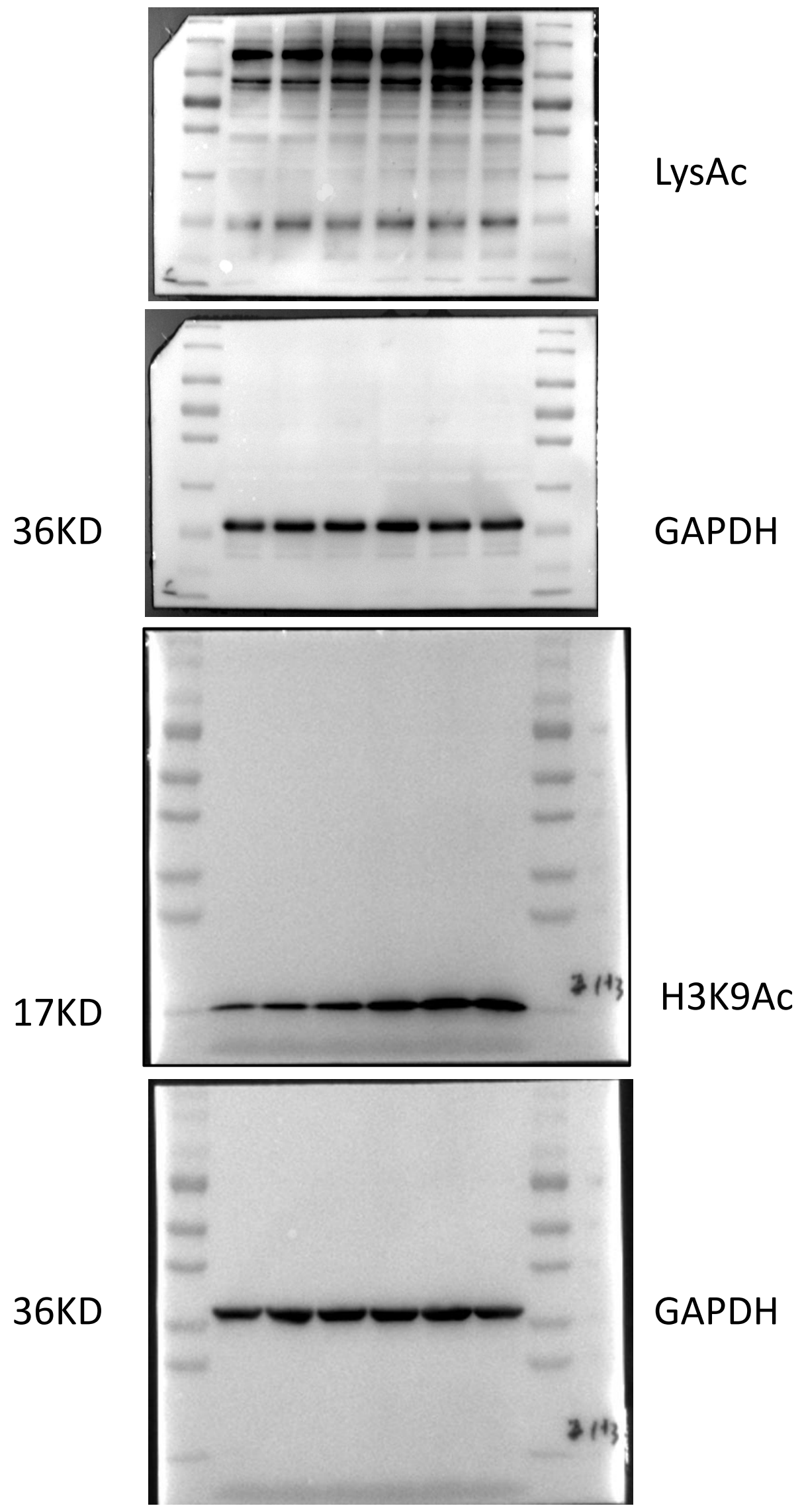

Supplementary Figure 4D

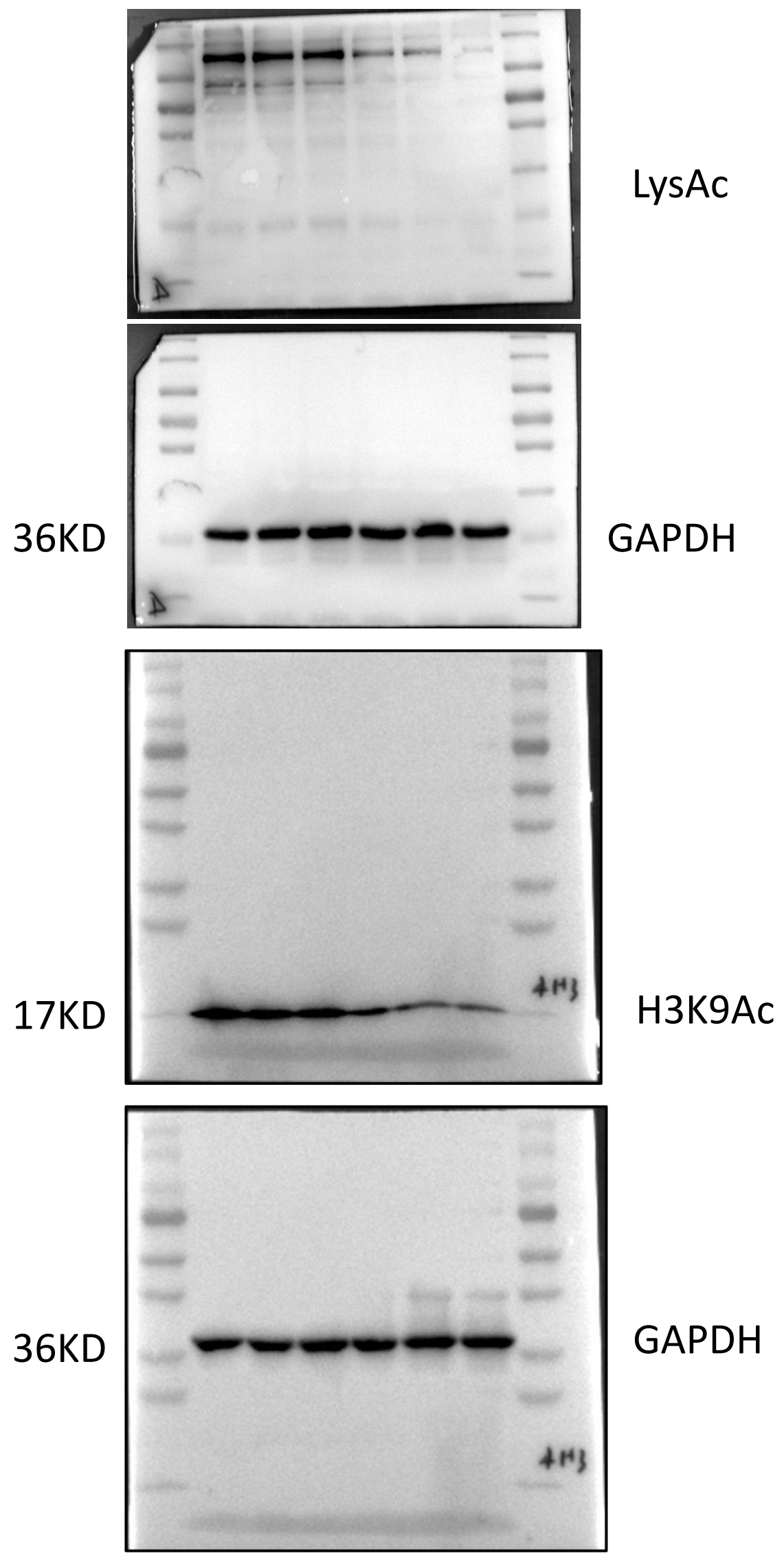

Supplement: Supplementary file 1 [file ADVS-13-e76824-s001.pdf]
